# Cellular crosstalk mediated by Meteorin-like regulating hepatic stellate cell activation during hepatic fibrosis

**DOI:** 10.1038/s41419-025-07734-6

**Published:** 2025-05-20

**Authors:** Yuxia Zhou, Guifang Wang, Lingling Liu, Lingyu Song, Laying Hu, Lu Liu, Lifen Xu, Tuanlao Wang, Lirong Liu, Yuanyuan Wang, Tian Zhang, Bing Guo

**Affiliations:** 1 Department of Pathophysiology, Guizhou Provincial Key Laboratory of Pathogenesis and Drug Research on Common Chronic Diseases, Guizhou Province Talent Base of Research on the Pathogenesis and Drug Prevention and Treatment for Common Major Diseases, Guizhou Medical University, Guiyang, Guizhou China; 2 Department of Pathology, People’s Hospital of Qianxinan Prefecture, Xingyi, Guizhou China; 3 Department of Gastroenterology, Affiliated Hospital of Guizhou Medical University, Guizhou Provincial Key Laboratory for Digestive System Diseases, Guiyang, Guizhou China; 4 Department of Pathology, Affiliated Hospital of Guizhou Medical University, Guiyang, Guizhou China; 5 School of Pharmaceutical Sciences, State Key Laboratory of Cellular Stress Biology, Fujian Provincial Key Laboratory of Innovative Drug Target Research, Xiamen University, Xiamen, Fujian China; 6 Guizhou Institute of Precision Medicine, Affiliated Hospital of Guizhou Medical University, Guizhou Medical University, Guiyang, Guizhou China

**Keywords:** Diseases, Mechanisms of disease

## Abstract

Liver fibrosis is characterized by an excessive accumulation of extracellular matrix (ECM), primarily produced by activated hepatic stellate cells (HSCs). The activation of HSCs is influenced by paracrine signaling interactions among various liver cell types, but molecular mechanisms remain to be elucidated. Secretory Meteorin-like (Metrnl) can effectively ameliorate fulminant hepatitis. However, little is known about its role in liver fibrosis. In our study, we found that hepatic Metrnl mRNA transcripts and protein expression were significantly downregulated in patients and mouse models of hepatic fibrosis. Hepatocyte-specific and global knockout of Metrnl exacerbated CCl4-induced liver fibrosis. In contrast, the administration recombinant Metrnl or AAV-Metrnl overexpression markedly ameliorated CCl4-induced liver fibrosis in mice, suggesting a protective role for Metrnl. Mechanistically, hepatocyte-derived Metrnl not only influences the activation of HSCs through paracrine signaling but also modulates the release of the fibrogenic cytokine PDGFB via the transcription factor EGR1, thereby regulating PDGFB/PDGFRβ signaling to affect HSC activation. Furthermore, Metrnl absence in hepatocytes and HSCs leads to the downregulation of the E3 ubiquitin ligase HECW2, inhibiting K48-linked ubiquitination of FN and preventing its proteasomal degradation, thus promoting FN secretion from HSCs. These effects contribute to ECM deposition and the activation of HSCs, ultimately exacerbating liver fibrosis. Collectively, our study reveals Metrnl as a novel regulator of liver fibrosis that mediates communication between hepatocytes and HSCs, indicating its potential as a therapeutic target for liver fibrosis. The identification of Metrnl as a critical player in the pathogenesis of hepatic fibrosis underscores the importance of understanding cellular crosstalk in the progression of liver disease.

## Introduction

Liver fibrosis, a common pathological process in end-stage liver disease, can be caused by various factors such as hepatitis virus infection, drugs, autoimmune hepatitis, or non-alcoholic fatty liver disease. Advanced liver fibrosis can lead to severe complications like cirrhosis, liver failure, and hepatocellular carcinoma, thereby increasing global morbidity and mortality rates [1–3]. Despite the use of conventional anti-fibrotic drugs, many patients still experience persistent fibrosis. Currently, liver transplantation is the only effective treatment for end-stage liver disease; however, its availability is limited due to donor shortages [4, 5]. Therefore, understanding the mechanisms of liver fibrosis regression could help identify new therapeutic targets.

Multiple cell types, including hepatic stellate cells (HSCs), hepatocytes, Kupffer cells, and liver sinusoidal endothelial cells are involved in liver fibrosis [6, 7]. The activation of latent HSCs is a key event in liver fibrosis and occurs when HSCs interact with other viable and infiltrating hepatocytes [8]. This leads to the production of different inflammatory factors and fibrotic cytokines. Upon microenvironmental stimulation, quiescent HSCs can transform into activated myofibroblast-like cells that exhibit fibrotic characteristics. These activated HSCs express α-smooth muscle actin (α-SMA) and produce excessive extracellular matrix (ECM) proteins, resulting in fibrosis [1, 6, 7]. However, the regulation of HSC activation and the interaction between HSCs and other liver cells are not well understood. Therefore, understanding these interactions is crucial for gaining a deeper understanding chronic liver disease biology and discovering potential therapeutic targets.

Meteorin-like (Metrnl) is expressed in various tissues, such as adipose tissue, skin, liver, and cardiac tissue [9–11], acting as a myokine or adipokine that plays a role in regulating metabolism [9, 12–15]. Recent studies have identified Metrnl as a cardiac factor that helps protect the heart from dysfunction [16–18]. Furthermore, Metrnl deficiency has been linked to decreased blood HDL cholesterol levels under a high-fat diet [19], while overexpression of Metrnl has shown effectiveness in ameliorating fulminant hepatitis [20], indicating a potential role of Metrnl in liver function. Despite these findings, the specific role of Metrnl in regulating liver disease, particularly fibrosis, and the underlying mechanisms of hepatic Metrnl function remain unclear.

This study aimed to investigate the impact of Metrnl in liver disease using CCl4-induced liver injury mouse models with Metrnl global knockout, HSC-specific knockout, and hepatocyte-specific knockout mice, as well as in patients with fibrotic livers. The study also explored the effects of restoring Metrnl expression on liver fibrosis through the administration of Metrnl recombinant protein and gene overexpression. Additionally, the research delved into the potential mechanisms through which Metrnl may regulate liver fibrosis. The findings of this study offer valuable insights into fibrosis control and emphasize the critical role of Metrnl in safeguarding the liver against fibrosis.

## Results

### The expression of Metrnl is decreased in activated HSCs and fibrotic liver

**Fig. 1:**
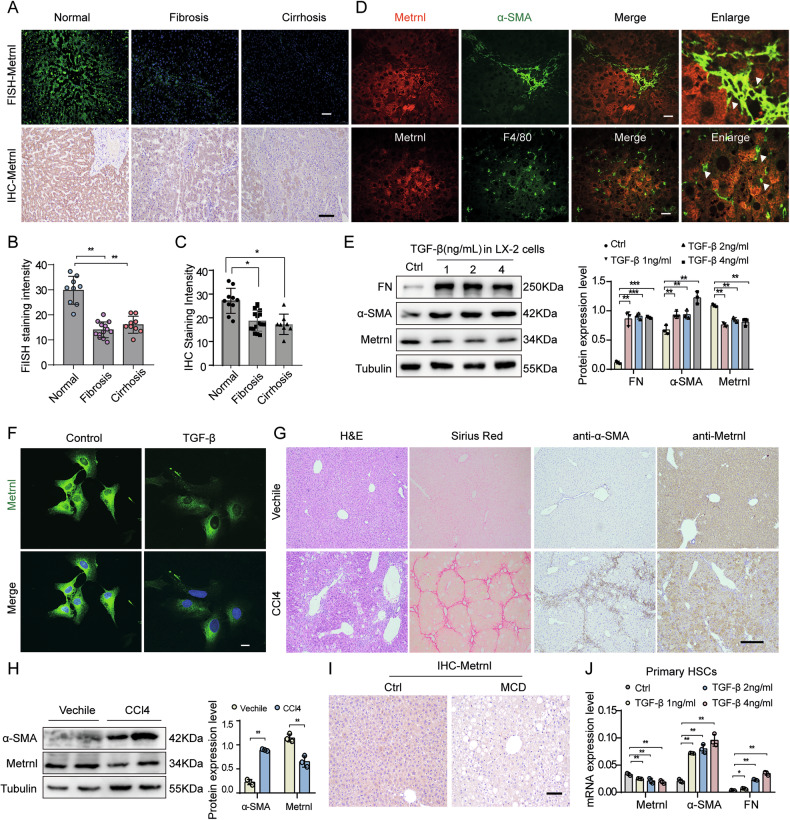
The expression of Metrnl is decreased in activated HSCs and fibrotic liver. Representative IHC staining and FISH staining of Metrnl (**A**), along with quantitative (**B**, **C**) (*n* = 8-15 fields) were conducted on liver tissues from humans with liver fibrosis (*n* = 6), fibrosis cirrhosis (*n* = 5), and healthy controls (*n* = 6). Scale bar, white 50 μm; black, 100 μm. **D** Representative immunofluorescence staining of Metrnl (red) in conjunction with α-SMA and F4/80 (green) were performed on liver tissues from mice subjected to CCl4 induction for 8 weeks. Scale bar, white 50 μm. **E** HSC line LX-2 cells were treated with TGF-β (1, 2, and 4 ng/mL) for 48 h, and the protein levels of FN, α-SMA, and Metrnl were analyzed via Western blotting and quantified (*n* = 3 per group). **F** Immunofluorescence staining of Metrnl was conducted on LX-2 HSC line cells treated with TGF-β (2 ng/mL) for 48 h, with nuclei counterstained using DAPI. Scale bar, white 20 μm. **G** Liver sections from mice treated with CCl4 for 8 weeks underwent H&E staining, Sirus Red staining, and IHC staining for Metrnl and α-SMA (*n* = 6 per group). Scale bar, black, 200 μm. **H** Representative Western blot analysis and quantification were performed to evaluate the protein levels of Metrnl and α-SMA in the liver of CCl4-treated mice (*n* = 3 per group). **I** Representative IHC staining of Metrnl in the liver of mice induced with methionine-choline deficient (MCD) diet for 4 weeks was compared to control mice. Scale bar, black, 100 μm. **J** Primary HSCs isolated from C57BL/6 mice were treated with TGF-β (1, 2, and 4 ng/mL) for 48 h, and the mRNA expression levels of Metrnl, α-SMA, and FN were assessed (*n* = 3 per group). All data are mean ± SD. *P < 0.05, **P < 0.01, and ***P < 0.001.

Previously, we reported that Metrnl exhibits widespread expression across multiple adult tissues, with particularly elevated levels in mouse livers [21]. To investigate its potential implications in the pathogenesis of liver disease, we conducted an initial examination of Metrnl’s expression in both healthy and fibrotic human livers. Utilizing immunohistochemical (IHC) and fluorescence in situ hybridization (FISH) staining techniques, we observed a significant downregulation of hepatic Metrnl protein and mRNA in fibrotic livers compared to their healthy counterparts (Fig. 1A–C). Immunofluorescence analysis of liver tissue from CCl4-induced mice demonstrated that activated hepatic stellate cell (HSC) markers, such as α-SMA, exhibited minimal colocalization with Metrnl, as indicated by the white arrow (Fig. 1D). Furthermore, Metrnl was primarily expressed in hepatocytes. The literature indicates that Metrnl is also secreted by infiltrating monocytes and macrophages; however, using the macrophage marker F4/80, we found that Metrnl expression in macrophages within the liver tissue of CCl4 model mice was nearly absent (Fig. 1D). This suggests that Metrnl is predominantly expressed in hepatocytes and HSCs, closely associated with fibrosis.

To further elucidate the expression of Metrnl in HSC, we observed a significant decrease in Metrnl protein levels in the HSC cell line LX-2 when exposed to recombinant TGF-β (Fig. 1E). Additionally, immunofluorescence studies revealed a reduction in endogenous Metrnl protein expression in LX-2 cells following stimulation with recombinant TGF-β (Fig. 1F). We then extended our analysis to commonly utilized murine models of liver fibrosis induced by CCl4 administration or a methionine-choline deficient (MCD) diet. Our findings demonstrated that an 8-week course of CCl4-induced liver injury in C57BL/6 mice resulted in liver fibrosis, as indicated by α-SMA and Sirius-Red staining (Fig. 1G). Consistent with the decreased Metrnl levels observed in fibrotic livers of patients, IHC staining confirmed the downregulation of hepatic Metrnl in CCl4-induced fibrotic livers of mice (Fig. 1G). This was further corroborated by Western blot analysis (Fig. 1H), which revealed a significant reduction in Metrnl expression in the liver tissues of CCl4-treated groups. Moreover, both Western blot analysis and IHC staining indicated that Metrnl protein level were also decreased in the livers of MCD diet-induced mice compared to the control group (Fig. 1I; Supplementary Fig. 1A). Furthermore, we observed that mRNA levels of Metrnl significantly decreased when primary HSCs isolated from C57BL/6 mice were exposed to recombinant TGF-β (Fig. 1J).

Collectively, these findings strongly suggest that Metrnl expression is decreased in activated HSCs and fibrotic liver, with its primary expression occurring in hepatocytes and HSCs during the fibrotic process, indicating that Metrnl likely plays a pivotal role in the regulation of liver fibrosis.

### Supplementation of Metrnl alleviates CCl4-induced liver fibrosis

**Fig. 2:**
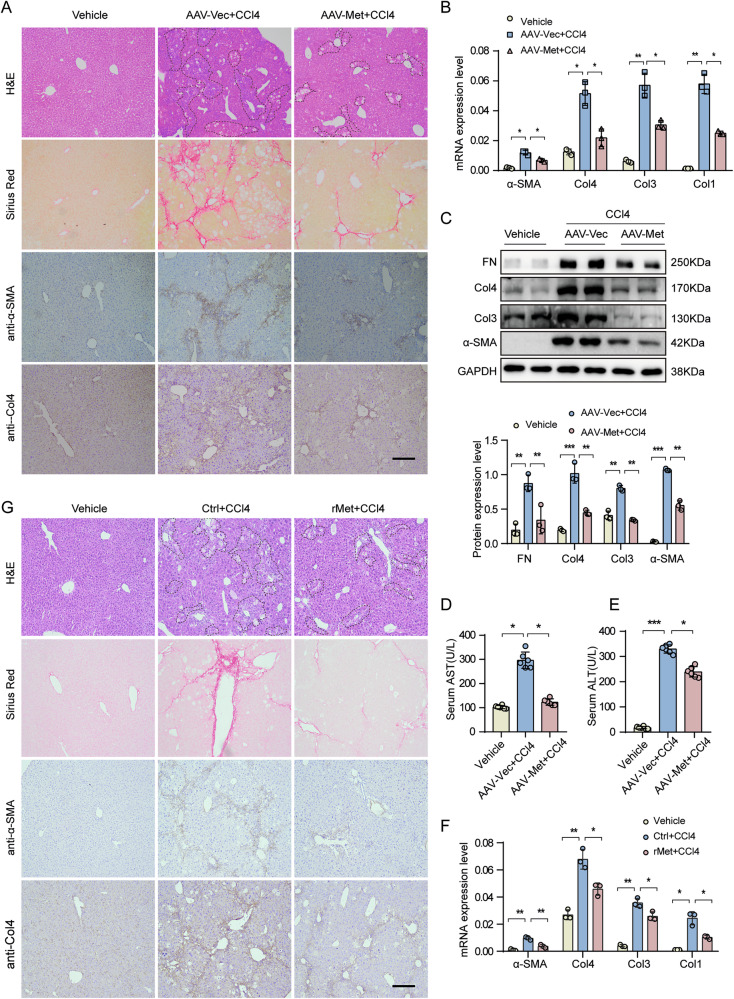
Supplementation of Metrnl alleviates CCl4-induced liver fibrosis. **A** Representative H&E staining, Sirus Red staining, and IHC staining for α-SMA and Col4 of liver sections from CCl4-induced mice conducted with tail vein injection AAV-vector (AAV-Vec) or AAV-Metrnl (AAV-Met) virus (*n* = 6 mice per group). Scale bar, black, 200 μm. **B** The mRNA expression of α-SMA, Col4, Col3, and Col1 in the liver was analyzed in mice injected with AAV-vector or AAV-Metrnl virus, while induced by CCl4 for 8 weeks (*n* = 3 per group). **C** Representative Western blot images and quantification analysis of FN, Col4, Col3, and α-SMA expression in the liver of AAV-vector or AAV-Metrnl mice induced by CCl4 (*n* = 6 per group) (*n* = 3 blots). **D**, **E** Serum AST and ALT levels in CCl4-induced mice injected with AAV-vector or AAV-Metrnl (*n* = 6 per group). **F** The mRNA expression of α-SMA, Col4, Col3, and Col1 in the liver from vehicle or rMetrnl (rMet) treated mice induced by CCl4 for 8 weeks (*n* = 6 per group). **G** Representative H&E staining, Sirus Red staining, IHC staining for α-SMA and Col4 of liver sections from CCl4-induced mice conducted with tail vein injection vehicle or rMetrnl (*n* = 6 per group). Scale bar, black, 200 μm. All data are mean ± SD. *P < 0.05, **P < 0.01, ***P < 0.001.

Our findings indicate a correlation between decreased Metrnl expression and liver fibrosis. To explore the effects of restoring Metrnl expression on liver fibrosis, we generated a liver-targeted Metrnl gene adeno-associated virus vector AAV8-Metrnl, and induced liver fibrosis by injecting CCl4. Treatment with AAV8-Metrnl resulted in Metrnl overexpression in the liver (Supplementary Fig. 1B, C). Notably, the liver tissue subjected to CCl4 exhibited significant infiltration of inflammatory cells, along with flaky and spotty necrosis of hepatocytes. However, AAV8-Metrnl overexpression markedly alleviated both hepatocyte necrosis and inflammatory cell infiltration (Fig. 2A; Supplementary Fig. 1D). Both H&E and Sirius-Red staining showed that Metrnl overexpression alleviated CCl4-induced liver fibrosis compared to the control group (Fig. 2A; Supplementary Fig. 1E). This was also confirmed by IHC staining of α-SMA and Col4 (Fig. 2A). Additionally, the mRNA levels of α-SMA, Col1, Col3, and Col4 in the liver were significantly decreased after Metrnl overexpression (Fig. 2B). Western blot analysis further confirmed the improvement in liver fibrosis (Fig. 2C). Moreover, plasma serum alanine aminotransferase (ALT) and aspartate transaminase (AST) levels were also significantly reduced after Metrnl overexpression in the CCl4-induced liver fibrosis model (Fig. 2D, E), indicating an improvement in hepatic function.

Additionally, recombinant Metrnl (rMet) was administered to C57BL/6 mice via tail vein injection every other day following eight weeks of CCl4 treatment (Supplementary Fig. 1B, F). The results demonstrated that rMet treatment effectively reduced fibrosis and ECM gene expression, as confirmed by RT-qPCR analysis (Fig. 2F). Moreover, H&E staining revealed that rMet significantly alleviated CCl4-induced hepatocyte necrosis (Fig. 2G; Supplementary Fig. 1G). Sirius Red staining and IHC analysis of α-SMA and Col4 further confirmed the attenuation of liver fibrosis in rMet-treated mice (Fig. 2G; Supplementary Fig. 1H). Western blot analysis additionally showed a notable downregulation of myofibroblast markers, including FN, Col4, and α-SMA, in the livers of rMet-treated mice compared to controls (Supplementary Fig. 1I). Collectively, these findings indicate that Metrnl overexpression, either via rMet administration or genetic overexpression, effectively mitigates hepatocyte necrosis and liver fibrosis progression.

### Metrnl deficiency aggravates CCl4-induced liver fibrosis

**Fig. 3:**
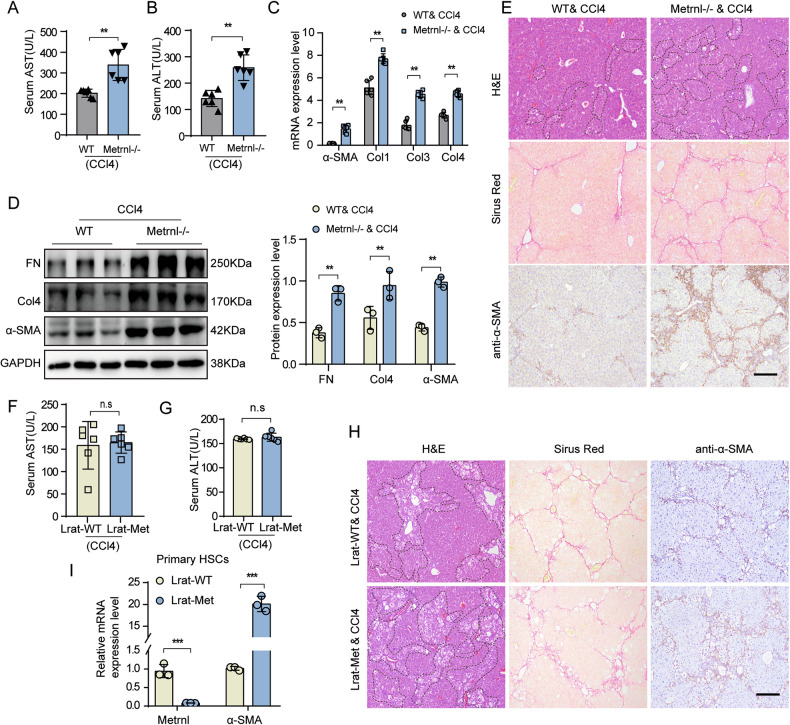
Metrnl deficiency aggravates CCl4-induced liver fibrosis. Serum AST (**A**) and ALT levels (**B**) in WT and Metrnl−/− mice induced by CCl4 for 8 weeks (*n* = 6 mice per group). **C** The mRNA expression of α-SMA, Col1, Col3, and Col4 in the liver from WT and Metrnl−/− mice induced by CCl4 (*n* = 6 mice per group). **D** Representative Western blot images and quantification analysis of FN, Col4, and α-SMA expression in the liver from WT and Metrnl−/− mice induced by CCl4 (*n* = 3 mice). **E** Representative H&E staining, Sirus Red staining, IHC staining of α-SMA of liver sections from WT and Metrnl−/− mice induced by CCl4 (*n* = 6 per group). Scale bar, black, 200 μm. Serum AST (**F**) and ALT levels (**G**) in Lrat-WT and Lrat-Metrnl mice induced by CCl4 for 8 weeks (*n* = 6 mice per group). **H** Representative H&E staining, Sirus Red staining, IHC staining for α-SMA of liver sections from Lrat-WT and Lrat-Met mice induced by CCl4 (*n* = 6 per group). Scale bar, black, 200 μm. **I** Relative mRNA expression of Metrnl and α-SMA in primary HSCs isolated from Lrat-WT and Lrat-Met mice was analyzed after natural differentiation for 7 days, these mRNA levels were normalized to that of GAPDH (*n* = 3 per group). All data are mean ± SD. **P < 0.01, and ***P < 0.001.

To further clarify the role of endogenous Metrnl in liver fibrosis, we utilized global Metrnl knockout mice (Metrnl−/−) and validated the efficiency of the Metrnl gene knockout using qPCR analysis of liver tissue from wild-type (WT) and Metrnl−/− mice (Supplementary Fig. 2A, B). Metrnl−/− mice exhibited significantly elevated ALT and AST levels compared to WT mice following CCl4 injection (Fig. 3A, B). Moreover, the absence of Metrnl led to increased mRNA expression of pro-fibrotic genes (α-SMA, Col1, Col3, and Col4) in the liver (Fig. 3C). Western blot analysis demonstrated an overall upregulation of myofibroblast marker genes in Metrnl-deficient livers relative to WT mice (Fig. 3D). Liver fibrosis was also markedly aggravated in Metrnl−/− mice treated with CCl4, as shown by Sirius-Red staining and IHC staining of α-SMA (Fig. 3E; Supplementary Fig. 2C), indicating more advanced fibrosis compared to WT mice. Additionally, H&E staining showed more seriously hepatocyte necrosis in Metrnl−/− mice treated with CCl4 compared to WT mice (Fig. 3E; Supplementary Fig. 2D). Notably, Metrnl−/− mice without CCl4 treatment did not exhibit significant pathological changes in terms of hepatocyte necrosis (Supplementary Fig. 2E), suggesting that Metrnl’s regulatory role in liver fibrosis may manifest under external stress conditions.

To investigate the impact of HSC-specific Metrnl deletion on liver fibrosis, we created HSC-specific knockout mice by crossing Metrnl^flox/flox^ mice with Lrat-Cre mice to generated Metrnl^f/f;Cre+^ mice, with specific inactivation of the Metrnl gene in HSC cells (referred to as Lrat-Metrnl). Littermate control mice (Metrnl^flox/flox^, denoted Lrat-WT) were used as the control group (Supplementary Fig. 2F). Both Lrat-WT and Lrat-Metrnl mice were injected with CCl4 to induce liver fibrosis. Results showed no significant changes in plasma ALT and AST levels in Lrat-Metrnl mice (Fig. 3F, G). However, CCl4-induced hepatocyte necrosis was slightly aggravated in Lrat-Metrnl mice (Fig. 3H; Supplementary Fig. 2G). Both Sirius-Red staining and IHC staining analysis of α-SMA revealed a modest increase in liver fibrosis in Lrat-Metrnl mice compared to control mice (Fig. 3H; Supplementary Fig. 2H). Interestingly, when primary HSCs from Lrat-Metrnl mice underwent natural differentiation in vitro, the absence of Metrnl significantly enhanced HSC activation, as evidenced by the upregulation of myofibroblast marker genes such as α-SMA (Fig. 3I).

Collectively, these findings suggest that the systemic deficiency of Metrnl significantly contributes to the progression of liver fibrosis. However, in vivo experiments indicate that the absence of Metrnl in HSC only marginally exacerbates liver fibrosis. In contrast, in vitro studies reveal that HSC-specific deletion of Metrnl markedly enhances HSC activation, implying the existence of additional mechanisms that may modulate the impact of HSC-specific Metrnl deletion on liver pathology.

### Metrnl regulates HSCs activation in vitro

**Fig. 4:**
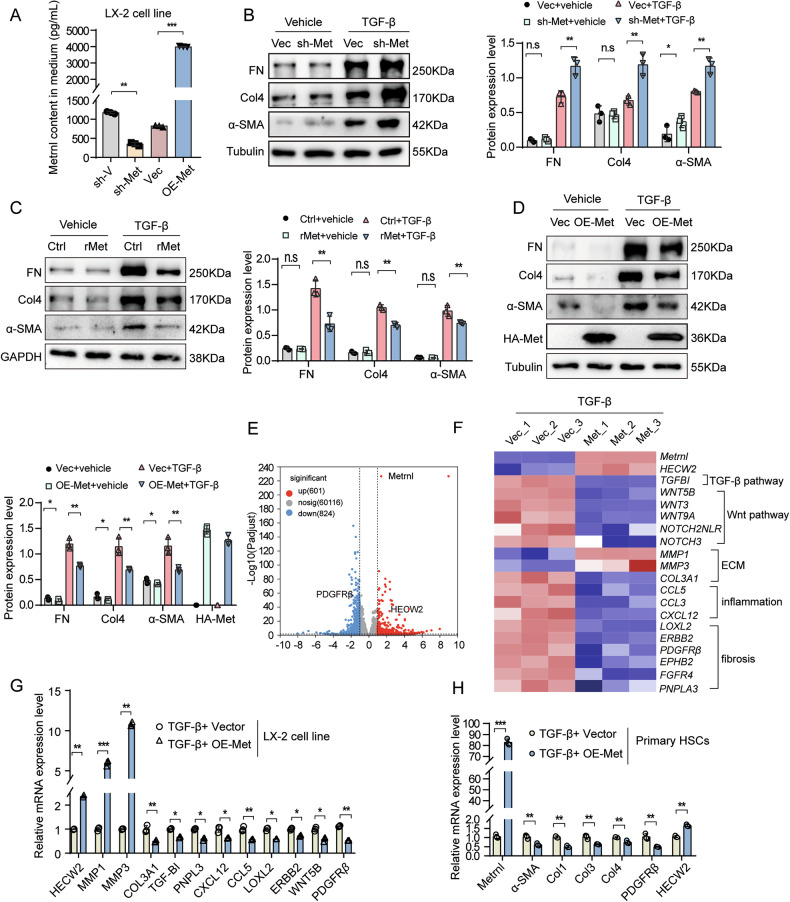
Metrnl regulates HSCs activation in vitro. **A** The level of Metrnl in the cultured medium, secreted by LX-2 cells overexpressing Metrnl (OE-Met) and knockdown Metrnl (sh-Met) induced by adenovirus, was measured using ELISA (*n* = 3 per group). Representative Western blot images and quantification analysis of FN, Col4, and α-SMA expression in LX-2 cells transfected with adenovirus-mediated Metrnl knockdown (**B**), rMetrnl treatment (200 ng/mL) (**C**), and adenovirus-mediated Metrnl overexpression (**D**) combined with TGF-β (2 ng/mL) treatment for 48 h (*n* = 3 blots). **E** Volcano plot analysis was performed on the RNA sequencing results of the indicated samples. A stringent set of genes (*p* < 0.05) was identified in Metrnl overexpressing LX-2 cells compared to vector cells treated with TGF-β for 48 h (*n* = 3 per group). **F** Heat map of representative, differentially expressed genes associated with matrix remodeling, inflammation, and fibrosis is shown. The analysis includes genes involved in fibrosis-related signaling pathways (vector and OE-Met, both with TGF-β, *n* = 3 per group). **G** Changes in relative mRNA levels of genes related to matrix remodeling, inflammation, and fibrosis were observed in Metrnl overexpressing LX-2 cells. These mRNA levels were normalized to that of GAPDH (*n* = 3 per group). **H** Relative mRNA levels of genes related to fibrosis in primary HSCs isolated from C57BL/6 mice and treated with TGF-β (2 ng/mL) for 48 h. These mRNA levels were normalized to that of GAPDH (*n* = 3 per group). All data are mean ± SD. *P < 0.05, **P < 0.01, and ***P < 0.001.

Since activated HSCs are the primary drivers of ECM protein synthesis and deposition, we investigated the role of Metrnl in HSC activation in vitro. To achieve this, we utilized adenovirus-mediated gene expression to overexpress Metrnl in the human HSC line LX-2. Western blot analysis confirmed successful Metrnl overexpression (Supplementary Fig. 1J), while ELISA quantification of Metrnl levels in the culture medium demonstrated its extracellular secretion. Notably, Metrnl overexpression significantly enhanced its secretion, whereas knockdown reduced it (Fig. 4A), validating the effectiveness of both overexpression and knockdown strategies. Furthermore, we observed that Metrnl knockdown significantly enhanced TGF-β induced HSC activation, as shown by increased expression of α-SMA, FN, and Col4 in Metrnl-knockdown LX-2 cells (Fig. 4B). These findings were consistent with our in vitro experiments using primary HSCs isolated from Lrat-Metrnl mice (Fig. 3I). Conversely, treatment with rMet reduced the expression of myofibroblast marker genes in HSCs, leading to the inhibition of HSC activation (Fig. 4C). Adenovirus-mediated Metrnl overexpression in LX-2 cells also suppressed HSC activation under TGF-β treatment (Fig. 4D).

RNA sequencing was conducted to assess the impact of Metrnl overexpression on the HSC transcriptome. Using a significance level of p < 0.05 and a 1.5-fold change threshold, 601 upregulated genes and 824 downregulated genes were identified in Metrnl-overexpressing LX-2 cells treated with TGF-β (Fig. 4E). These genes are primarily associated with ECM remodeling, inflammation, and fibrosis, including key genes in fibrosis-related signaling pathways such as TGF-β and Wnt (Fig. 4F). RT-qPCR validation confirmed alterations in ECM genes (increased MMP1 and MMP3; decreased Col3A1), fibrosis-related genes (decreased TGF-βI, PNPL3, LOXL2, ERBB2, WNT5B, and PDGFRβ), and inflammation-related genes (decreased CXCL12, CCL5) in Metrnl-overexpressing LX-2 cells (Fig. 4G). Furthermore, the genes related to HSCs activation (α-SMA, Col1, Col3, Col4, and PDGFRβ) were inhibited in adenovirus-mediated Metrnl-overexpressing primary HSCs isolated from C57BL/6 mice treated with TGF-β (Fig. 4H).

In summary, our results indicate that Metrnl may modulate the phenotypic transition of HSCs to myofibroblast-like cells, with Metrnl knockdown enhancing HSC activation and overexpression exerting the opposite effect in vitro.

### Hepatocytes-derived Metrnl inhibits HSCs activation through a paracrine manner

**Fig. 5:**
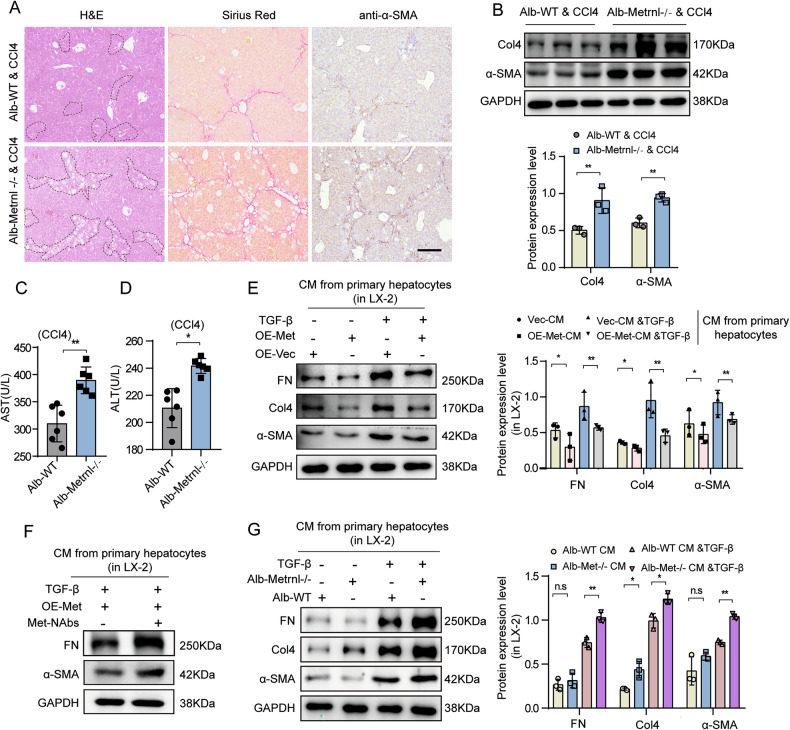
Hepatocytes-derived Metrnl inhibits HSCs activation through a paracrine manner. **A** Representative H&E staining, Sirus Red staining, IHC staining for α-SMA of liver sections from Alb-WT or Alb-Metrnl−/− mice induced by CCl4 for 8 weeks (*n* = 6 mice). Scale bar, black, 200 μm. **B** Representative Western blot images and quantification analysis of Col4 and α-SMA in the liver from Alb-WT or Alb-Metrnl−/− mice induced by CCl4 (*n* = 3 blots). Serum AST (**C**) and ALT levels (**D**) in Alb-WT or Alb-Metrnl−/− mice induced by CCl4 (*n* = 6 mice per group). **E** Representative Western blot images and quantification analysis of FN, Col4, and α-SMA in LX-2 cells after incubating CM from primary hepatocytes transfected with OE-Metrnl under TGF-β (2 ng/mL) stimulation for 48 h. **F** Representative Western blot images of FN and α-SMA in LX-2 cells after incubating CM from primary hepatocytes transfected with OE-Metrnl treated with or without Metrnl neutralizing antibody (Met-Nabs) or control IgG antibody, which was used to block Metrnl in the medium (*n* = 3 blots). **G** Representative Western blot images and quantification analysis of FN, Col4, and α-SMA in LX-2 cells after incubating CM from the medium of primary hepatocyte isolated from Alb-WT or Alb-Metrnl−/− mice, under TGF-β (2 ng/mL) stimulation for 48 h (*n* = 3 blots). All data are mean ± SD. *P < 0.05, **P < 0.01.

As above observed, liver fibrosis development showed a slight increase in HSC-specific Metrnl deletion mice, while significant HSC activation was observed in isolated primary HSCs from Lrat-Metrnl mice in vitro. Given that Metrnl is a secreted protein highly expressed in hepatocytes, we hypothesized that Metrnl originating from hepatocytes might impact liver fibrosis by regulating HSCs activation. To test this hypothesis, we generated mice with hepatocyte-specific Metrnl deletion (Metrnl^flox/flox^, Alb-Cre + , Alb-Metrnl−/−) and matched control mice (Metrnl^flox/flox^, Alb-Cre-, Alb-WT) (Supplementary Fig. 3A, B). Our results demonstrated that Alb-Metrnl−/− mice exhibited increased liver fibrosis compared to Alb-WT mice when subjected to CCl4 treatment. This was confirmed by H&E and Sirius-Red staining, as well as IHC staining of α-SMA and Western blot analysis (Fig. 5A, B). Moreover, both hepatocyte necrosis and the area of interstitial fibrosis were significantly aggravated in Alb-Metrnl−/− mice (Supplementary Fig. 3C, D). Additionally, plasma serum ALT and AST levels were higher in Alb-Metrnl−/− mice injected with CCl4 compared to Alb-WT mice (Fig. 5C, D), suggesting that Metrnl deficiency in hepatocytes significantly contributes to the development of liver fibrosis.

Furthermore, we found that hepatocytes can secrete Metrnl, as detected by ELISA, and the level of Metrnl in the culture supernatant of Metrnl-overexpressing primary hepatocytes was significantly increased (Supplementary Fig. 3E). In co-culture experiments, the activation of LX-2 cells was notably inhibited upon treatment with conditional medium (CM) from Metrnl-overexpressing primary hepatocytes, as confirmed by western blot analysis (Fig. 5E). To evaluate the inhibitory effect of Metrnl secreted from primary hepatocytes on HSC activation, a Metrnl neutralizing antibody was utilized to block Metrnl secretion from Metrnl-overexpressing primary hepatocytes. Results showed that the decreased protein expression of α-SMA and FN induced by the CM from Metrnl-overexpressing primary hepatocytes was significantly reversed by the specific Metrnl neutralizing antibody (Fig. 5F; Supplementary Fig. 3F). Moreover, LX-2 cells exhibited significant activation after treatment with CM from primary hepatocytes isolated from Alb-Metrnl−/− mice compared to Alb-WT control mice (Fig. 5G). These findings suggest that hepatocytes can modulate the release of Metrnl, ultimately aiding in the improvement of liver fibrosis through paracrine signaling. This underscores the pivotal role of Metrnl in mediating communication between hepatocytes and HSCs, thereby ameliorating liver fibrosis.

### Metrnl regulates HSCs activation by targeting the PDGFRβ signaling pathway

**Fig. 6:**
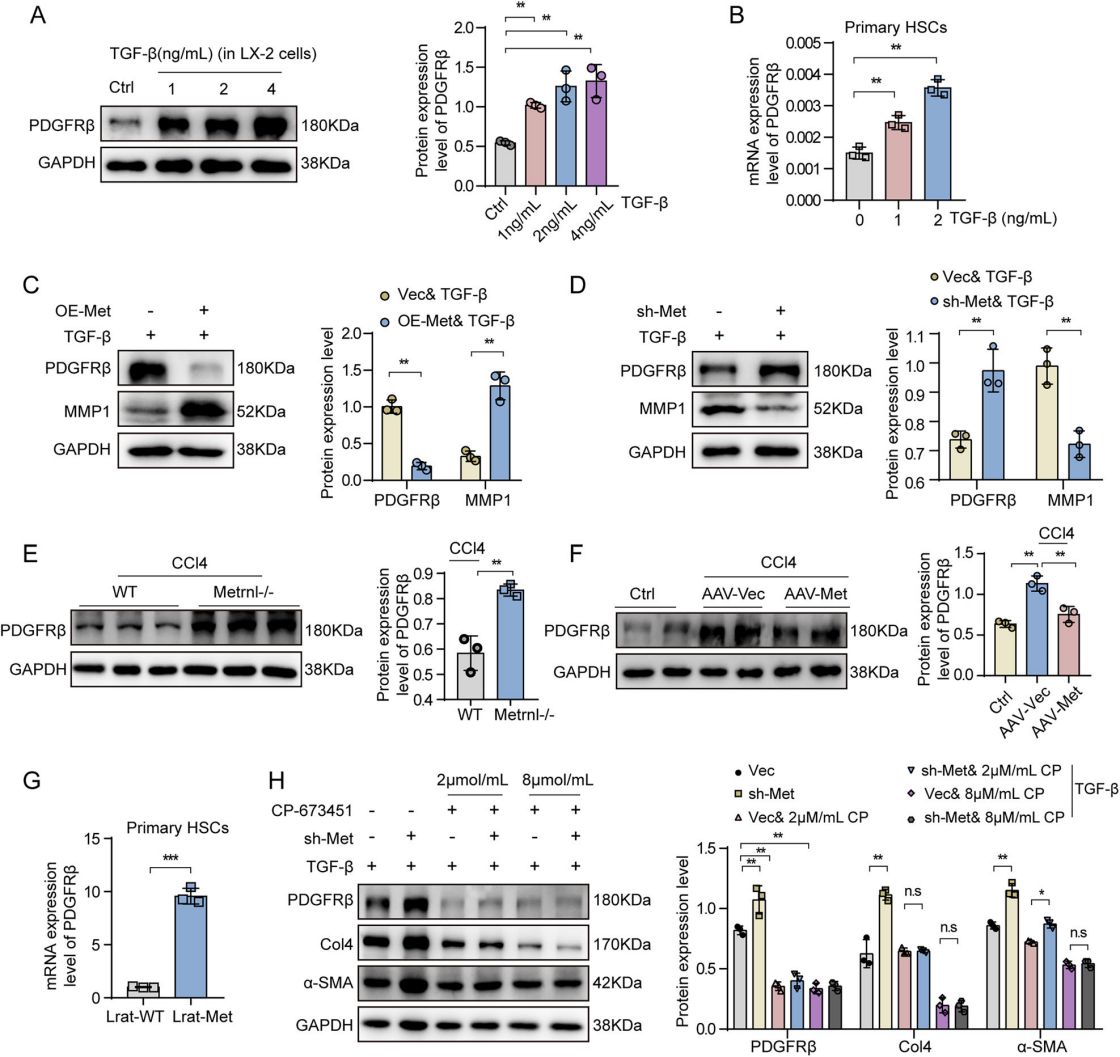
Metrnl regulates HSCs activation by targeting the PDGFRβ signaling pathway. **A** Representative Western blot images and quantification analysis of PDGFRβ expression in LX-2 cells treated with TGF-β (1, 2, and 4 ng/mL) for 48 h (*n* = 3 per group). **B** The mRNA expression of PDGFRβ in primary HSCs isolated from C57BL/6 mice and treated with TGF-β (1 and 2 ng/mL) for 48 h (*n* = 3 per group). Representative western blot images and quantification analysis of PDGFRβ and MMP1 expression in LX-2 cells under TGF-β (2 ng/mL) stimulation for 48 h, either OE-Metrnl overexpression (**C**) or sh-Metrnl knockdown (**D**) (*n* = 3 blots). **E** Representative Western blot images and quantification analysis of PDGFRβ in the liver from WT and Metrnl−/− mice induced by CCl4 for 8 weeks (*n* = 3 blots). **F** Representative Western blot images and quantification analysis of PDGFRβ expression in the liver from CCl4-induced mice injected with AAV-vector or AAV-Metrnl virus (*n* = 3 blots). **G** The mRNA expression of PDGFRβ in primary HSCs isolated from Lrat-WT and Lrat-Met was analyzed after natural differentiation for 7 days (*n* = 3 per group). **H** Representative Western blot images and quantification analysis of PDGFRβ, Col4, and α-SMA expression in LX-2 cells treated with PDGFRβ inhibitor (CP-673451, 2 µM/mL, and 8 µM/mL) and injected with sh-Metrnl knockdown virus, under TGF-β (2 ng/mL) stimulation for 48 h (*n* = 3 blots). All data are mean ± SD. *P < 0.05, **P < 0.01, and ***P < 0.001.

To investigate the impact of Metrnl on HSC activation, we conducted a detailed analysis of RNA-Seq data from Metrnl-overexpressing LX-2 cells treated with TGF-β. Our focus was on PDGFRβ, a key signaling molecule in stellate cells known to play a role in disease progression during chronic liver injury [22]. Our results revealed an increase in both PDGFRβ protein and mRNA levels in LX-2 cells and primary HSCs following TGF-β treatment (Fig. 6A, B). Interestingly, PDGFRβ protein expression decreased significantly by approximately 4-fold in Metrnl-overexpressing LX-2 cells compared to the control group (Fig. 6C), while the opposite effect was observed with Metrnl knockdown (Fig. 6D). Further validation through RNA-Seq data and RT-qPCR confirmed the downregulation of PDGFRβ in LX-2 cells and primary HSCs overexpressing Metrnl (Fig. 4F–H). Moreover, we observed that Metrnl positively regulated MMP1, a key factor involved in ECM degradation, in LX-2 cells (Fig. 6C, D), suggesting a regulatory role of Metrnl in PDGFRβ expression and HSC activation.

In vivo studies also supported these findings, showing increased PDGFRβ levels in the livers of Metrnl−/− mice (Fig. 6E), particularly in the context of CCl4-induced fibrosis. Conversely, Metrnl overexpression led to reduced PDGFRβ expression in the livers of AAV-Metrnl mice (Fig. 6F). Additionally, PDGFRβ mRNA levels were elevated in primary HSCs from Lrat-Metrnl mice (Fig. 6G). Treatment with a PDGFR inhibitor (CP-673451) in LX-2 cells resulted in decreased expression of ECM genes (Col4 and α-SMA) (Fig. 6H), indicating the importance of PDGFRβ signaling in ECM deposition. Importantly, CP-673451 partially reversed the effects of Metrnl knockdown on ECM deposition and HSC fibrosis (Fig. 6H), underscoring the role of Metrnl in regulating HSC activation through the PDGFRβ signaling pathway to mitigate liver fibrosis.

### Metrnl regulates PDGFB release via EGR1 from hepatocytes to activate PDGFRβ signaling pathway

**Fig. 7:**
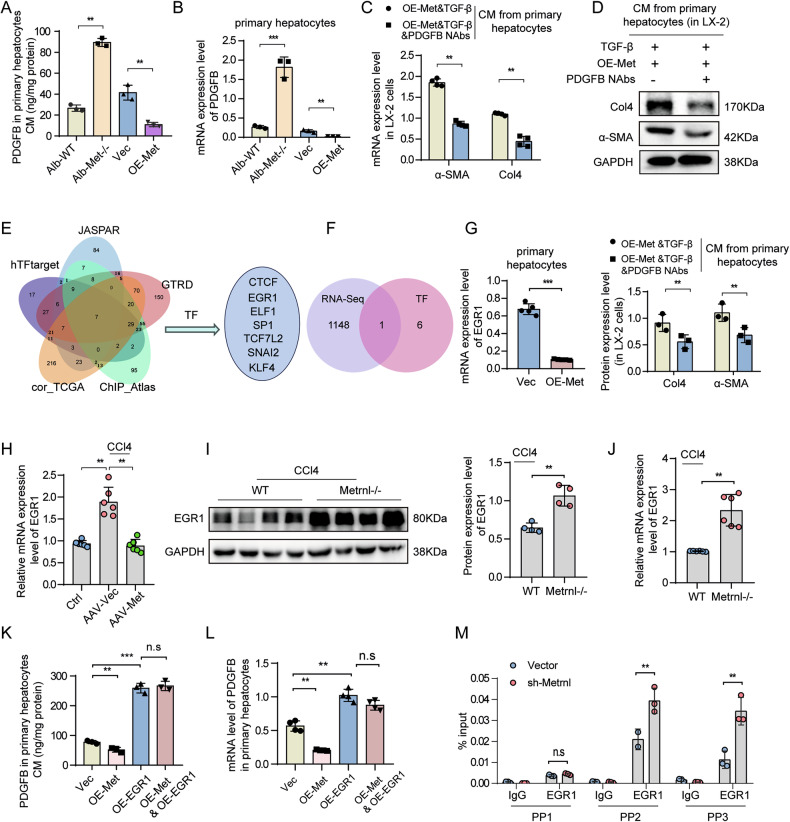
Metrnl regulates PDGFB release via EGR1 from hepatocytes to activate PDGFRβ signaling pathway. ELISA-quantified PDGFB levels in culture medium (CM) (**A**) and mRNA expression level (**B**) of primary hepatocytes transfected with adenovirus-mediated Metrnl overexpression (OE-Met) and primary hepatocytes from Alb-Metrnl−/− mice (*n* = 3 per group). The mRNA expression (**C**) and Western blot images (**D**) and its quantification analysis (down panel) of α-SMA and Col4 in LX-2 cells after incubating CM from LO2 cells transfected with OE-Metrnl virus, and treated with or without PDGFB neutralization antibody (PDGFB-Nabs) or control IgG, which was used to block PDGFB in the CM (*n* = 3 per group). **E** A Venn diagram created by the jvenn tool to display potential transcription factors of PDGFB predicted by TF-Target Finder. **F** A Venn diagram showing the intersection of seven potential transcription factors (CTCF, EGR1, et.al) with RNA-Seq data from Metrnl-overexpressing LX-2 cells. **G** The mRNA expression of EGR1 in primary hepatocytes from C57BL/6 mice transfected with adenovirus-mediated OE-Metrnl constructs (*n* = 5 per group). **H** The mRNA expression of EGR1 in the liver from CCl4-induced mice injected with AAV-vector or AAV-Metrnl virus (*n* = 6 per group). Representative Western blot images (**I**) and quantification analysis of EGR1, also the relative mRNA expression (**J**) in the liver from WT and Metrnl−/− mice induced by CCl4 for 8 weeks (*n* = 4 per group). ELISA-quantified PDGFB levels in CM (**K**) and the mRNA expression levels (**L**) of primary hepatocytes transfected with OE-Metrnl or OE-EGR1 overexpression virus, or both viruses combined (*n* = 3–4 per group). **M** RT-qPCR analysis of PDGFB mRNA levels with EGR1 antibody or IgG antibody respectively by ChIP assay in Metrnl-knockdown or control LO2 cells (*n* = 3 per group). All data are mean ± SD. *P < 0.05, **P < 0.01, and ***P < 0.001.

The above findings indicate that Metrnl negatively regulates both mRNA and protein levels of PDGFRβ in HSCs. This prompts further investigation into whether Metrnl can modulate PDGFB levels, a ligand for PDGFRβ that activates HSCs and contributes to liver fibrosis. Prior research has established that hepatocyte-specific overexpression of PDGFB can induce liver fibrosis [23]. To examine the regulatory effect of Metrnl on PDGFB release in hepatocytes, primary hepatocytes were transfected with adenoviruses carrying either Metrnl overexpression (OE-Metrnl) or knockdown (sh-Metrnl) constructs. The results revealed that Metrnl knockdown led to an increase in PDGFB levels in the culture medium and mRNA levels in primary hepatocytes, while Metrnl overexpression resulted in decreased PDGFB release and mRNA levels (Fig. 7A, B). Notably, treatment with PDGFB neutralizing antibodies (PDGFB Nabs) in the culture medium from primary hepatocytes overexpressing Metrnl significantly reduced the mRNA and protein expression levels of α-SMA and Col4 in LX-2 cells (Fig. 7C, D). This suggests that Metrnl regulates PDGFB expression and release in hepatocytes, thereby influencing the activation state of HSCs.

We further investigated the impact of Metrnl on the regulation of PDGFB expression in hepatocytes. Utilizing the TF-Target Finder tool [24], which integrates databases such as hTFtarget, JASPAR, GTRD, and ChIP-Atlas, we identified seven potential transcription factors (CTCF, EGR1, ELF1, SP1, KLF4, TCF7L2, and SNAI2) that regulate PDGFB expression (Fig. 7E). Further analysis indicated that the expression level of EGR1 was significantly altered in RNA-Seq data from Metrnl-overexpressing cells (Fig. 7F), leading to the hypothesis that Metrnl may influence PDGFB expression through the transcription factor EGR1. Notably, EGR1 mRNA expression was significantly lower in Metrnl-overexpressing primary hepatocytes compared to controls (Fig. 7G). Additionally, an upregulation of EGR1 expression was observed in CCl4-induced mice, suggesting a potential link between EGR1 and the progression of liver fibrosis. This finding is consistent with previous research emphasizing the role of EGR1 in liver fibrosis and NAFLD development [25, 26]. Furthermore, the study demonstrated a significant decrease in EGR1 mRNA expression in the livers of CCl4-induced mice overexpressing Metrnl compared to control mice (Fig. 7H). Furthermore, both EGR1 protein and mRNA levels were notably higher in the livers of CCl4-induced Metrnl−/− mice compared to wild-type mice (Fig. 7I, J). Importantly, in primary hepatocytes with adenovirus-mediated EGR1 overexpression, an increase in PDGFB content was observed (Fig. 7K; Supplementary Fig. 4A), indicating a positive regulatory role of EGR1 in the release of PDGFB from primary hepatocytes. Conversely, EGR1 overexpression reversed the decrease in PDGFB release induced by Metrnl overexpression in primary hepatocytes (Fig. 7K). Additionally, mRNA levels of PDGFB increased in primary hepatocytes with EGR1 overexpression, and EGR1 overexpression counteracted the decrease in PDGFB mRNA levels induced by Metrnl overexpression (Fig. 7L).

ChIP-qPCR analysis demonstrated that the downregulation of Metrnl resulted in increased EGR1 binding to the PDGFB promoter at the PP2 and PP3 binding sites (Fig. 7M). This finding underscores Metrnl’s role as a negative regulator of PDGFB mRNA levels and protein release through the transcription factor EGR1 in hepatocytes. These results suggest that Metrnl inhibits HSCs activation by modulating the PDGFRβ signaling pathway, specifically through the regulation of PDGFB transcription and secretion from hepatocytes via EGR1 expression modulation, ultimately contributing to the amelioration of liver fibrosis.

### Metrnl promotes FN degradation through catalyzing its K48-linked ubiquitination mediated by HECW2

**Fig. 8:**
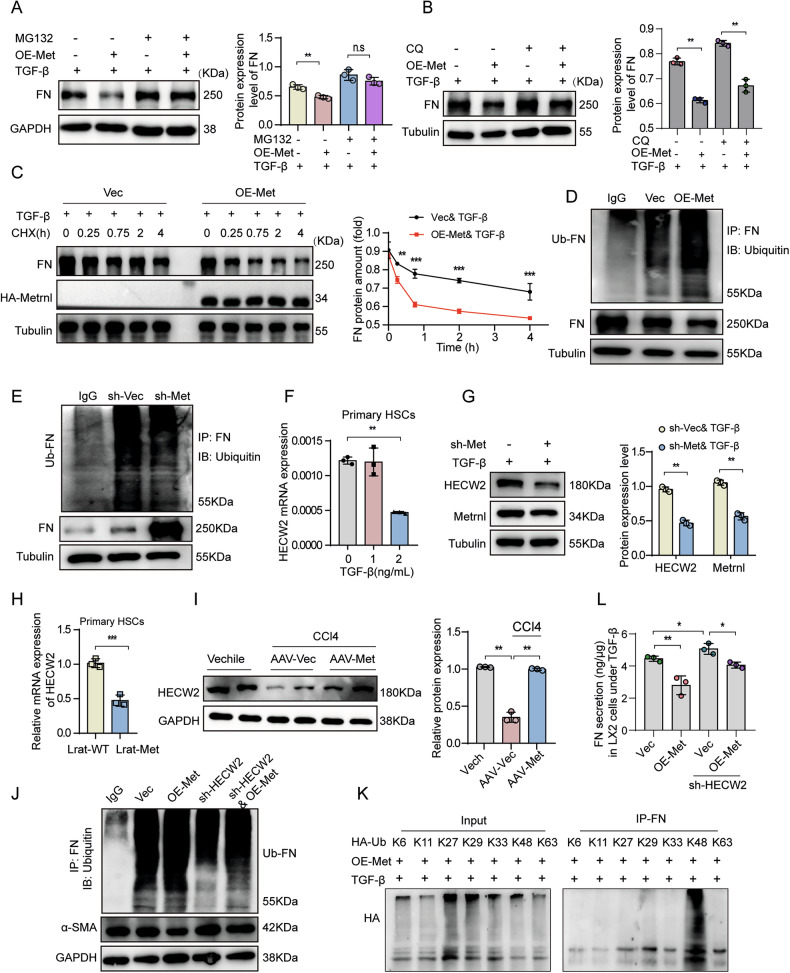
Metrnl promotes FN degradation through catalyzing its K48-linked ubiquitination mediated by HECW2. **A**, **B** Representative Western blot images and quantification analysis of FN protein levels in LX-2 cells treated with MG132 (50 μM) (**A**) or CQ (40 μM) (**B**) for 24 h (*n* = 3 blots). **C** Representative Western blot images and quantification analysis of FN in OE-Metrnl mediated LX-2 cells treated with CHX (50 μM) for indicated time under TGF-β (2 ng/mL) incubation (*n* = 2–3 blots). Co-IP assays of the ubiquitination of FN in OE-Metrnl overexpressing (**D**) or sh-Metrnl knockdown (**E**) LX-2 cells treated with TGF-β (2 ng/mL) for 48 h (*n* = 3 blots). **F** The mRNA expression of HECW2 in primary HSCs isolated from C57BL/6 mice and treated with TGF-β (1 and 2 ng/mL) for 48 h (*n* = 3 per group). **G** Representative Western blot images and quantification analysis of HECW2 and Metrnl in sh-Metrnl knockdown LX-2 cells treated with TGF-β (2 ng/mL) for 48 h (*n* = 3 blots). **H** The mRNA expression of HECW2 in primary HSCs isolated from Lrat-WT and Lrat-Met mice was analyzed after natural differentiation for 7 days (*n* = 3 per group). **I** Representative Western blot and quantification of HECW2 expression in the liver from mice injected with AAV-vector or AAV-Metrnl virus combined with CCl4 (*n* = 3 per group). **J** α-SMA protein expression and ubiquitinated FN level in LX-2 cells transfected with OE-Metrnl overexpression or sh-HECW2 knockdown virus, or both, under TGF-β (2 ng/mL) stimulation for 48 h (*n* = 2 blots). **K** Ubiquitination of FN in LX-2 cells co-transfected with the indicated HA-Ub plasmids and OE-Metrnl overexpression virus, after TGF-β (2 ng/mL) treatment (*n* = 3 blots). **L** ELISA-quantified FN levels in CM from LX-2 cells transfected with OE-Metrnl overexpression or sh-HECW2 knockdown virus, or both, under TGF-β (2 ng/mL) stimulation for 48 h (*n* = 3 per group). All data are mean ± SD. *P < 0.05, **P < 0.01, ***P < 0.001.

Previous studies have demonstrated that FN undergoes post-translational proteolysis in both lysosomes and proteasomes [27–29]. Our study confirmed that FN degradation occurs in both pathways in LX-2 cells; however, the proteasome plays a dominant role, as inhibition of the 26S proteasome with MG132, restored FN protein levels under Metrnl overexpression, but not lysosomal inhibition with chloroquine (CQ) (Fig. 8A, B). Moreover, a cycloheximide (CHX) chase assay demonstrated that Metrnl accelerates FN degradation by shortening its half-life in HSCs (Fig. 8C). Enhanced FN ubiquitination was observed upon Metrnl overexpression (Fig. 8D), while Metrnl knockdown reduced FN ubiquitination (Fig. 8E), indicating that Metrnl promotes FN degradation through the ubiquitin-proteasome pathway.

To further elucidate the molecular mechanism underlying FN ubiquitination, we identified the E3 ubiquitin ligase HECW2 as a key regulator through RNA-seq analysis (Fig. 4E, F). Our data showed a gradual decrease in HECW2 protein levels in LX-2 cells and primary HSCs following TGF-β exposure (Fig. 8F; Supplementary Fig. 4B), suggesting a relation between HECW2 and HSC activation. Notably, Metrnl overexpression upregulated HECW2 mRNA levels in primary HSCs and LX-2 cells (Fig. 4G, H), whereas Metrnl knockdown decreased HECW2 protein expression (Fig. 8G). This reduction in HECW2 mRNA was further validated in primary HSCs isolated from Lrat-Metrnl mice (Fig. 8H). In vivo, the overexpression of Metrnl in CCl4-treated mice resulted in an increase in HECW2 protein levels in the liver, as confirmed by Western blot analysis and IHC staining (Fig. 8I; Supplementary Fig. 4C). Furthermore, the knockdown of HECW2 in LX-2 cells led to a reduction in FN ubiquitination and an elevation in α-SMA expression, indicative of HSC activation (Fig. 8J, Supplementary Fig. 4D). Notably, the deficiency of HECW2 partially reversed the increased FN ubiquitination induced by Metrnl overexpression, resulting in a decrease in FN degradation and promoting HSC activation (Fig. 8J). These findings suggest that Metrnl regulates FN degradation through HECW2-mediated ubiquitination, thereby modulating ECM homeostasis in HSCs.

To further elucidate the type of ubiquitin chain linkage on FN regulated by Metrnl, we transfected LX-2 cells with HA-tagged ubiquitin mutants alongside Metrnl overexpression constructs. Our results demonstrated that FN degradation is mediated via K48-linked ubiquitination (Fig. 8K). Additionally, we observed a decrease in FN secretion in the medium of LX-2 cells overexpressing Metrnl following TGF-β exposure, an effect that was partially reversed by HECW2 knockdown (Fig. 8L). These results reinforce the role of Metrnl in the regulation of FN ubiquitination and secretion. Overall, our findings underscore the critical role of the E3 ubiquitin ligase HECW2 in mediating FN ubiquitination and degradation in HSCs. We demonstrate that Metrnl enhances FN degradation through the ubiquitin-proteasome pathway by upregulating HECW2, ultimately reducing ECM deposition and ameliorating liver fibrosis.

## Discussion

Liver fibrosis is a complex process that develops in response to chronic liver injury, leading to an excessive accumulation of extracellular matrix (ECM) [30]. This abnormal ECM deposition can impair liver function and flexibility, ultimately resulting in liver fibrosis and cirrhosis [7]. Hepatic stellate cells (HSCs) are key players in this process, as they transform into myofibroblasts that contribute to ECM production. However, the mechanisms underlying HSC activation, especially the interplay between hepatocytes and HSCs, which is known to play a role in liver fibrosis, are not fully understood. Therefore, it is essential to identify the factors that regulate this cell-to-cell communication to develop effective therapeutic approaches for the disease.

This study presents compelling evidence from genetic deletion and pharmacological treatment in animal models, indicating that Metrnl plays a crucial role in liver fibrosis. Metrnl facilitates communication between hepatocytes and HSCs, influencing HSC activation and regulating liver fibrosis. The research observed a significant decrease in Metrnl expression in the liver during hepatic fibrogenesis in mouse models induced by CCl4-treatment and MCD diet, as well as in patients with fibrotic livers and activated HSCs. The results indicate that the specific deletion of Metrnl in hepatocytes or the use of a global knockout model exacerbates liver fibrosis. In contrast, genetically overexpressing Metrnl or administering recombinant Metrnl protein can mitigate liver fibrosis in mice. While the impact of HSC-specific deletion of Metrnl on hepatic fibrosis is slightly exacerbated, a notable activation effect on primary HSCs isolated from Lrat-Metrnl KO mice was observed in vitro. Both hepatocyte-specific deletion and global deletion of Metrnl resulted in significant liver injury and fibrosis, suggesting that hepatocyte-derived Metrnl may partially compensate for the effects of HSC Metrnl deletion through paracrine action. This hypothesis was supported by in vitro co-culture experiments and Metrnl antibody neutralization. Importantly, we found that Metrnl was not expressed in infiltrating macrophages within liver tissues from CCl4-treated mice, despite reports of monocyte- and macrophage-derived cytokine Metrnl acting as a driver of post-infarction angiogenesis [31]. Collectively, our findings suggest that hepatocyte-derived Metrnl influences HSC activation via paracrine signaling, establishing Metrnl as a significant signaling molecule in intercellular communication. Other studies have similarly reported that Metrnl is a critical molecule involved in the communication between macrophages and muscle satellite cells [32], vascular endothelial cells and macrophages [31], as well as keratinocytes and vascular endothelial cells [11].

Fibrosis is a complex process involving multiple signaling pathways, with the PDGF/PDGFR signaling pathway playing a significant role in various fibrotic diseases such as myocardial fibrosis, renal fibrosis, liver fibrosis, and skin fibrosis [33, 34]. Activation of PDGFR leads to downstream signal transduction, regulating the activation of HSCs and contributing to the development of fibrotic diseases [35, 36]. In liver fibrosis, both PDGFRα [37] and PDGFRβ are upregulated, with PDGFRα expressed in liver progenitor cells and PDGFRβ mainly expressed in HSCs [38–40]. Decreasing PDGFRβ in HSCs can reduce liver ECM deposition and alleviate liver fibrosis [40]. Our study observed that Metrnl deficiency increased the expression of PDGFRβ, activating HSCs through PDGFB released from hepatocytes via paracrine regulation. Overexpressing Metrnl in hepatocytes reduces PDGFB release, inhibiting HSCs activation through PDGFRβ. Furthermore, we discovered that Metrnl regulates PDGFB release from hepatocytes via the transcription factor EGR1. Previous research has shown that EGR1 is involved in liver fibrosis progression downstream of Elk-3 in CCl4-induced mouse liver fibrotic tissues and human liver cirrhotic tissues [25, 41, 42]. Therefore, hepatocyte-derived Metrnl not only influences the activation of HSCs through paracrine signaling but also modulates the release of fibrogenic cytokine PDGFB, thereby regulating HSC activation. These findings underscore the significant role of Metrnl in the communication between hepatocytes and stellate cells. Furthermore, additional studies have indicated that Metrnl overexpression can ameliorate fulminant hepatitis in mice by inhibiting chemokine-dependent immune cell infiltration [20]. Our study further enhances the understanding of Metrnl’s critical role in liver function protection and suggests that it may serve as an important target for the prevention or treatment of hepatitis and liver fibrosis.

Liver fibrosis is characterized by inflammation and the excessive accumulation of ECM proteins, which disrupt tissue microarchitecture and impair liver function [6]. Fibronectin acts as a scaffold for collagen fibril assembly [43, 44]. Studies have shown that disrupting fibronectin polymerization or genetically ablating it in fibroblasts can reduce liver fibrosis [45]. Inhibiting fibronectin matrix formation, such as with pUR4, has been linked to decreased fibrosis and improved liver function [45–47]. Previous research has emphasized the crucial role of FN as a binding protein for latent TGF-β, activating TGF-β during matrix assembly and influencing HSC activation [48–50]. Ubiquitination is crucial in post-translational modifications of fibronectin, with β-TrCP and HACE1 identified as key regulators of FN degradation and secretion [27–29]. Our study observed both proteasomal and lysosomal degradation of FN in HSCs. However, overexpression of Metrnl in LX-2 cells resulted in a reduction of fibronectin protein expression, primarily by enhancing FN ubiquitination and promoting proteasomal degradation. Furthermore, our research investigates the molecular mechanisms by which Metrnl regulates FN ubiquitination, emphasizing HECW2 as a significant E3 ubiquitin ligase that modulates intracellular FN levels through the K48 ubiquitination proteasomal degradation pathway in HSCs, thereby influencing FN secretion. Because FN is a large protein, we have not been able to identify the site of K48 ubiquitination. The research findings suggest that post-translational modifications play a crucial role in regulating the stability of FN protein in HSCs. Metrnl in hepatocytes and HSCs influences ECM deposition by affecting the levels of ubiquitinated FN, which in turn modulates HSC activation. However, further investigation is required to understand how Metrnl facilitates HECW2-mediated ubiquitination degradation of FN in the liver. While Metrnl has been identified as a ligand of the KIT receptor tyrosine kinase in endothelial cells [31], the specific receptor(s) of Metrnl in hepatocytes and HSCs that control fibrosis remain unknown.

**Fig. 9:**
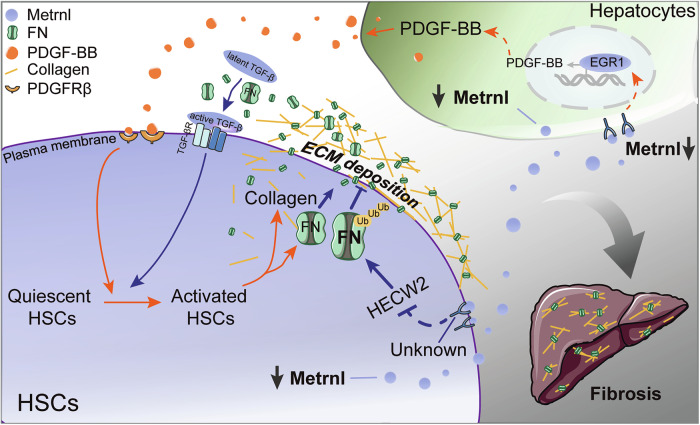
Schematic summary of the possible effect of Metrnl on HSC activation during liver fibrosis. Hepatic Metrnl expression was significantly reduced in the fibrotic liver. The deficiency of Metrnl had a dual effect, resulting in the acceleration of HSC activation. Specifically, the absence of Metrnl in hepatocytes promotes the release of fibrogenic cytokine PDGFB through upregulating transcription factor EGR1, which activates PDGFRβ signaling in HSCs through paracrine regulation. Additionally, the absence of Metrnl in hepatocytes and HSCs results in the downregulation of E3 ubiquitin ligase HECW2, inhibiting K48-linked ubiquitination of FN on proteasomal degradation, thus promoting FN secretion from HSCs. These effects contribute to the deposition of extracellular matrix (ECM) and HSCs activation, ultimately exacerbating liver fibrosis. These findings highlight Metrnl as a novel regulator of liver fibrosis that mediates communication between hepatocytes and HSCs. The mechanistic illustration was created with Adobe Illustrator.

In conclusion, our study demonstrates the significant impact of Metrnl in liver fibrosis. Metrnl deficiency in hepatocytes enhances the secretion of PDGFB by upregulating transcription factor EGR1 expression, leading to PDGFRβ signaling activation in HSCs through paracrine pathways. This cascade accelerates HSC activation and liver fibrosis progression. Moreover, the lack of Metrnl in HSCs downregulates E3 ubiquitin ligase HECW2, impeding K48-linked ubiquitination of FN for proteasomal degradation, thereby promoting FN protein expression, secretion from HSCs. These actions contribute to ECM deposition, HSC activation, and worsen liver fibrosis. Overall, these results shed light on a novel regulatory pathway involving Metrnl that mediates communication between hepatocytes and HSCs, offering insights into potential therapeutic targets for liver fibrosis. The identification of Metrnl as a critical player in the pathogenesis of hepatic fibrosis highlights the importance of understanding cellular crosstalk in liver disease progression. The mechanism of Metrnl in liver fibrosis is illustrated in Fig. 9.

## Materials and methods

### Patient samples

All liver samples used in this study were purchased from Bioaitech Co., Ltd. (Xian, China). Healthy human liver tissues were obtained from individuals who underwent liver operation (*n* = 6). Liver fibrosis (*n* = 6) and cirrhosis tissues (*n* = 5) were obtained from tumor-adjacent tissues of individuals diagnosed with fibrosis and cirrhosis after hepatic carcinoma surgery.

### Mouse models

The mice used in this study were kept in a temperature-controlled environment (24 ± 2 °C) with a 12-h light/dark cycle. Metrnl knockout mice (Metrnl−/− mice) were generated by Shanghai Model Organisms (ShangHai, China). For hepatocyte-specific Metrnl knockout, Metrnl^flox/flox^ mice provided by Gempharmatech were crossed with Alb-Cre transgenic mice provided by Cyagen (JiangSu, China) to generate Metrnl^flox/flox^-Alb-Cre mice (referred to as Alb-Metrnl) and littermate control mice Metrnl^flox/flox,^ (referred to as Alb-WT). For HSC-specific Metrnl knockout, Metrnl^flox/flox^ mice were crossed with Lrat-Cre transgenic mice provided by Cyagen (JiangSu, China) to generate Metrnl^flox/flox^-Lrat-Cre mice (referred to as Lrat-Metrnl) and littermate control mice Metrnl^flox/flox,^ (referred to as Lrat-WT). The genotyping of the Metrnl knockout mice was performed by PCR using the primers listed in Supplementary Table 1.

### Metrnl gene overexpression and rMet treatment intervention of CCl4-induced mice

Adeno-associated virus expressing Metrnl (AAV8-Metrnl) and control virus (AAV8-vector) were delivered into CCl4-induced mice via tail vein injection using an apparatus (Yiyan, China). A total of 100 μL of AAV8-Metrnl or AAV8-vector (1 × 10^11^ pfu mL^−1^) was aspirated using a 29 G insulin syringe. For the experiments involving administration of recombinant protein, rMet (0.3 mg/kg body weight) or a saline solution was administered to CCl4-induced mice through tail intravenous injection every other day for the specified duration.

### Liver fibrosis model

To establish the MCD-induced liver fibrosis model, 8-week-old male C57BL/6 wild type mice were fed either a Methionine- and Choline-Supplement Diet (MCS, cat. TP3006S) or a Methionine- and Choline-Deficient Diet (MCD, cat. TP3006) from Trophic Animal Feed High-Tech Co.,Ltd. (Jiangsu, China) for a duration of 4 weeks. On the other hand, for the CCl4-induced liver fibrosis model, 8-week-old male C57BL/6 wildtype mice were intraperitoneally injected with CCl4 (25%, 3 mL/kg) or Oil twice a week over a span of 8 weeks. In order to study the effect of Metrnl deficiency in CCl4-induced liver fibrosis, 8-week-old male Metrnl−/− mice and their littermate control wildtype mice (WT) were intraperitoneally injected with CCl4 for 8 weeks. Similarly, to obtain a typical liver fibrosis model, Alb-Metrnl or Lrat-Metrnl mice and their littermate control mice underwent the same procedure.

### Antibodies and reagents

Metrnl rabbit polyclonal antibody (WB, 1:1000; IHC and IF,1:200; cat. ab235775) and Fibronectin antibody (WB,1:2000; cat. ab2413) were purchased from Abcam. F4/80 rabbit mAb was purchased from CST (IF, 1:200; cat. D2S9R). α-SMA antibody (IHC/IF,1:200; WB, 1:2000; cat.19245S), Ubiquitin antibody (WB, 1:1000; cat. 43124S) and EGR1 antibody (WB, 1:1000; ChIP,1:50; cat. 4153S) were obtained from CST. PDGFRβ antibody (WB, 1:2000; cat. 13449-1-AP) and MMP1 Rabbit Polyclonal antibody (WB, 1:1000; Cat.10371-2-AP) were purchased from Proteintech. Collagen IV antibody was purchased from Sigma (IHC,1:100; WB, 1:1000; cat. SAB4200500). Rabbit Anti-HECW2 Polyclonal antibody was purchased from Absin (WB, 1:1000; cat. 147321). Anti-collagen III was purchased from Bioss (WB, 1:1000; cat. bs-0948R). The ZS-BIO Kit (Peroxidase, DAB, Rabbit-Mouse, PV-9000) was used for IHC staining. Alexa Fluor^TM^ 488 mouse anti-rabbit (1:200; Invitrogen; cat. A11059) was used for immunofluorescence staining.

### Cell culture and treatments

The human normal hepatocytes cell line LO2 and HSC cell line LX-2 were obtained from Pricella Biotechnology Co., Ltd. They were cultured in high-glucose DMEM medium supplemented with 10% FBS. To induce TGF-β treatment, LX-2 cells were exposed to a TGF-β solution (MCE, China, HY-P7118) with concentrations ranging from 0–4 ng/mL for 48 h.

### RNA in situ hybridization

The mRNA expression of Metrnl in the liver was detected using the FISH detection kit (GenePharma, China), following the manufacturer’s instructions. In summary, the liver tissue arrays were deparaffinized and treated with H_2_O_2_ to expose RNA. Subsequently, the slides were immersed in Target Reagent solution and treated with Protease Plus. The liver tissue arrays were then incubated with a human Metrnl probe mix at 42 °C overnight in a darkroom. Finally, the images were captured using an Olympus Confocal Microscope.

### Relevant indexes detected by ELISA

Blood samples were collected and serum alanine aminotransferase (ALT) and aspartate aminotransferase (AST) levels were measured using a kit from Nan Jing Jan Cheng Biochemical Institute (Nanjing, China). The PDGFB and FN content of the medium supernatant from cells was measured using a commercially available Human PDGFB ELISA Kit (Proteintech; KE100161) and Human Fibronectin/FN1 ELISA Kit (BOSTER; EK0349) individually, following the manufacturer’s instructions.

### Co-immunoprecipitation and Western blot

Cells were lysed using buffer (Beyotime, cat: P0013) containing protease inhibitor and phosphatase inhibitors (MCE; HY-K0010; HY-K0021) for 1 h on ice. The cell lysates were clarified by centrifugation at 14,000 rpm for 30 min. The LX-2 cells lysates were immunoprecipitated with anti-FN antibody and or rotated at 4 °C overnight, followed by addition of 50 μL Protein G Agarose beads (Merck, Cat:3394201) and incubation at 4 °C for 4 h. The proteins bound to Sepharose beads were detected through western blot assay using Ubiquitin antibody.

For Western blot analysis, liver tissue and cells were extracted by homogenizing in RIPA buffer containing protease inhibitor and phosphatase inhibitors. The cell lysates were then separated by SDS-PAGE and the resolved proteins were transferred to a PVDF membrane, Subsequently, the membrane was blocked with 5% milk in TBST, and incubated with primary antibodies, followed by HRP-conjugated secondary antibody. The blots were detected using the ECL system (Tanon; Tanon-5200). Quantitative analysis of protein intensity was performed using Image J software.

### Histology

Freshly harvested mice liver tissues were fixed in 4% paraformaldehyde for 24 h and then embedded in paraffin. Paraffin sections were stained with hematoxylin and eosin (H&E) as well as Sirius Red staining to assess the degree of liver fibrosis. For immunohistochemistry (IHC) staining, liver sections were dewaxed, rehydrated, and subjected to antigen retrieval by boiling in sodium citrate buffer (pH 6.0) for 30 min. After blocking endogenous peroxidase activity and nonspecific binding, the sections were incubated with the primary antibody overnight at 4 °C, followed by incubation with a biotinylated secondary antibody for 30 min. Slides were stained using a DAB substrate and counterstained with hematoxylin for final detection.

### Confocal immunofluorescence microscopy

Immunofluorescence microscopy was conducted following the previously described [51]. Briefly, cells cultured on coverslips were fixed with 3% paraformaldehyde, permeabilized using 0.1% Triton X-100, and subsequently immunostained with primary antibodies. This was followed by incubation with fluorophore-conjugated secondary antibodies. The immunolabeled cells were examined using the FV3000 laser scanning confocal microscope system (Olympus, Japan).

### Co-culture experiment

To investigate the interaction between hepatocytes and HSCs in vitro, we cultured LX-2 cells with the supernatant of LO2 cells overexpressing Metrnl using Ad-Metrnl virus for 48 h. The LX-2 cells were then collected for mRNA and protein analysis. In the PDGFB or Metrnl blockade assay, LX-2 cells were grown in the supernatant of LO2 cells with either Metrnl knockdown or Metrnl overexpression. Additionally, we added 100 ng/ml PDGFB or 50 ng/ml Metrnl neutralization antibody (PDGFB Nabs, Proteintech, 69020-1-lg; Metrnl Nabs, R&D, AF6679,) or control IgG (Mouse IgG, Beyotime, A7028) to the medium every 12 h. After 48 h of culture, the LO2 and LX-2 cells were collected for protein analysis.

### RNA-sequencing analysis

The sequencing of samples involved overexpressing Metrnl LX-2 cells treated with TGF-β for 48 h. These procedures were carried out at Majorbio Bio-Pharm Technology Co., Ltd. (Shanghai, China). Genes were considered significantly differentially expressed if the adjusted *P*-value was found to be less than 0.05 using DESeq2.

### Chromatin immunoprecipitation (ChIP) assay

A ChIP assay was performed using a kit (Cell Signaling Technology, USA). Briefly, DNA-protein complexes were cross-linked with 1% formaldehyde for 15 minutes, followed by isolation of the cross-linked chromatin samples through nuclease digestion. EGR1 was immunoprecipitated using an EGR1 or IgG antibody, and the DNA was subsequently extracted. The ChIP DNA was amplified using primers targeting the PDGFB promoter (Supplementary Table 1) via RT-qPCR. The binding regions of EGR1 and the PDGFB promoter were predicted using the GTRD database, and RT-qPCR primers were designed based on NCBI Primer-BLAST. The values were calculated as a percentage of input using the threshold cycle (2−∆CT) method.

### Quantitative real-time PCR

Total RNA was extracted from tissue or cultured cell samples using TRIzol reagent (Invitrogen, USA). RNA concentration was determined using a NanoDrop One spectrophotometer (ThermoFisher Scientific, USA). The RNA was then converted to cDNA using a PrimeScript Reverse Transcription kit (Takara, China). Quantitative PCR analysis was performed using qPCR SYBR Green Master Mix (Yeasen, China). The relative expression of the target genes was calculated using the 2-ΔΔCT method. The primers used in the study are listed in Supplementary Table 1.

### Primary mouse hepatic stellate cell and hepatocyte isolation

Mouse livers were perfused with a solution of 0.5 mg/mL type IV collagenase (Sigma, USA) in D-Hank’s buffer. To isolate primary hepatic stellate cells (HSCs), the liver tissue was digested and separated using 18% OptiprepTM solution (Axis-shield, cat. 1114542), and the white cell rings at the interface were carefully collected. These rings were centrifuged at 4 °C for 3 min at 800 rpm. For primary hepatocyte isolation, the digested and separated cells were purified using a 90% Percoll solution (Solarbio, cat. P8370). The isolated primary HSCs and hepatocytes were resuspended in DMEM containing 10% FBS for culture.

### Adenovirus-mediated gene expression and knockdown

Recombinant adenovirus was produced using the AdEasy Adenovirus System. The Metrnl gene was cloned into the pAdTrack-CMV vector. The linearized plasmid was then transformed into cells that are more likely to produce the recombinant adenoviral plasmid. These plasmids were subsequently transfected into 293A cells to obtain recombinant adenovirus (hereinafter referred to as Ad-Metrnl). For adenovirus-mediated gene knockdown, the targeting sequences shRNA-Metrnl and shRNA-HECW2 were cloned into the pAdTrack-H1-U6 vector. The recombinant virus was prepared following the same procedure mentioned above. The targeted sequences of shRNA-Metrnl and shRNA-HECW2 can be found in Supplementary Table 1.

### Statistical analysis

All statistical analyses were performed by using GraphPad Prism 8 software, and data were presented as mean ± SD. Statistical differences between two groups were analyzed using the student’s t-test. *P*-value < 0.05 was considered statistically significant.

## Supplementary information

Supplementary Figure legends

Supplementary Figure 1

Supplementary Figure 2

Supplementary Figure 3

Supplementary Figure 4

SupplementaryTable

Western blot Original Data

## Data Availability

The high-throughput sequencing data and original Western blot data have been publicly deposited in the Figshare repository and are accessible at 10.6084/m9.figshare.27977574 and 10.6084/m9.figshare.28637249, respectively. Other original datasets and materials are available from the corresponding author upon reasonable request.

## References

[1] Hernandez-Gea V, Friedman SL. Pathogenesis of liver fibrosis. Annu Rev Pathol. 2011;6:425–56. 21073339 10.1146/annurev-pathol-011110-130246

[2] Parola M, Pinzani M. Liver fibrosis: pathophysiology, pathogenetic targets and clinical issues. Mol Asp Med. 2019;65:37–55. 10.1016/j.mam.2018.09.002 30213667

[3] Lin Y, Dong MQ, Liu ZM, Xu M, Huang ZH, Liu HJ, et al. A strategy of vascular-targeted therapy for liver fibrosis. Hepatology. 2022;76:660–75. 34940991 10.1002/hep.32299 PMC9543235

[4] Zhao M, Wang L, Wang M, Zhou S, Lu Y, Cui H, et al. Targeting fibrosis, mechanisms and cilinical trials. Signal Transduct Target Ther. 2022;7:206–27. 35773269 10.1038/s41392-022-01070-3 PMC9247101

[5] Weiskirchen R. Hepatoprotective and anti-fibrotic agents: it’s time to take the next step. Front Pharm. 2015;6:303–47. 10.3389/fphar.2015.00303 PMC4703795 26779021

[6] Friedman SL. Mechanisms of hepatic fibrogenesis. Gastroenterology. 2008;134:1655–69. 18471545 10.1053/j.gastro.2008.03.003 PMC2888539

[7] Tsuchida T, Friedman SL. Mechanisms of hepatic stellate cell activation. Nat Rev Gastroenterol Hepatol. 2017;14:397–411. 28487545 10.1038/nrgastro.2017.38

[8] Gressner AM, Weiskirchen R, Breitkopf K, Dooley S. Roles of TGF-beta in hepatic fibrosis. Frontiers Biosci. 2002;7:793–807. 10.2741/A812 11897555

[9] Huang S, Cao L, Cheng H, Li D, Li Y, Wu Z. The blooming intersection of subfatin and metabolic syndrome. Rev Cardiovasc Med. 2021;22:799–805. 34565078 10.31083/j.rcm2203086

[10] Miao ZW, Hu WJ, Li ZY, Miao CY. Involvement of the secreted protein Metrnl in human diseases. Acta Pharmacol Sin. 2020;41:1525–30. 32999412 10.1038/s41401-020-00529-9 PMC7921639

[11] Song L, Chang X, Hu L, Liu L, Wang G, Huang Y, et al. Accelerating wound closure with metrnl in normal and diabetic mouse skin. Diabetes. 2023;72:1692–706. 37683051 10.2337/db23-0173

[12] Rao RR, Long JZ, White JP, Svensson KJ, Lou J, Lokurkar I, et al. Meteorin-like is a hormone that regulates immune-adipose interactions to increase beige fat thermogenesis. Cell. 2014;157:1279–91. 24906147 10.1016/j.cell.2014.03.065 PMC4131287

[13] Lee JO, Byun WS, Kang MJ, Han JA, Moon J, Shin MJ, et al. The myokine meteorin-like (metrnl) improves glucose tolerance in both skeletal muscle cells and mice by targeting AMPKalpha2. FEBS J. 2020;287:2087–104. 32196931 10.1111/febs.15301 PMC7383816

[14] Jung TW, Lee SH, Kim H-C, Bang JS, Abd El-Aty AM, Hacımüftüoğlu A, et al. METRNL attenuates lipid-induced inflammation and insulin resistance via AMPK or PPARδ-dependent pathways in skeletal muscle of mice. Experimental Mol Med. 2018;50:1–11. 10.1038/s12276-018-0147-5 PMC6137187 30213948

[15] Li ZY, Song J, Zheng SL, Fan MB, Guan YF, Qu Y, et al. Adipocyte Metrnl antagonizes insulin resistance through PPARg signaling. Diabetes. 2015;64:4011–22. 26307585 10.2337/db15-0274

[16] Ruperez C, Ferrer-Curriu G, Cervera-Barea A, Florit L, Guitart-Mampel M, Garrabou G, et al. Meteorin-like/Meteorin-beta protects heart against cardiac dysfunction. J Exp Med. 2021;218:1–18. 10.1084/jem.20201206 PMC7923691 33635944

[17] Xu L, Cai Y, Wang Y, Xu C. Meteorin-Like (METRNL) Attenuates myocardial ischemia/reperfusion injury-induced cardiomyocytes apoptosis by alleviating endoplasmic reticulum stress via activation of AMPK-PAK2 signaling in H9C2 cells. Med Sci Monit. 2020;26:e924564–75. 32594095 10.12659/MSM.924564 PMC7343023

[18] Lu QB, Ding Y, Liu Y, Wang ZC, Wu YJ, Niu KM, et al. Metrnl ameliorates diabetic cardiomyopathy via inactivation of cGAS/STING signaling dependent on LKB1/AMPK/ULK1-mediated autophagy. J Adv Res. 2022;51:161–79. 36334887 10.1016/j.jare.2022.10.014 PMC10491969

[19] Qi Q, Hu WJ, Zheng SL, Zhang SL, Le YY, Li ZY, et al. Metrnl deficiency decreases blood HDL cholesterol and increases blood triglyceride. Acta Pharmacol Sin. 2020;41:1568–75. 32265491 10.1038/s41401-020-0368-8 PMC7921638

[20] Du Y-n, Teng J-m, Zhou T-h, Du B-y, Cai W. Meteorin-like protein overexpression ameliorates fulminant hepatitis in mice by inhibiting chemokine-dependent immune cell infiltration. Acta Pharmacol Sin. 2023;44:1404–15. 36721008 10.1038/s41401-022-01049-4 PMC10310738

[21] Zhou Y, Liu L, Jin B, Wu Y, Xu L, Chang X, et al. Metrnl alleviates lipid accumulation by modulating mitochondrial homeostasis in diabetic nephropathy. Diabetes. 2023;72:611–26. 36812572 10.2337/db22-0680 PMC10130489

[22] Coentro JQ, Pugliese E, Hanley G, Raghunath M, Zeugolis DI. Current and upcoming therapies to modulate skin scarring and fibrosis. Adv Drug Deliv Rev. 2019;146:37–59. 30172924 10.1016/j.addr.2018.08.009

[23] Czochra P, Klopcic B, Meyer E, Herkel J, Garcia-Lazaro JF, Thieringer F, et al. Liver fibrosis induced by hepatic overexpression of PDGF-B in transgenic mice. Journal Hepatol. 2006;45:419–28. 10.1016/j.jhep.2006.04.010 16842882

[24] Wang J. TFTF: an R-based integrative tool for decoding human transcription factor–target interactions. Biomolecules. 2024;14:749–64. 39062464 10.3390/biom14070749 PMC11274450

[25] Havis E, Duprez D. EGR1 transcription factor is a multifaceted regulator of matrix production in tendons and other connective tissues. Int J Mol Sci. 2020;21:1664–89. 32121305 10.3390/ijms21051664 PMC7084410

[26] Guo Y, Miao X, Sun X, Li L, Zhou A, Zhu X, et al. Zinc finger transcription factor Egf1 promotes non-alcoholic fatty liver disease. JHEP Rep. 2023;5:100724–39. 37234276 10.1016/j.jhepr.2023.100724 PMC10206499

[27] Ray D, Osmundson EC, Kiyokawa H. Constitutive and UV-induced fibronectin degradation is a ubiquitination-dependent process controlled by beta-TrCP. J Biol Chem. 2006;281:23060–5. 16757476 10.1074/jbc.M604311200

[28] McDonald GA, Sarkar P, Rennke H, Unemori E, Kalluri R. VP S. Relaxin increases ubiquitin-dependent degradation of fibronectin in vitro and ameliorates renal fibrosis in vivo. Am J Physiol Ren Physiol. 2003;285:59–67. 10.1152/ajprenal.00157.2002 12820641

[29] El-Hachem N, Habel N, Naiken T, Bzioueche H, Cheli Y, Beranger GE, et al. Uncovering and deciphering the pro-invasive role of HACE1 in melanoma cells. Cell Death Differ. 2018;25:2010–22. 29515254 10.1038/s41418-018-0090-y PMC6219503

[30] Bataller R, Brenner DA. Liver fibrosis. Journal Clin Investig. 2005;115:209–18. 10.1172/JCI24282 PMC546435 15690074

[31] Reboll MR, Klede S, Taft MH, Cai C-L, Field LJ, Lavine KJ, et al. Meteorin-like promotes heart repair through endothelial KIT receptor tyrosine kinase. Science. 2022;376:1343–7. 35709278 10.1126/science.abn3027 PMC9838878

[32] Baht GS, Bareja A, Lee DE, Rao RR, Huang R, Huebner JL, et al. Meteorin-like facilitates skeletal muscle repair through a Stat3/IGF-1 mechanism. Nat Metab. 2020;2:278–89. 32694780 10.1038/s42255-020-0184-y PMC7504545

[33] Klinkhammer BM, Floege J, Boor P. PDGF in organ fibrosis. Mol Asp Med. 2018;62:44–62. 10.1016/j.mam.2017.11.008 29155002

[34] Distler JHW, Gyorfi AH, Ramanujam M, Whitfield ML, Konigshoff M, Lafyatis R. Shared and distinct mechanisms of fibrosis. Nat Rev Rheumatol. 2019;15:705–30. 31712723 10.1038/s41584-019-0322-7

[35] Bonner JC. Regulation of PDGF and its receptors in fibrotic diseases. Cytokine Growth Factor Rev. 2004;15:255–73. 15207816 10.1016/j.cytogfr.2004.03.006

[36] Mekala S, Tulimilli SV, Geesala R, Manupati K, Dhoke NR. Dasa A. Cellular crosstalk mediated by platelet-derived growth factor BB and transforming growth factor β during hepatic injury activates hepatic stellate cells. Can J Physiol Pharm. 2018;96:728–41. 10.1139/cjpp-2017-0768 29558627

[37] Lim BJ, Lee WK, Lee HW, Lee KS, Kim JK, Chang HY, et al. Selective deletion of hepatocyte platelet-derived growth factor receptor alpha and development of liver fibrosis in mice. Cell Commun Signal. 2018;16:93–104. 30509307 10.1186/s12964-018-0306-2 PMC6276164

[38] Chen C, Li X, Wang L. Thymosinbeta4 alleviates cholestatic liver fibrosis in mice through downregulating PDGF/PDGFR and TGFbeta/Smad pathways. Dig Liver Dis. 2020;52:324–30. 31542221 10.1016/j.dld.2019.08.014

[39] Cao S, Yaqoob U, Das A, Shergill U, Jagavelu K, Huebert RC, et al. Neuropilin-1 promotes cirrhosis of the rodent and human liver by enhancing PDGF/TGF-beta signaling in hepatic stellate cells. J Clin Invest. 2010;120:2379–94. 20577048 10.1172/JCI41203 PMC2898590

[40] Kocabayoglu P, Lade A, Lee YA, Dragomir AC, Sun X, Fiel MI, et al. beta-PDGF receptor expressed by hepatic stellate cells regulates fibrosis in murine liver injury, but not carcinogenesis. J Hepatol. 2015;63:141–7. 25678385 10.1016/j.jhep.2015.01.036 PMC4475471

[41] Li TZ, Kim SM, Hur W, Choi JE, Kim J-H, Hong SW, et al. Elk-3 contributes to the progression of liver fibrosis by regulating the epithelial-mesenchymal transition. Gut Liver. 2017;11:102–11. 27538444 10.5009/gnl15566 PMC5221867

[42] Wang Y, Wang P, Yu Y, Huang E, Yao Y, Guo D, et al. Hepatocyte Ninjurin2 promotes hepatic stellate cell activation and liver fibrosis through the IGF1R/EGR1/PDGF-BB signaling pathway. Metabolism. 2023;140:155380–96. 36549436 10.1016/j.metabol.2022.155380

[43] Chiang HY, Korshunov VA, Serour A, Shi F, Sottile J. Fibronectin is an important regulator of flow-induced vascular remodeling. Arterioscler Thromb Vasc Biol. 2009;29:1074–9. 19407246 10.1161/ATVBAHA.108.181081 PMC3091823

[44] Sottile J, Hocking DC. Fibronectin polymerization regulates the composition and stability of extracellular matrix fibrils and cell-matrix adhesions. Mol Biol Cell. 2002;13:3546–59. 12388756 10.1091/mbc.E02-01-0048 PMC129965

[45] Altrock E, Sens C, Wuerfel C, Vasel M, Kawelke N, Dooley S, et al. Inhibition of fibronectin deposition improves experimental liver fibrosis. J Hepatol. 2015;62:625–33. 24946284 10.1016/j.jhep.2014.06.010

[46] Tomasini-Johansson BR, Kaufman NR, Ensenberger MG, Ozeri V, Hanski E, Mosher DF. A 49-residue peptide from adhesin F1 of Streptococcus pyogenes inhibits fibronectin matrix assembly. J Biol Chem. 2001;276:23430–9. 11323441 10.1074/jbc.M103467200

[47] Shi F, Harman J, Fujiwara K, Sottile J. Collagen I matrix turnover is regulated by fibronectin polymerization. Am J Physiol Cell Physiol. 2010;298:C1265–75. 20107040 10.1152/ajpcell.00341.2009 PMC2867395

[48] Dallas SL, Sivakumar P, Jones CJ, Chen Q, Peters DM, Mosher DF, et al. Fibronectin regulates latent transforming growth factor-beta (TGF beta) by controlling matrix assembly of latent TGF beta-binding protein-1. J Biol Chem. 2005;280:18871–80. 15677465 10.1074/jbc.M410762200

[49] Saharinen J, Hyytiäinen M, Taipale J, Keski-Oja J. Latent transforming growth factor-beta binding proteins (LTBPs)–structural extracellular matrix proteins for targeting TGF-beta action. Cytokine Growth Factor Rev. 1999;10:99–117. 10743502 10.1016/s1359-6101(99)00010-6

[50] Robertson IB, Horiguchi M, Zilberberg L, Dabovic B, Hadjiolova K, Rifkin DB. Latent TGF-beta-binding proteins. Matrix Biol. 2015;47:44–53. 25960419 10.1016/j.matbio.2015.05.005 PMC4844006

[51] Zhou Y, Liu Z, Zhang S, Zhuang R, Liu H, Liu X, et al. RILP restricts insulin secretion through mediating lysosomal degradation of proinsulin. Diabetes. 2020;69:67–82. 31624142 10.2337/db19-0086

