# Supplementary material for: Cellular crosstalk mediated by Meteorin-like regulating hepatic stellate cell activation during hepatic fibrosis
Source: Cell Death Dis. 2025 May 20;16(1):405. doi: 10.1038/s41419-025-07734-6 (PMC12092766; doi:10.1038/s41419-025-07734-6)
Supplement: Supplementary file 1 — Supplementary Figure legends [file 41419_2025_7734_MOESM1_ESM.docx]

**Supplementary Figures legends**

**Cellular crosstalk mediated by Meteorin-like regulating hepatic stellate cell activation during hepatic fibrosis**

**Supplementary Fig.1**

**A** Representative Western blot of Metrnl protein in the liver of mice induced with MCD for 4 weeks compared to control mice (n = 4 per group).

**B** 10-week-old C57BL/6 mice were intraperitoneally injected with CCl4 twice a week for 8 weeks. Concurrently, they received either recombinant Metrnl (rMet) or a vehicle (PBS) via tail vein injection every other day for the same duration. In a separate experiment, at 10 weeks of age, mice were injected with AAV8-Metrnl virus via the tail vein. After 4 weeks, mice were then subjected to intraperitoneal CCl4 injections twice a week for an additional 8 weeks.

**C** The mRNA expression of Metrnl in the liver from mice injected with AAV-vector or AAV-Metrnl virus combined with CCl4 (n = 7 per group).

**D, E** The quantification of necrosis area (**D**) and relative Sirius Red positive area (**E**) of the liver from mice injected with AAV-Metrnl virus combined with CCl4 in Fig.2A (n = 6 fields per group).

**F** Representative Western blot and quantification of Metrnl expression in the liver from mice treated with vehicle or rMet combined with CCl4 (n = 3 blots).

**G, H** The quantification of necrosis area and relative Sirius Red positive area of the liver from mice injected with rMet combined with CCl4 in Fig.2G (n = 6 fields per group).

**I** Representative Western blot and quantification of FN, Col4, and α-SMA expression in the liver from mice treated with vehicle or rMet combined with CCl4 (n = 3 blots).

**J** Representative Western blot and quantification of Metrnl expression in LX-2 cells transfected with adenovirus-mediated Metrnl overexpression (n = 3 blots).

**Supplementary Fig.2**

**A** PCR products were utilized to verify the deletion of the Metrnl gene in mouse tail DNA. Wild-type mice showed 694 bp fragments, while homozygous mutant mice exhibited 1048 bp fragments when genomic DNA was amplified using the Metrnl-/- primers (P1/P2) and (P3/P4) listed in Supplementary Table 1.

**B** qPCR was performed on the liver samples of 8-week-old wild-type mice and global knockout Metrnl-/- mice using the primer Mus-Metrnl-/-, as listed in Supplementary Table 1, which indicated the depletion efficiency of Metrnl (n = 4 per group).

**C** The quantification of relative Sirius Red positive area of the liver from mice injected with AAV-Metrnl virus combined with CCl4 in Fig.2A (n = 6 fields per group).

**D** The quantification of necrosis area of the liver from WT and Metrnl-/-mice combined with CCl4 in Fig.3E (n = 6 fields per group).

**E** Representative H&E staining in the liver of WT and Metrnl-/-mice at 8-week-old (n = 6 mice per group). Scale bar, black, 200 μm.

**F** PCR products were used to confirm the deletion of the Metrnl gene in mouse tail DNA. Wild-type mice exhibited 268 bp fragments, while homozygous Loxp mutant mice produced 370 bp fragments from genomic DNA using the 5'arm (Flox) primer. Lrat-Cre mice generated 641 bp fragments from genomic DNA using the Lrat-Cre primer mentioned in Supplementary Table 1.

**G** The quantification of necrosis area of the liver from Lrat-WT and Lrat-Metrnl-/-mice combined with CCl4 in Fig.3H (n = 6 fields per group).

**H** The quantification of relative Sirius Red positive area of the liver from Lrat-WT and Lrat-Metrnl-/-mice combined with CCl4 in Fig.3H (n = 6 fields per group).

**Supplementary Fig.3**

**A** PCR products were utilized to verify the deletion of the Metrnl gene by identifying mouse tail DNA. Wild-type mice displayed 268 bp fragments, while homozygous Loxp mutant mice generated 370 bp fragments from genomic DNA using the 5'arm primer (Flox). Alb-Cre mice generated 390 bp fragments from genomic DNA using the Alb-Cre primer mentioned in Supplementary Table 1.

**B** qPCR of the liver from 8-week-old Alb-WT and Alb-Metrnl mice showed the depletion of Metrnl using the primer Mus-Metrnl-/-(Alb/Lrat), as listed in Supplementary Table 1 (n = 4 per group).

**C** The quantification of necrosis area of the liver from Alb-WT and Alb-Metrnl-/-mice combined with CCl4 in Fig.5A (n = 6 fields per group).

**D** The quantification of relative Sirius Red positive area of the liver from Alb-WT and Alb-Metrnl-/-mice combined with CCl4 in Fig.5A (n = 6 fields per group).

**E** ELISA-quantified Metrnl levels in culture medium from primary hepatocytes infected with OE-Metrnl or vector virus (n = 4 per group).

**F** The quantification analysis of protein expression level in Fig.5F (n = 3 blots).

**Supplementary Fig.4**

**A** Representative Western blot images of primary hepatocytes infected with OE-EGR1 virus for 48 hours.

**B** Western blot images and quantification analysis of HECW2 in LX-2 cells treated with TGF-β (1, 2 and 4ng/mL) for 48 hours (*n* = 3 blots).

**C** Representative IHC staining images of HECW2 of liver sections from CCl4-induced mice conducted with tail vein injection AAV-vector and AAV-Metrnl virus. Scale bar, black, 200 μm.

**D** Gene silencing efficiency of HECW2 by qPCR analysis in LX-2 cells mediated by HECW2 knockdown adenovirus (n= 3 per group).
