# Supplementary figures and images for: Cellular crosstalk mediated by Meteorin-like regulating hepatic stellate cell activation during hepatic fibrosis
Source: Cell Death Dis. 2025 May 20;16(1):405. doi: 10.1038/s41419-025-07734-6 (PMC12092766; doi:10.1038/s41419-025-07734-6)

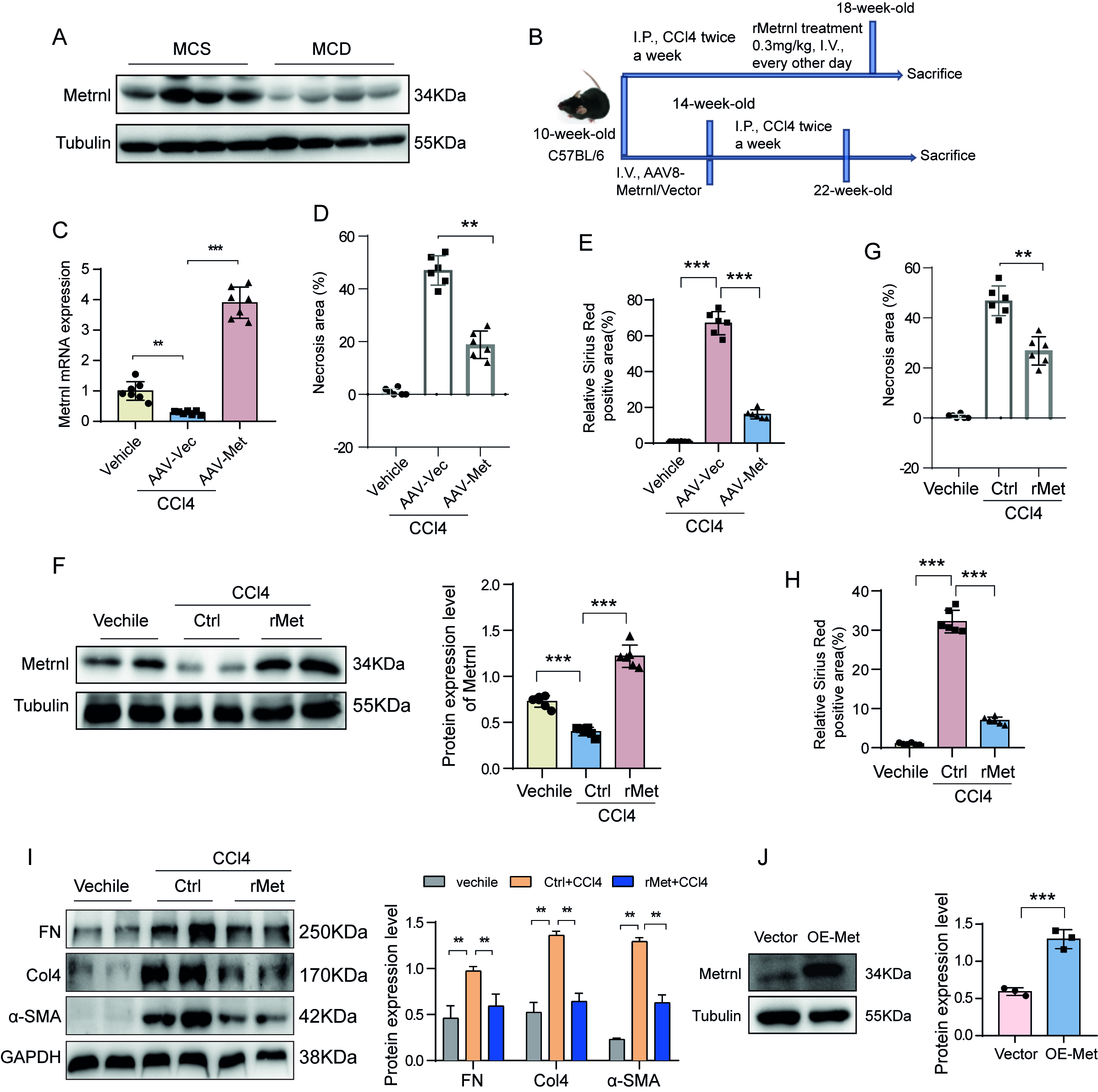

Supplement: Supplementary file 2 — Supplementary Figure 1 [file 41419_2025_7734_MOESM2_ESM.tif]

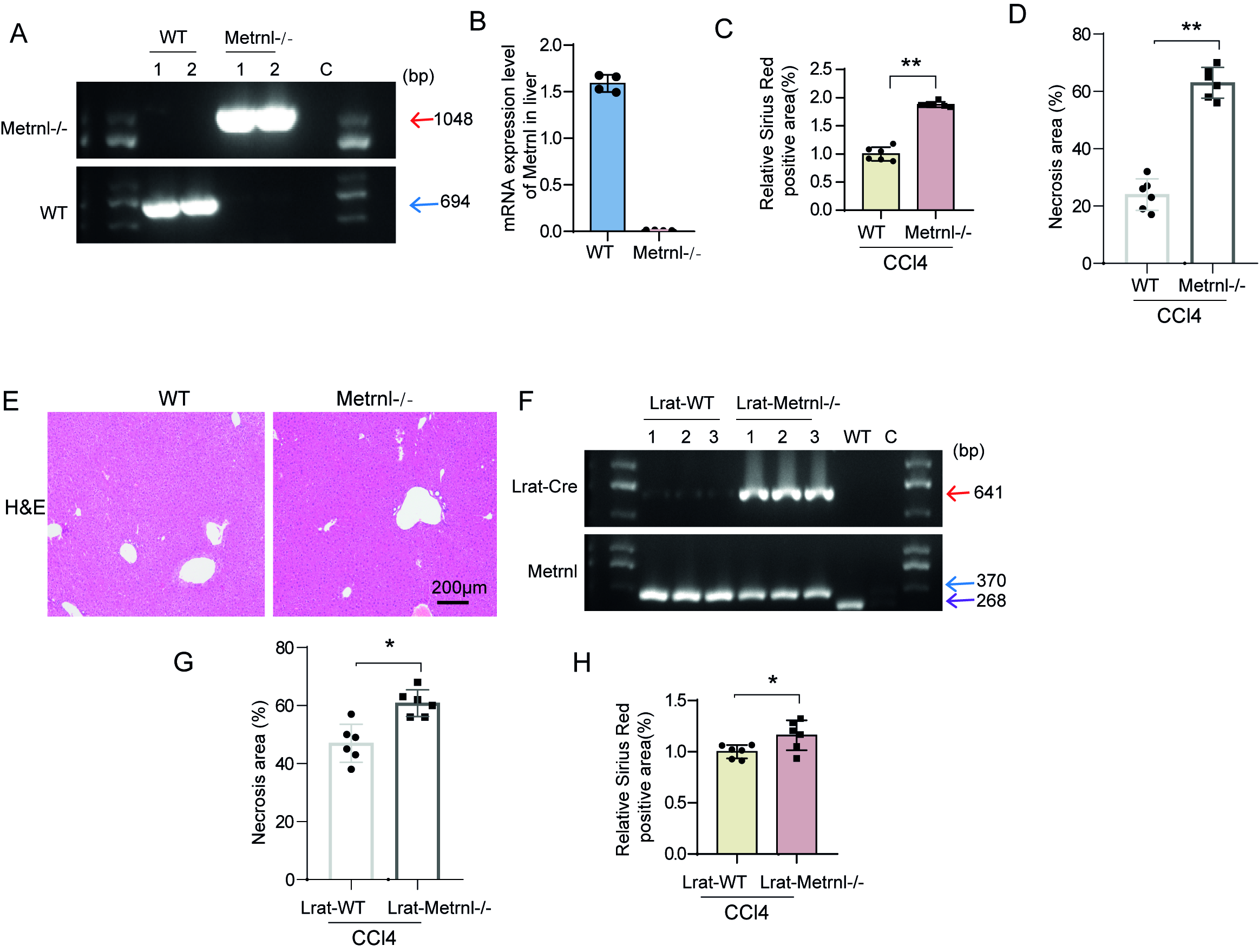

Supplement: Supplementary file 3 — Supplementary Figure 2 [file 41419_2025_7734_MOESM3_ESM.tif]

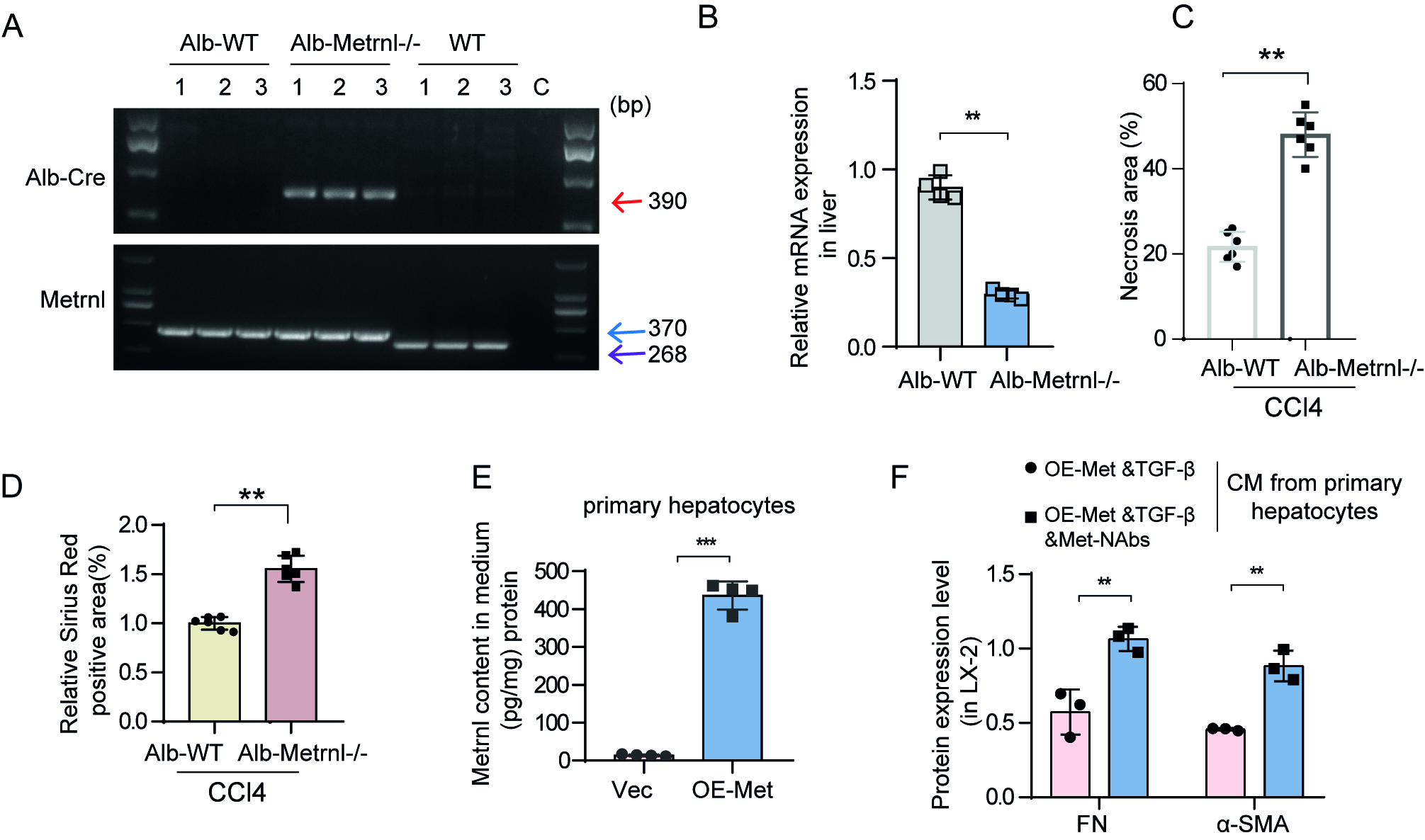

Supplement: Supplementary file 4 — Supplementary Figure 3 [file 41419_2025_7734_MOESM4_ESM.tif]

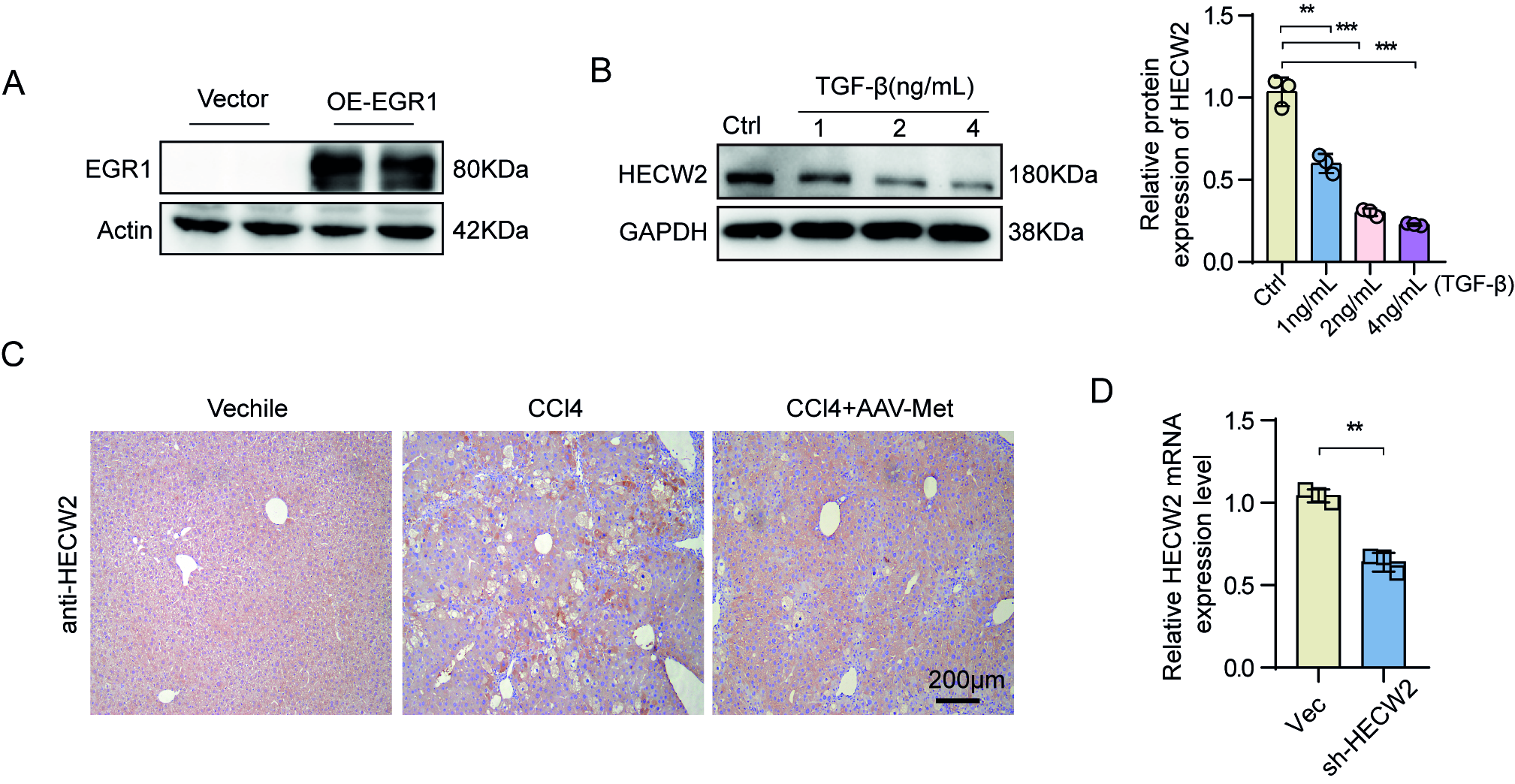

Supplement: Supplementary file 5 — Supplementary Figure 4 [file 41419_2025_7734_MOESM5_ESM.tif]
