# Supplementary material for: Cellular crosstalk mediated by Meteorin-like regulating hepatic stellate cell activation during hepatic fibrosis
Source: Cell Death Dis. 2025 May 20;16(1):405. doi: 10.1038/s41419-025-07734-6 (PMC12092766; doi:10.1038/s41419-025-07734-6)
Supplement: Supplementary file 6 — SupplementaryTable [file 41419_2025_7734_MOESM6_ESM.docx]

**Supplementary Table**

**Cellular crosstalk mediated by Meteorin-like regulating hepatic stellate cell activation during hepatic fibrosis**

**Supplementary Table 1. Primers for real time-qPCR, PCR, and shRNA.**

| **Gene** | **Forward (5' to 3')** | **Reverse (5' to 3')** |
| --- | --- | --- |
| Homo-a-SMA | GATGGTGGGAATGGGACAAA | GCCATGTTCTATCGGGTACTTC |
| Homo-Collagen4 | GTTACAAGGTGTCATTGGGTTTC | GGTAGTCCTGGTTCTCCAGTAT |
| Homo-Collagen1 | CAGACTGGCAACCTCAAGAA | CAGTGACGCTGTAGGTGAAG |
| Homo-Collagen3 | GGAGCTGGCTACTTCTCGC | GGGAACATCCTCCTTCAACAG |
| Homo-PDGFRβ | AGACACGGGAGAATACTTTTGC | AGTTCCTCGGCATCATTAGGG |
| Homo-HECW2 | GGATCCTTGGTCTTGCACTAATA | AGGTCACTCAGGTCACATAGA |
| Homo-MMP1 | CTCTGACATTCACCAAGGTCTC | GATTTCCTCCAGGTCCATCAAA |
| Homo-MMP3 | CAGGCTTTCCCAAGCAAATAG | CTCCAACTGTGAAGATCCAGTAA |
| Homo-TGFβI | CACTCTCAAACCTTTACGAGACC | CGTTGCTAGGGGCGAAGATG |
| Homo-CXCL12 | ATTCTCAACACTCCAAACTGTGC | ACTTTAGCTTCGGGTCAATGC |
| Homo-CCL5 | AGCTTTGTCACCCGAAAGAA | ACTCTCCATCCTAGCTCATCTC |
| Homo-LOXL2 | CTATGACCTGCTGAACCTCAAT | ATTCTTCTGGATGTCTCCTTCAC |
| Homo-ERBB2 | TGTGACTGCCTGTCCCTACAA | CCAGACCATAGCACACTCGG |
| Homo-WNT5B | GCTTCTGACAGACGCCAACT | CACCGATGATAAACATCTCGGG |
| Homo-GAPDH | TGCACCACCAACTGCTTAG | GGATGCAGGGATGATGTTC |
| Homo-PP1 | ATCTTCCCGTCAGTCACCCT | GGGCAGAAGGCGTTAACTGT |
| Homo-PP2 | AATGAAAAATGGGCGCTGGC | AGCTGAAAGGGTGGCAACTT |
| Homo-PP3 | GGGAATGAAAAATGGGCGCT | GCTGAAAGGGTGGCAACTTC |
| Homo-PDGFB | CCAAACTCGGGTGACCATT | CTTCAGTGCCGTCTTGTCAT |
| Homo-EGR1 | GGTCAGTGGCCTAGTGAGC | GTGCCGCTGAGTAAATGGGA |
| shRNA-Metrnl  (Homo) | CAAGGACTTCCAGAGGATGT |  |
| shRNA-HECW2  (Homo) | GCTCCACTCTGGAAATAGACA |  |
| Mus-actin | GGCTGTATTCCCCTCCATCG | CCAGTTGGTAACAATGCCATGT |
| Mus-PDGFRβ | CCAGTGACAGACTACCTCTTTG | CAAGTATAGGTGCCCGAATCA |
| Mus-HECW2 | CTTCATGGAACCGGAGATCAA | GCTGGGTTCTTCACGGTAATA |
| Mus-Collagen3 | CCTGGTGGAAAGGGTGAAAT | CGTGTTCCGGGTATACCATTAG |
| Mus-Collagen4 | CTCACTGTGGATCGGCTATTC | CGCTTCTAAACTCTTCCAGACA |
| Mus-Collagen1 | CCTGGCAAAGACGGACTCAAC | GCTGAAGTCATAACCGCCACTG |
| Mus-a-SMA | GTCCCAGACATCAGGGAGTAA | TCGGATACTTCAGCGTCAGGA |
| Mus-FN | GAAGTCGCAAGGAAACAAGC | GTAGGTGAACGGGAGGACAC |
| Mus-Metrnl | GGAATTCCTCTTCACTGGACAT | CTTCTGCTTTCCTGTACATCCT |
| Mus-GAPDH | AGGTCGGTGTGAACGGATTTG | TGTAGACCATGTAGTTGAGGTCA |
| Mus-EGR1 | TTCAATCCTCAAGGGGAGCC | GAGAAGCGGCCAGTATAGGT |
| Mus-Metrnl-/- | TATTTGTGGAGGCGACACCC | CTGGTACAGATGGCAAGGAGG |
| Mus-Metrnl-/-(Alb/Lrat) | TCCTTGCCATCTGTACCAGTGAC | TCTTCTGCCTGTGAAGCCTGTTC |
| Metrnl-/- (P1/P2) | CCTCCCCAAGGGATGTTTCC | GCTGGACCCCAGCTTAATGT |
| Metrnl-/- (P3/P4) | GGGAGGCAGGCCTTTTGTAT | GCTGGACCCCAGCTTAATGT |
| **5'**arm (Flox) | CGACAAATGGTCAGATCATTGCC | AGGGCACCAGAGTCTTAGTAGCTTC |
| Alb-Cre | GAAGCAGAAGCTTAGGAAGATGG | TTGGCCCCTTACCATAACTG |
| Lrat-Cre | CGGGTGGACACAGAACAATCG | ACCGACGATGAAGCATGTTTAGCTG |
