# Supplementary material for: Cellular crosstalk mediated by Meteorin-like regulating hepatic stellate cell activation during hepatic fibrosis
Source: Cell Death Dis. 2025 May 20;16(1):405. doi: 10.1038/s41419-025-07734-6 (PMC12092766; doi:10.1038/s41419-025-07734-6)

Fig.1

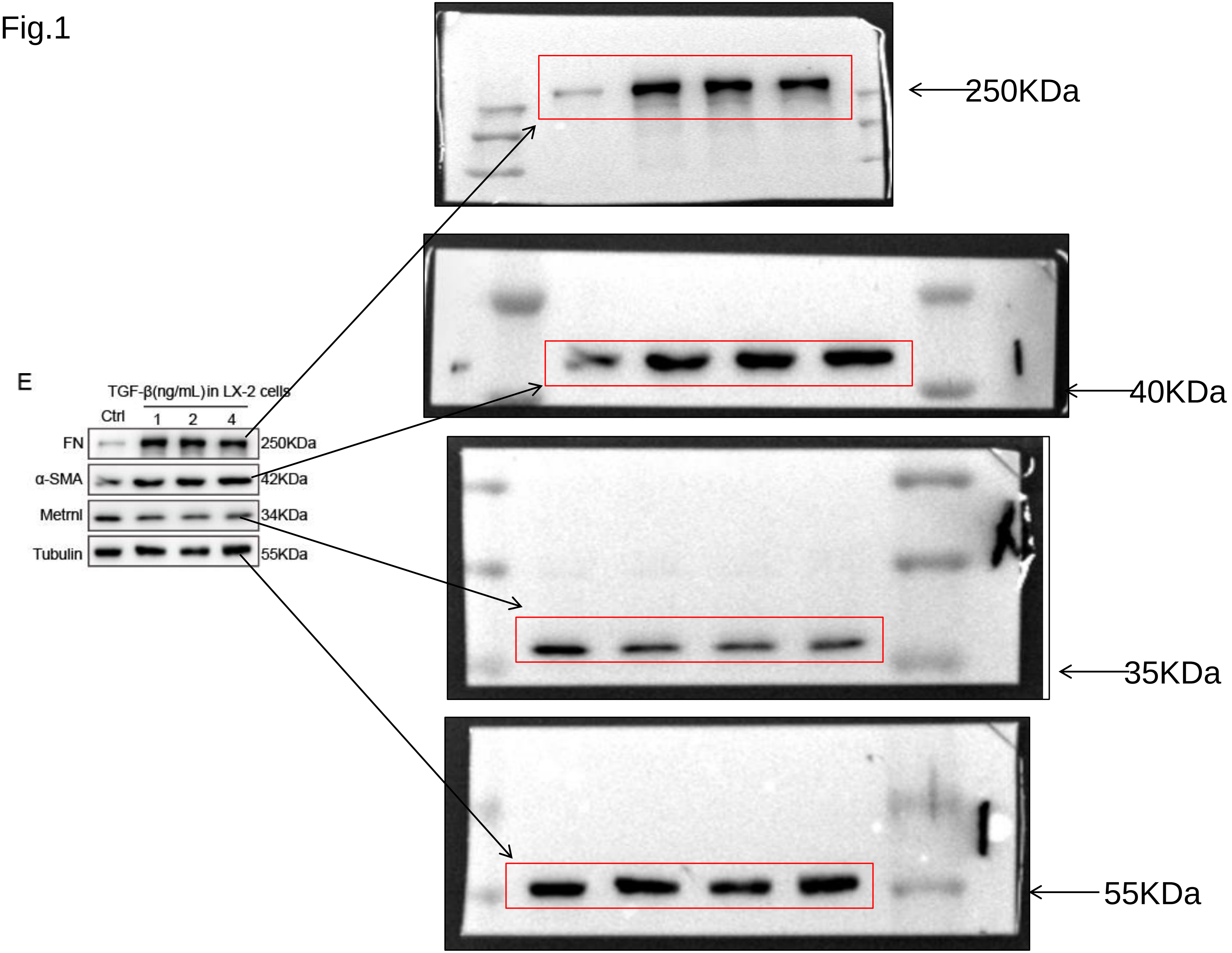

E
TGF-β(ng/mL)in LX-2 cells
Ctrl
1
2
4
FN
250KDa
α-SMA
42KDa
Metrnl
34KDa
Tubulin
55KDa
250KDa
40KDa
35KDa
55KDa

Repeat

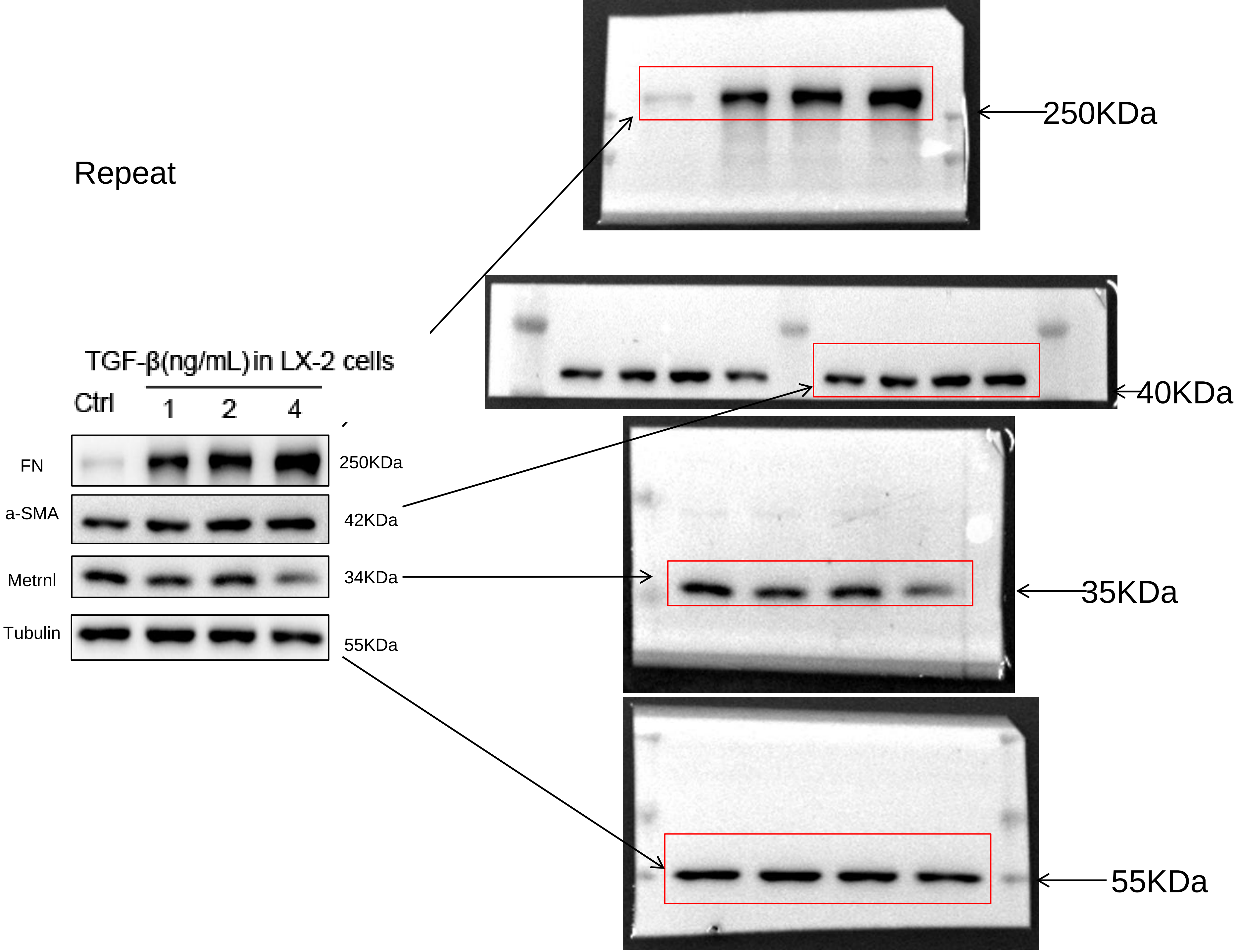
250KDa
40KDa
35KDa
55KDa
TGF-β(ng/mL)in LX-2 cells
Ctrl
1
2
4
FN
250KDa
a-SMA
42KDa
Metrnl
34KDa
Tubulin
55KDa

Repeat

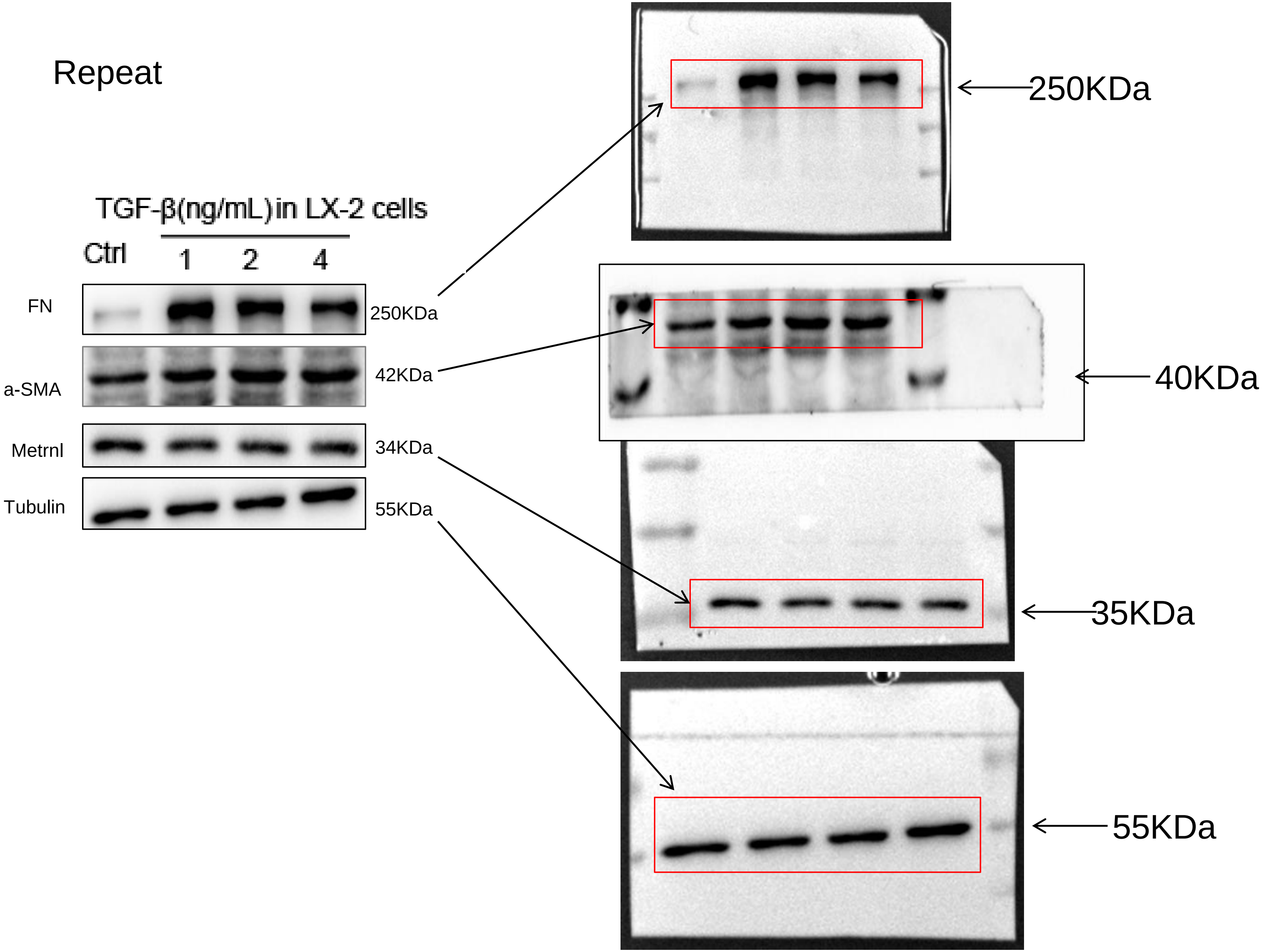
TGF-β(ng/mL)in LX-2 cells
Ctrl
1
2
4
FN
a-SMA
Metrnl
Tubulin
250KDa
42KDa
34KDa
55KDa
250KDa
40KDa
35KDa
55KDa

Fig.1

H

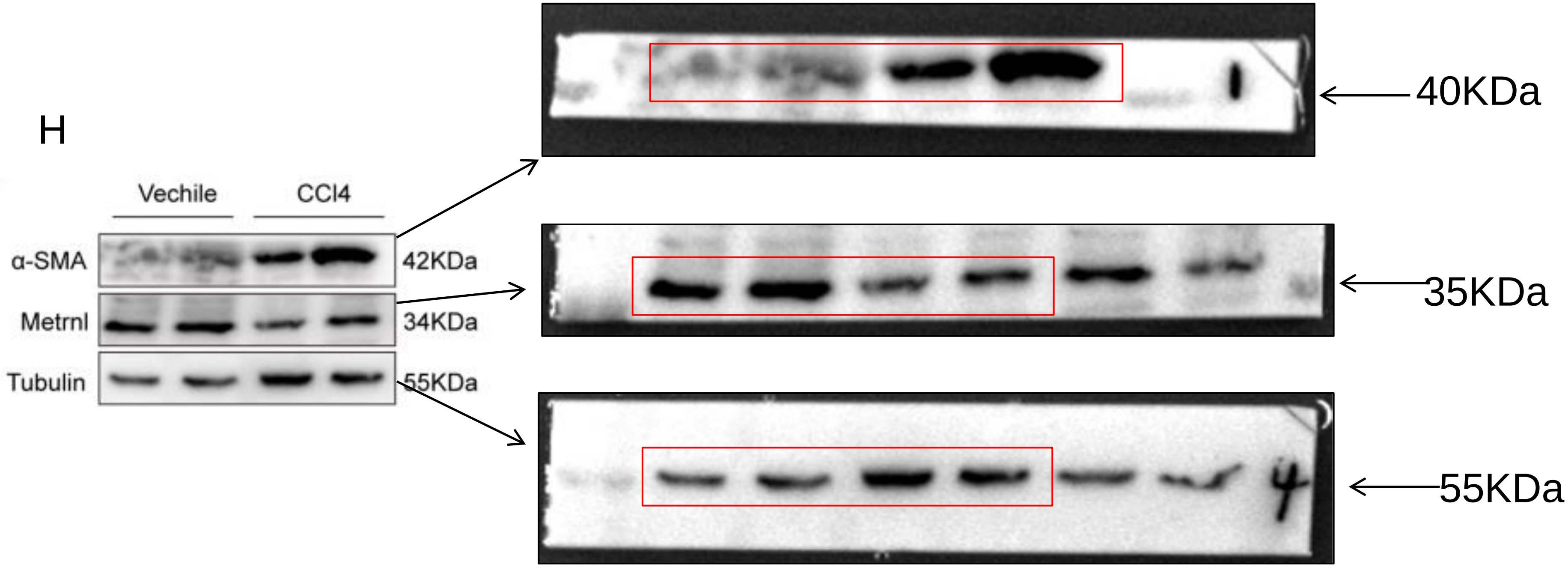

Vechile
CCl4
α-SMA
42KDa
Metrnl
34KDa
Tubulin
55KDa
40KDa
35KDa
55KDa

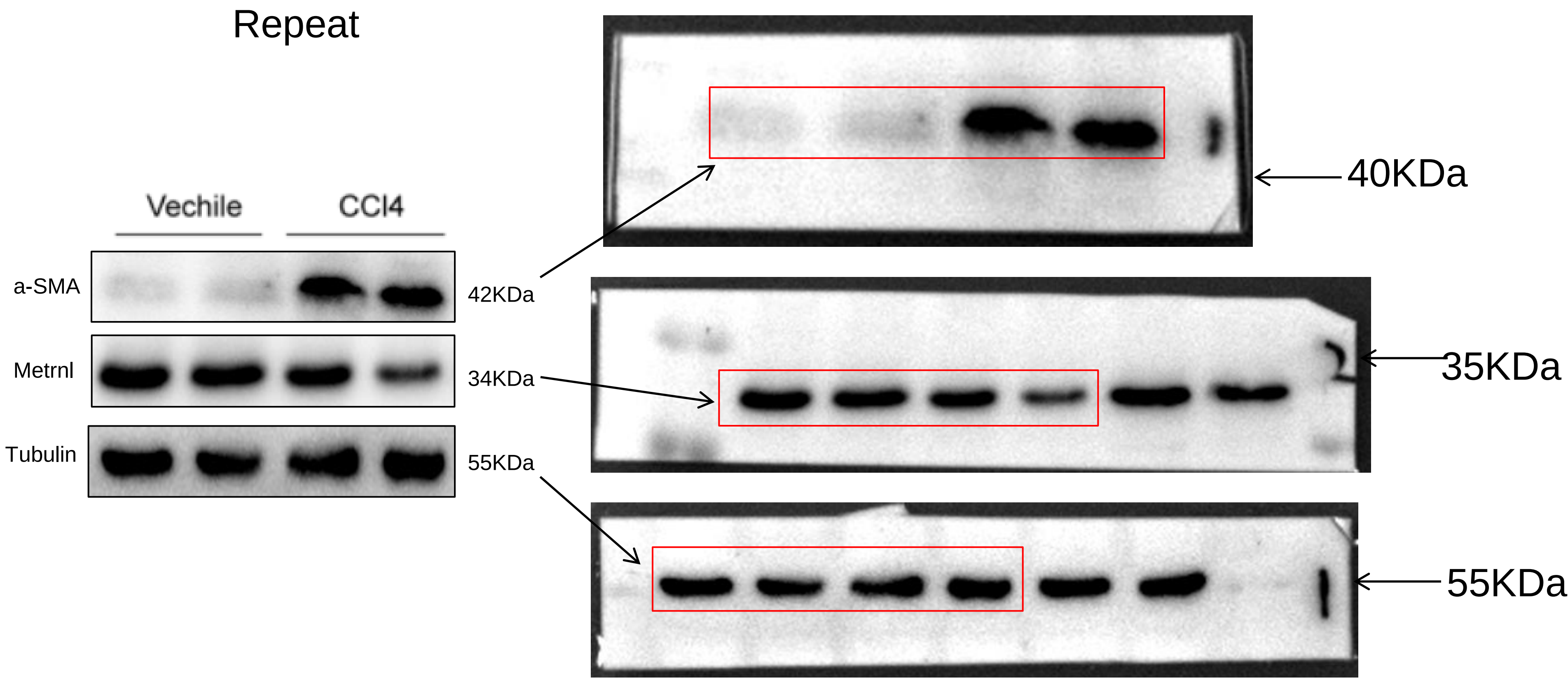
Repeat
Vechile
CCl4
a-SMA
Metrnl
Tubulin
42KDa
34KDa
55KDa
40KDa
35KDa
55KDa

Repeat

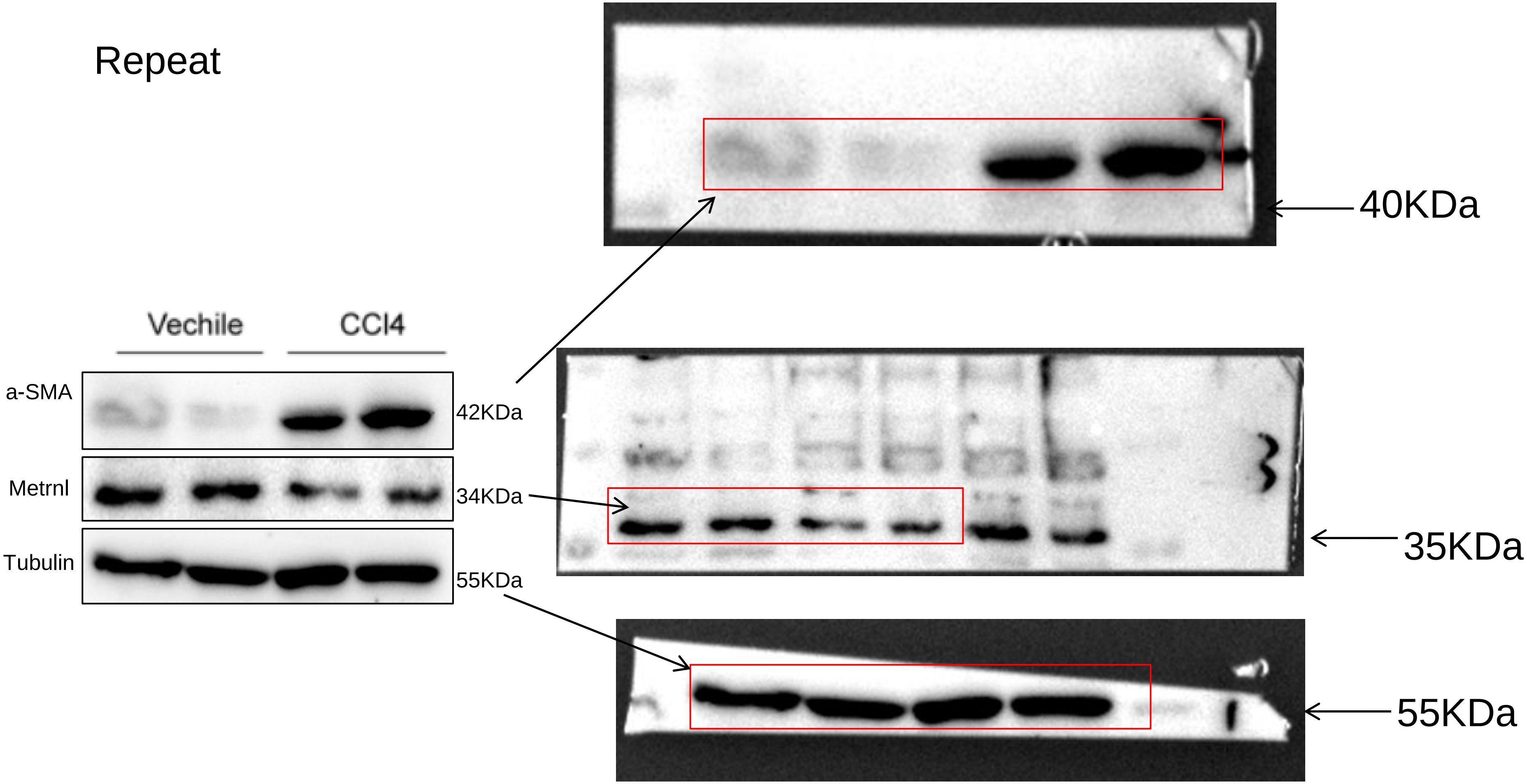
Vechile
CCl4
a-SMA
Metrnl
Tubulin
42KDa
34KDa
55KDa
40KDa
35KDa
55KDa

Fig.2

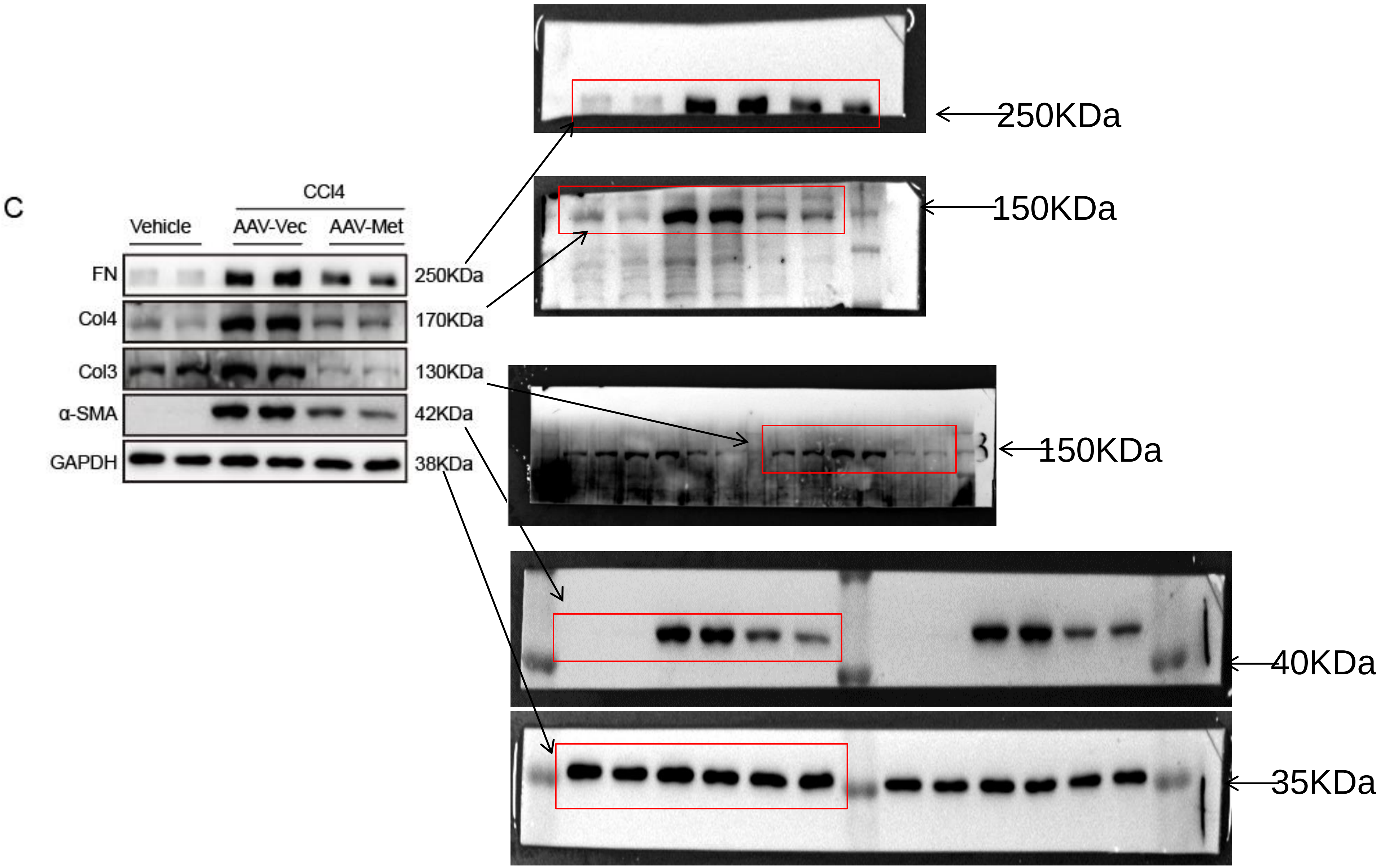
C
CCl4
Vehicle
AAV-Vec
AAV-Met
FN
Col4
Col3
α-SMA
GAPDH
250KDa
170KDa
130KDa
42KDa
38KDa
250KDa
150KDa
150KDa
40KDa
35KDa

Repeat

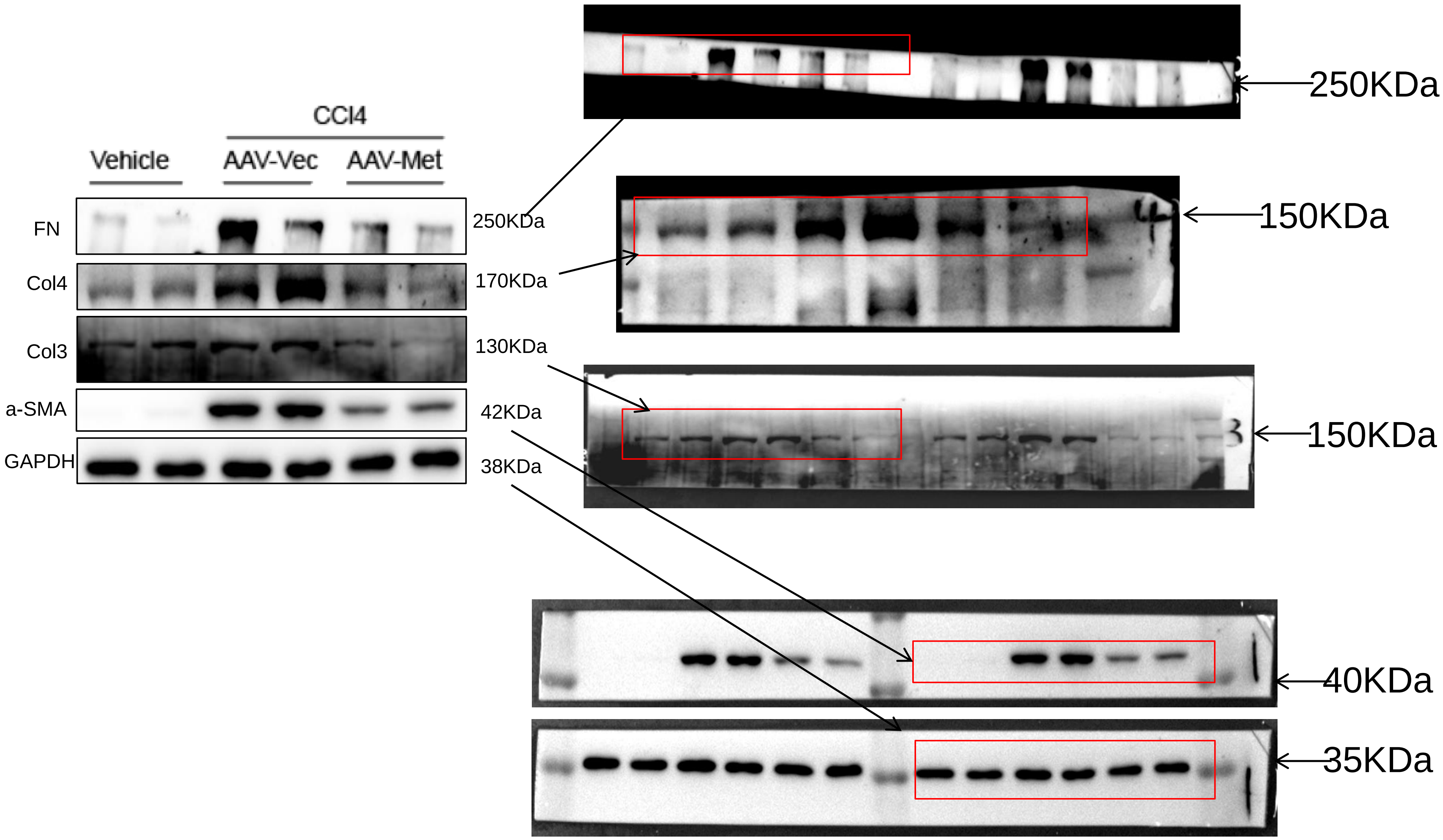

CCl4
Vehicle
AAV-Vec
AAV-Met
FN
Col4
Col3
a-SMA
GAPDH
250KDa
170KDa
130KDa
42KDa
38KDa
250KDa
150KDa
150KDa
40KDa
35KDa

Repeat

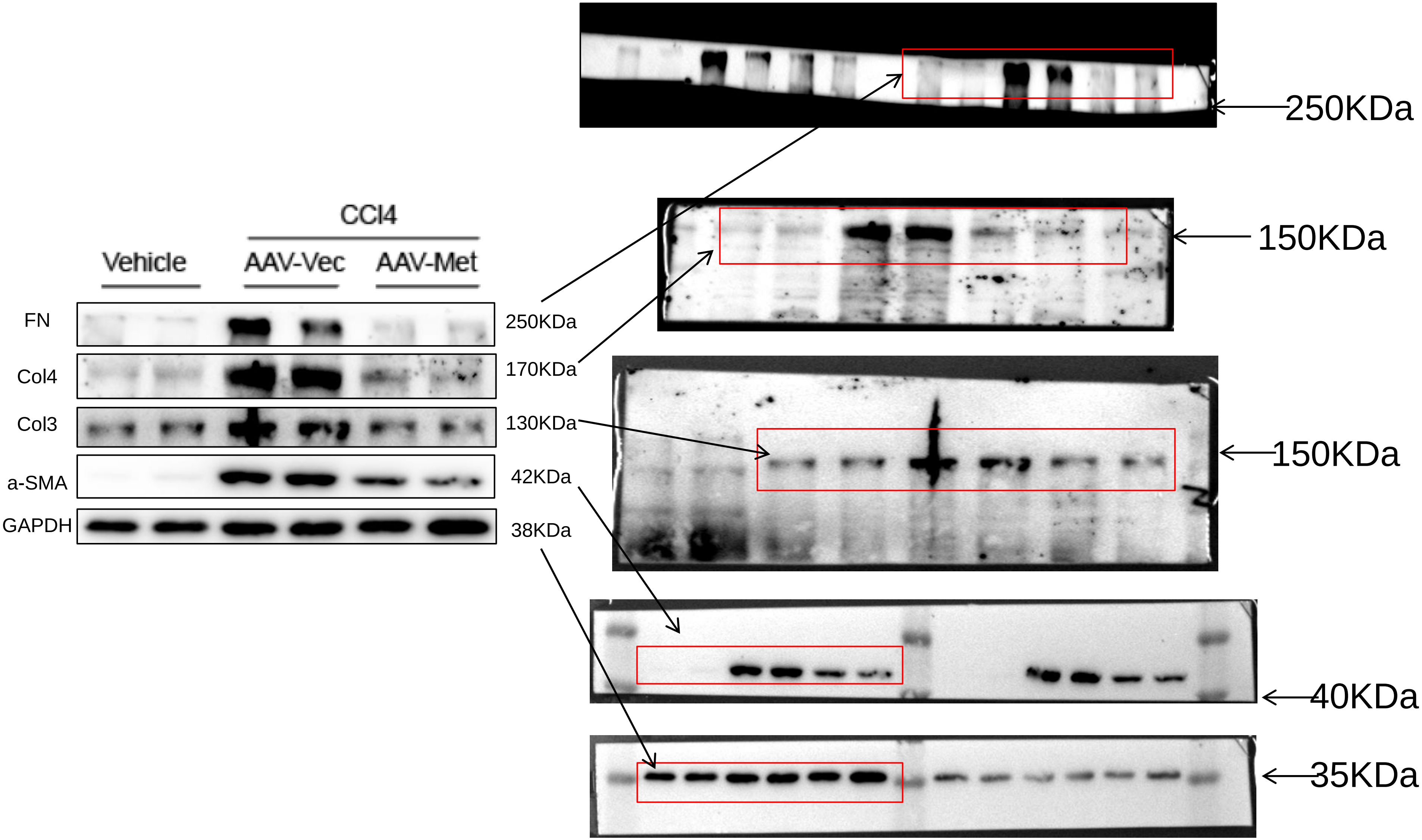
CCl4
Vehicle
AAV-Vec
AAV-Met
FN
Col4
Col3
a-SMA
GAPDH
250KDa
170KDa
130KDa
42KDa
38KDa
250KDa
150KDa
150KDa
40KDa
35KDa

Fig.3

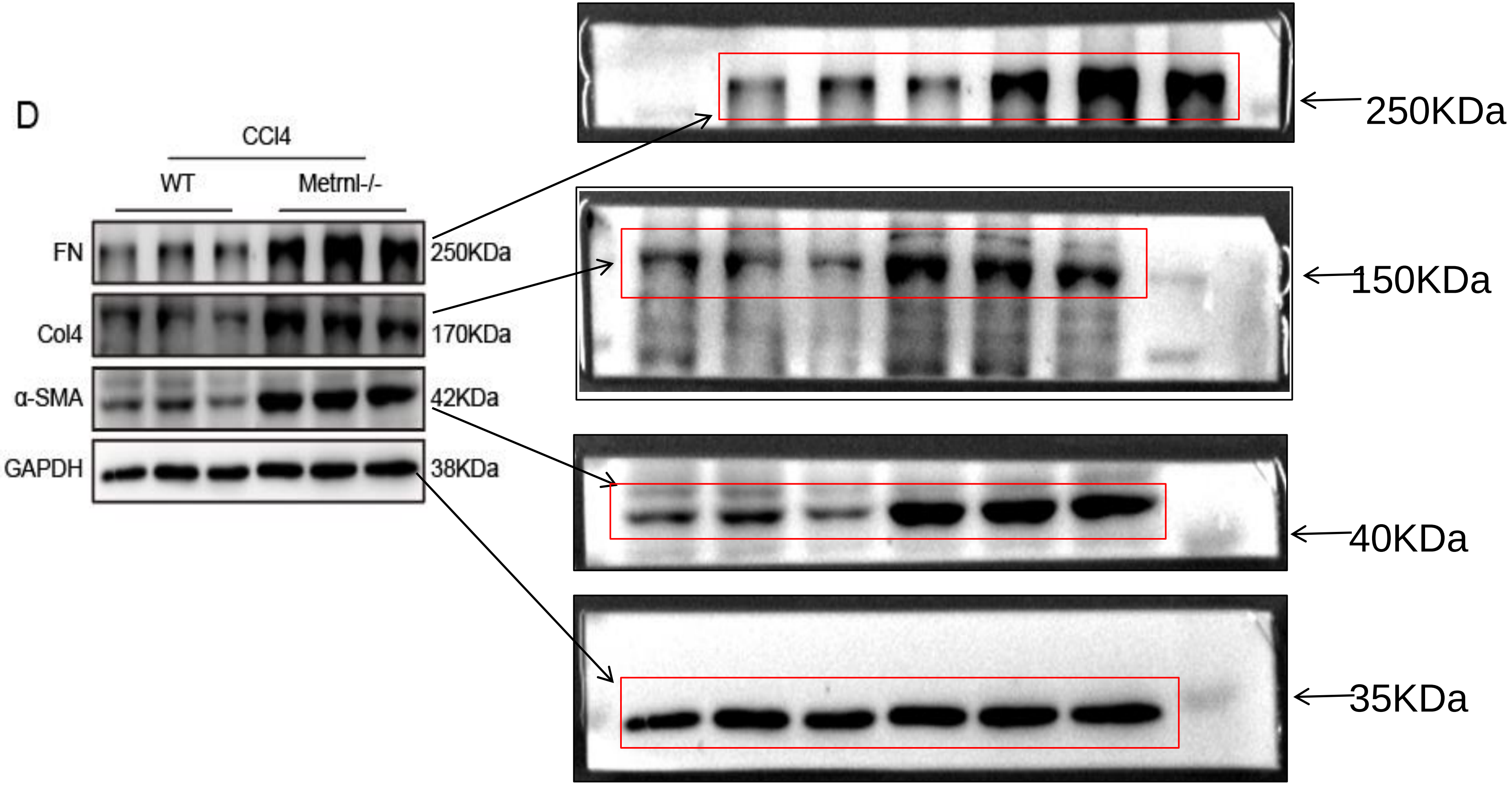
D
CCl4
WT
Metrnl-/-
FN
Col4
α-SMA
GAPDH
250KDa
170KDa
42KDa
38KDa
250KDa
150KDa
40KDa
35KDa

Repeat

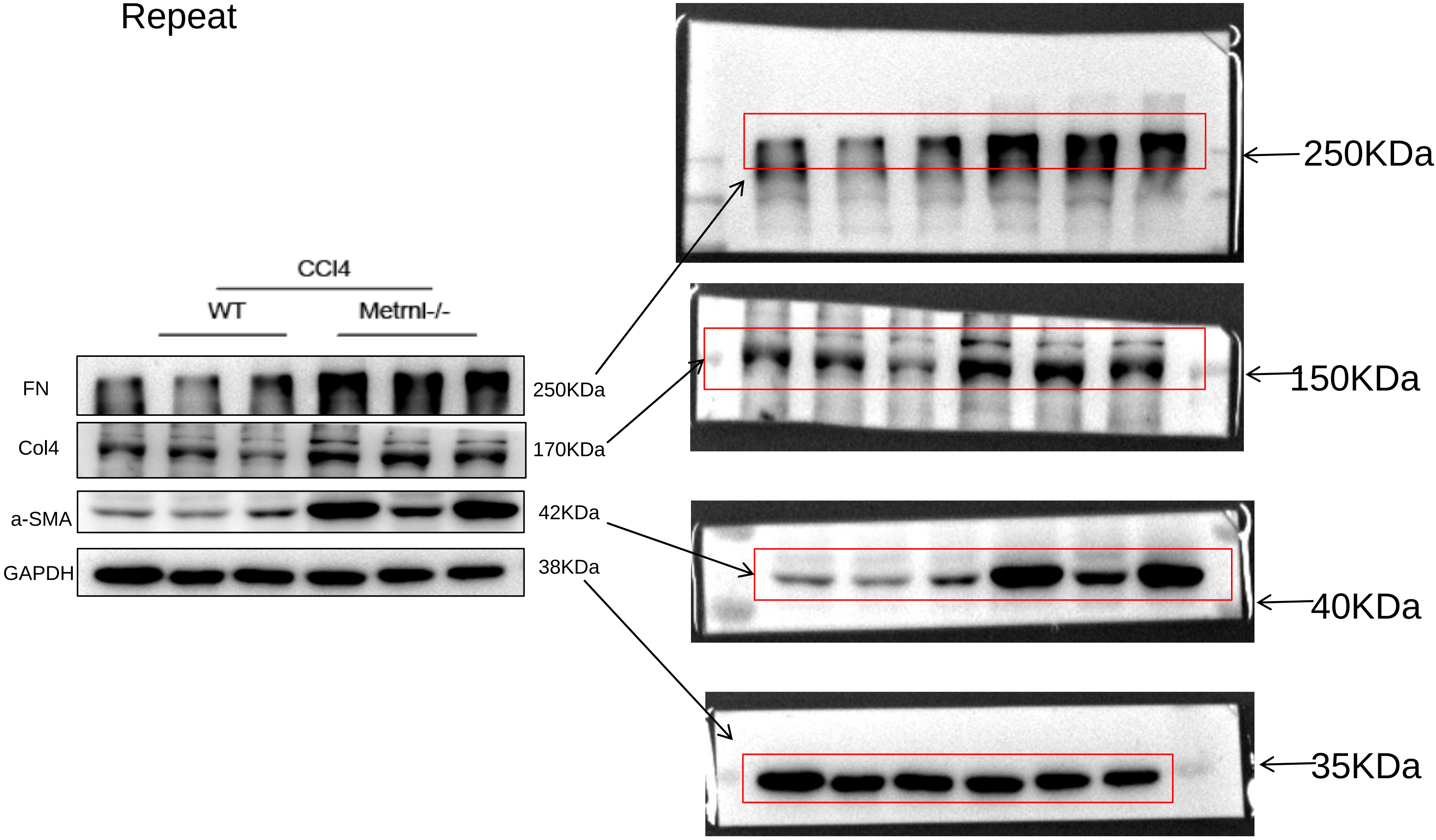
CCl4
WT
Metrnl-/-
FN
Col4
a-SMA
GAPDH
250KDa
170KDa
42KDa
38KDa
250KDa
150KDa
40KDa
35KDa

Repeat

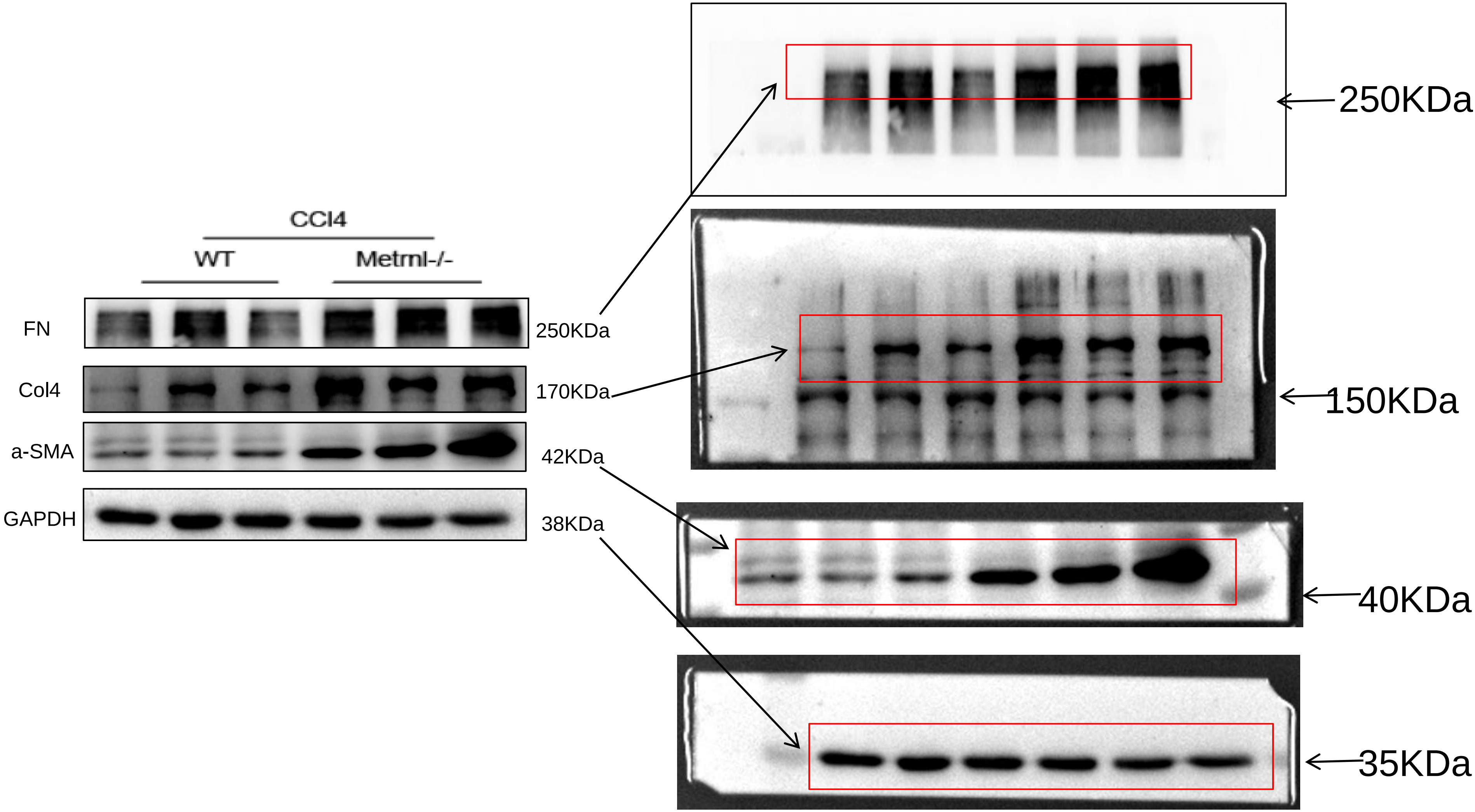
CCl4
WT
Metrnl-/-
FN
Col4
a-SMA
GAPDH
250KDa
170KDa
42KDa
38KDa
250KDa
150KDa
40KDa
35KDa

Fig.4

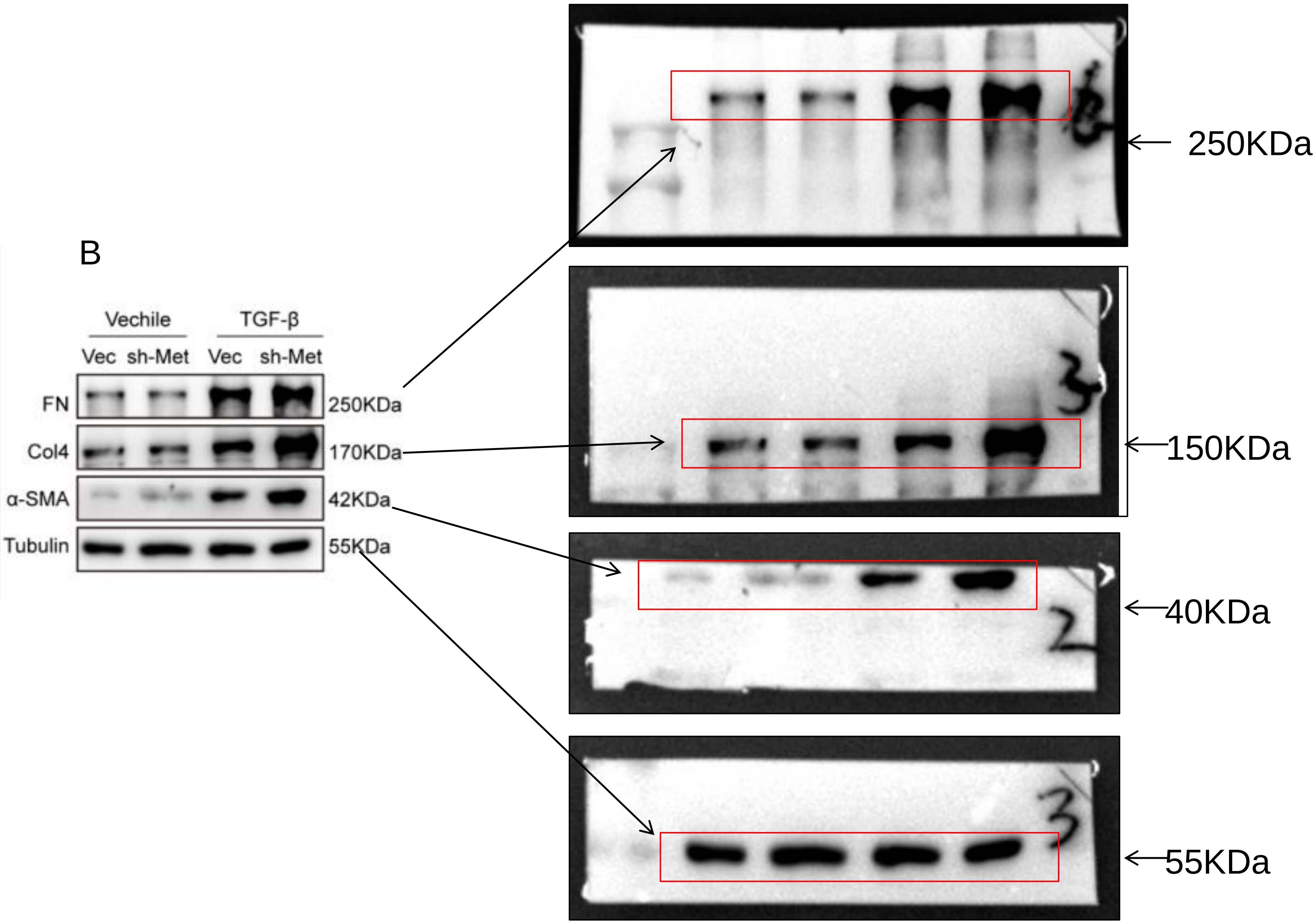

B
Vechile
TGF-β
Vec
sh-Met
Vec
sh-Met
FN
Col4
α-SMA
Tubulin
250KDa
170KDa
42KDa
55KDa
250KDa
150KDa
40KDa
55KDa

Repeat

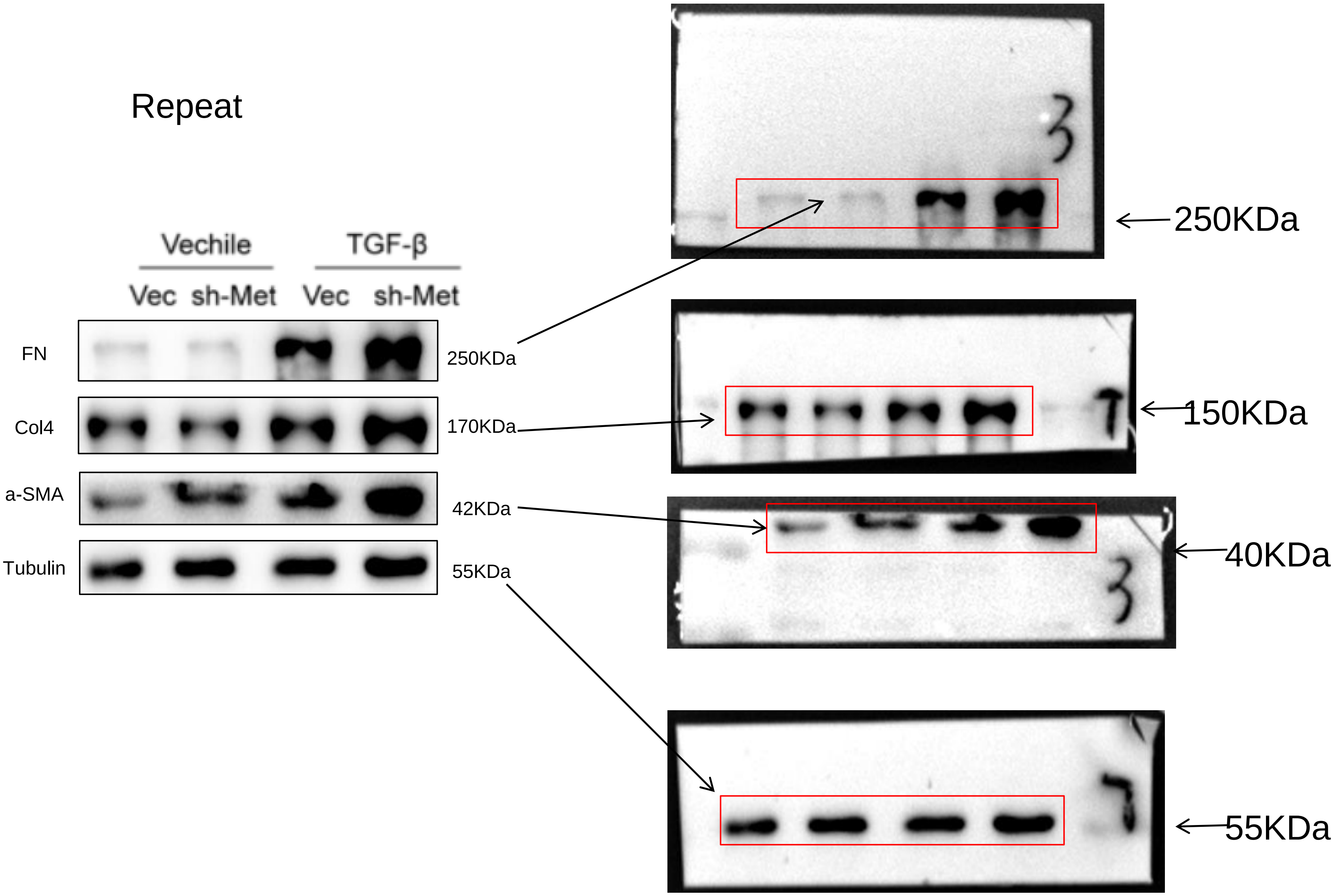
Vechile
TGF-β
Vec
sh-Met
Vec
sh-Met
FN
Col4
a-SMA
Tubulin
250KDa
170KDa
42KDa
55KDa
250KDa
150KDa
40KDa
55KDa

Repeat

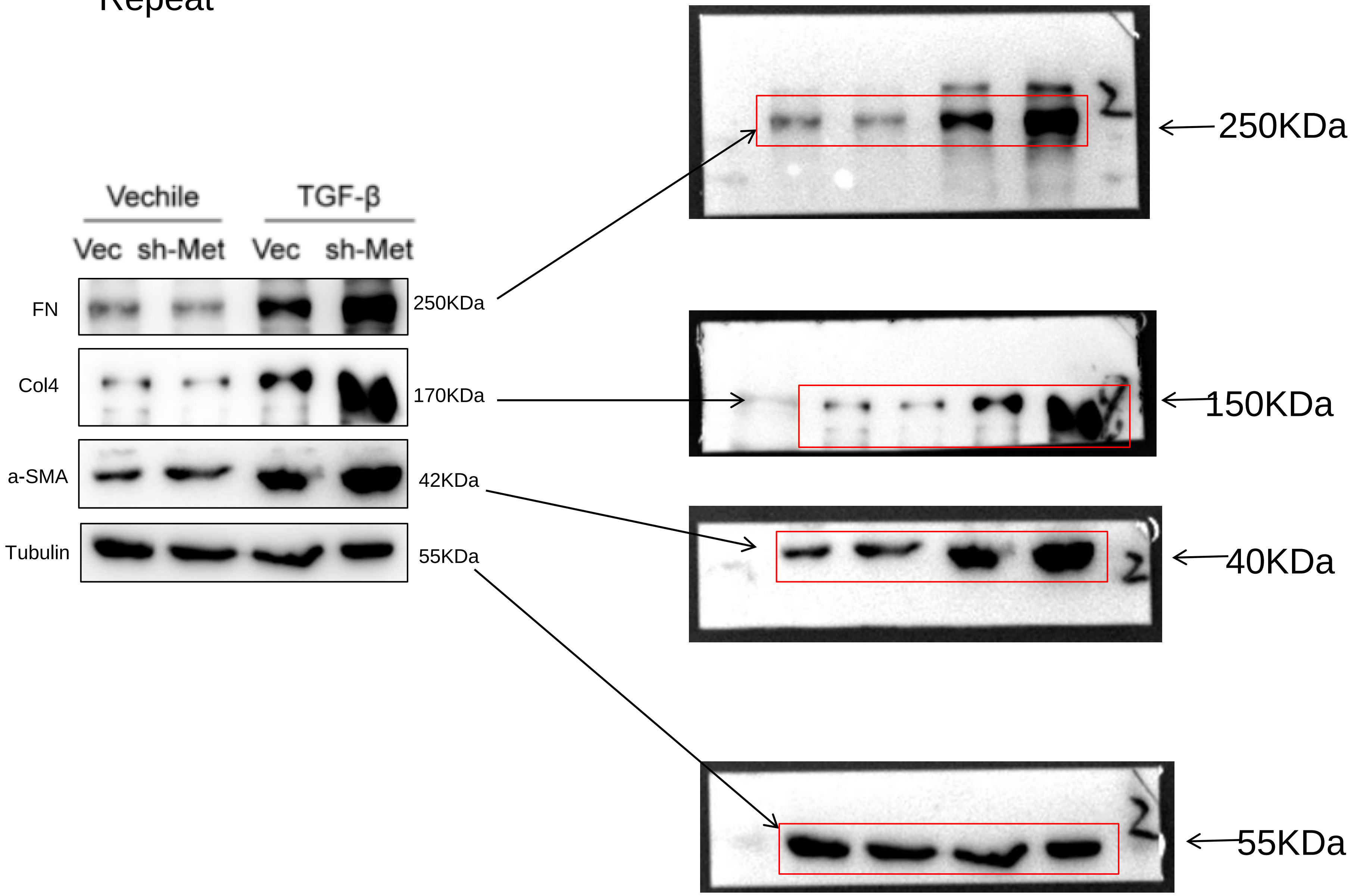

Vechile
TGF-β
Vec sh-Met Vec sh-Met
FN
Col4
a-SMA
Tubulin
250KDa
170KDa
42KDa
55KDa
250KDa
150KDa
40KDa
55KDa

Fig.4

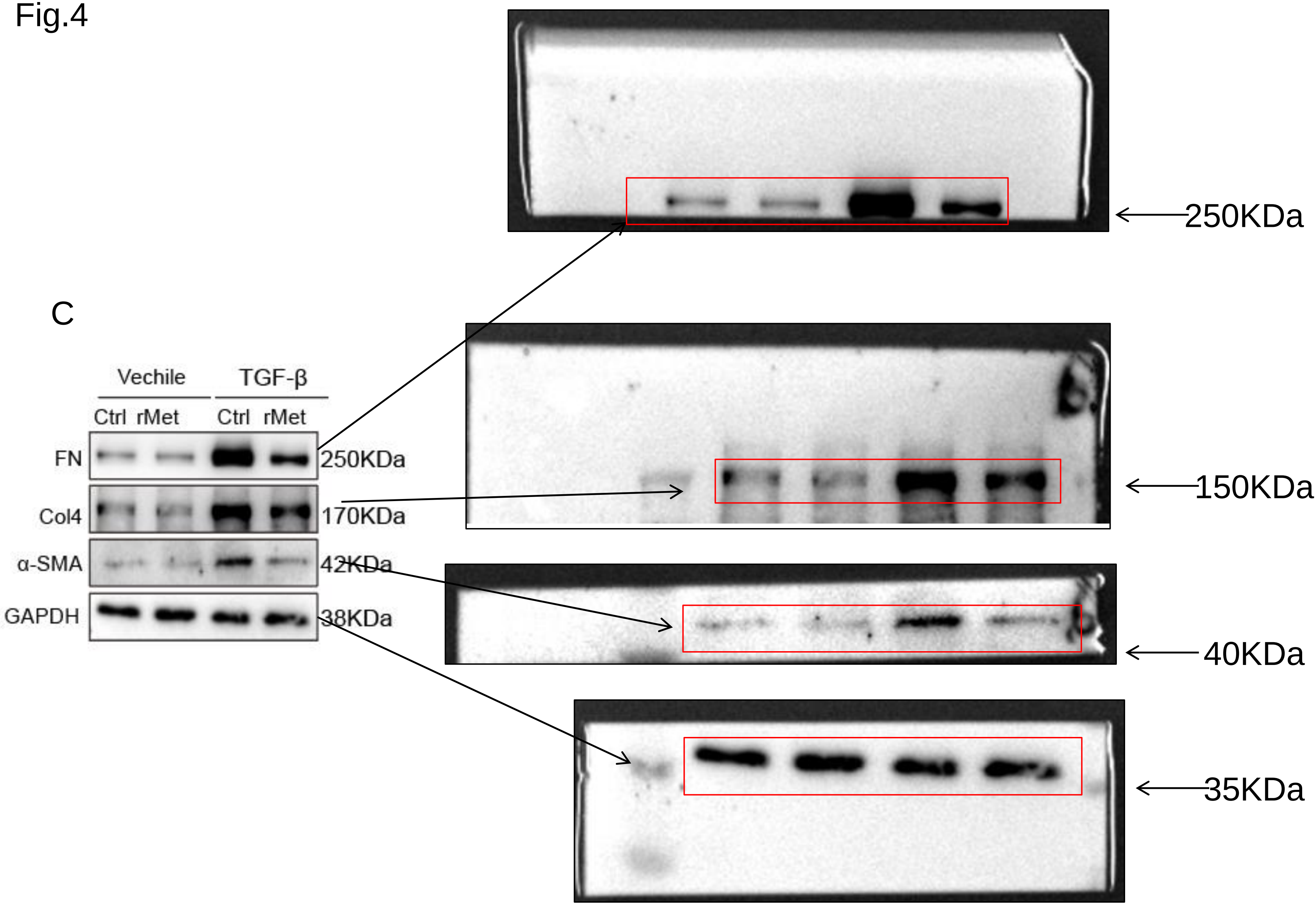

C
Vechile
TGF-β
Ctrl
rMet
Ctrl
rMet
FN
Col4
α-SMA
GAPDH
250KDa
170KDa
42KDa
38KDa
250KDa
150KDa
40KDa
35KDa

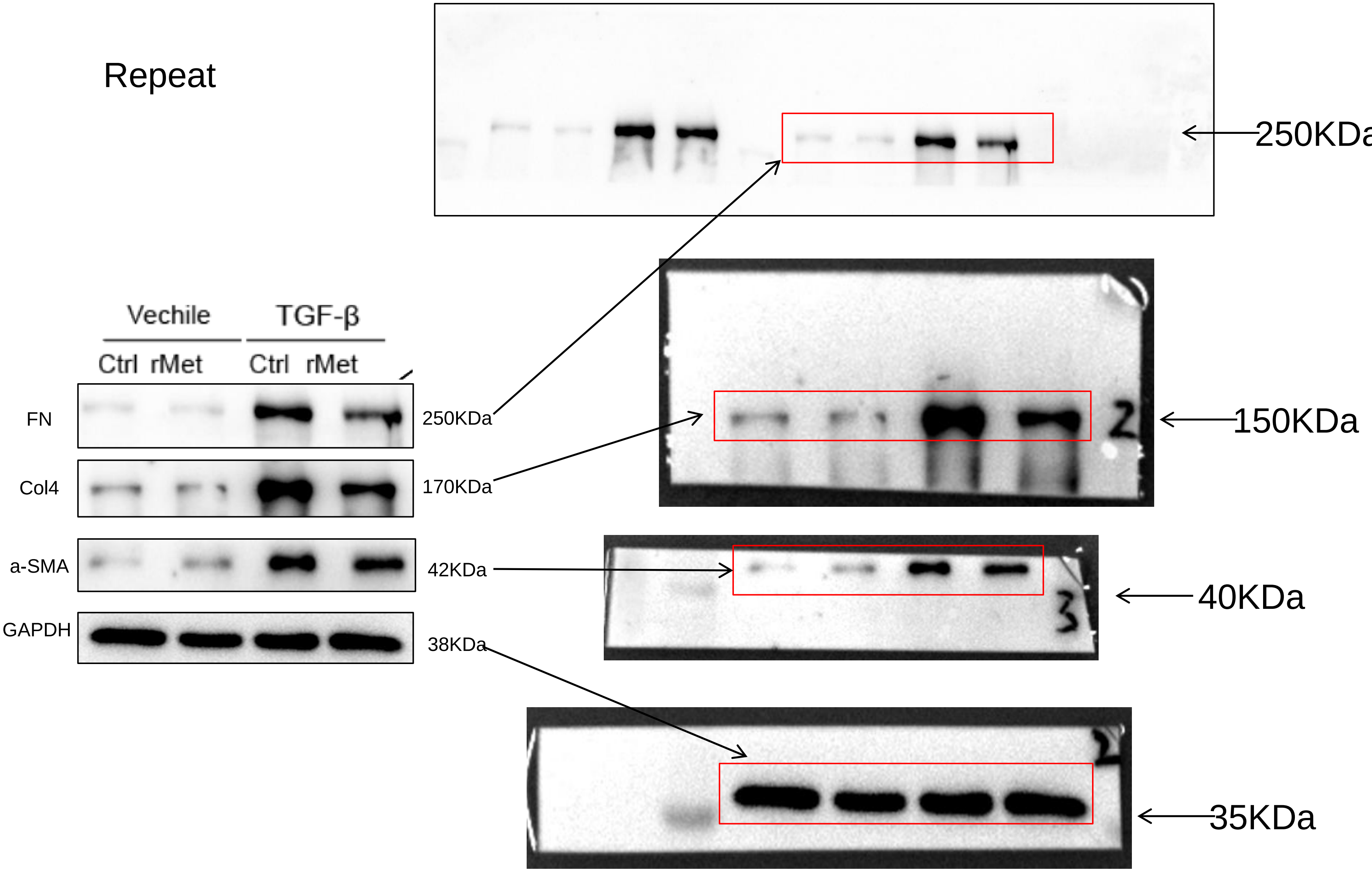
Repeat
Vechile
TGF-β
Ctrl rMet
Ctrl rMet
FN
Col4
a-SMA
GAPDH
250KDa
170KDa
42KDa
38KDa
250KDa
150KDa
40KDa
35KDa

Repeat

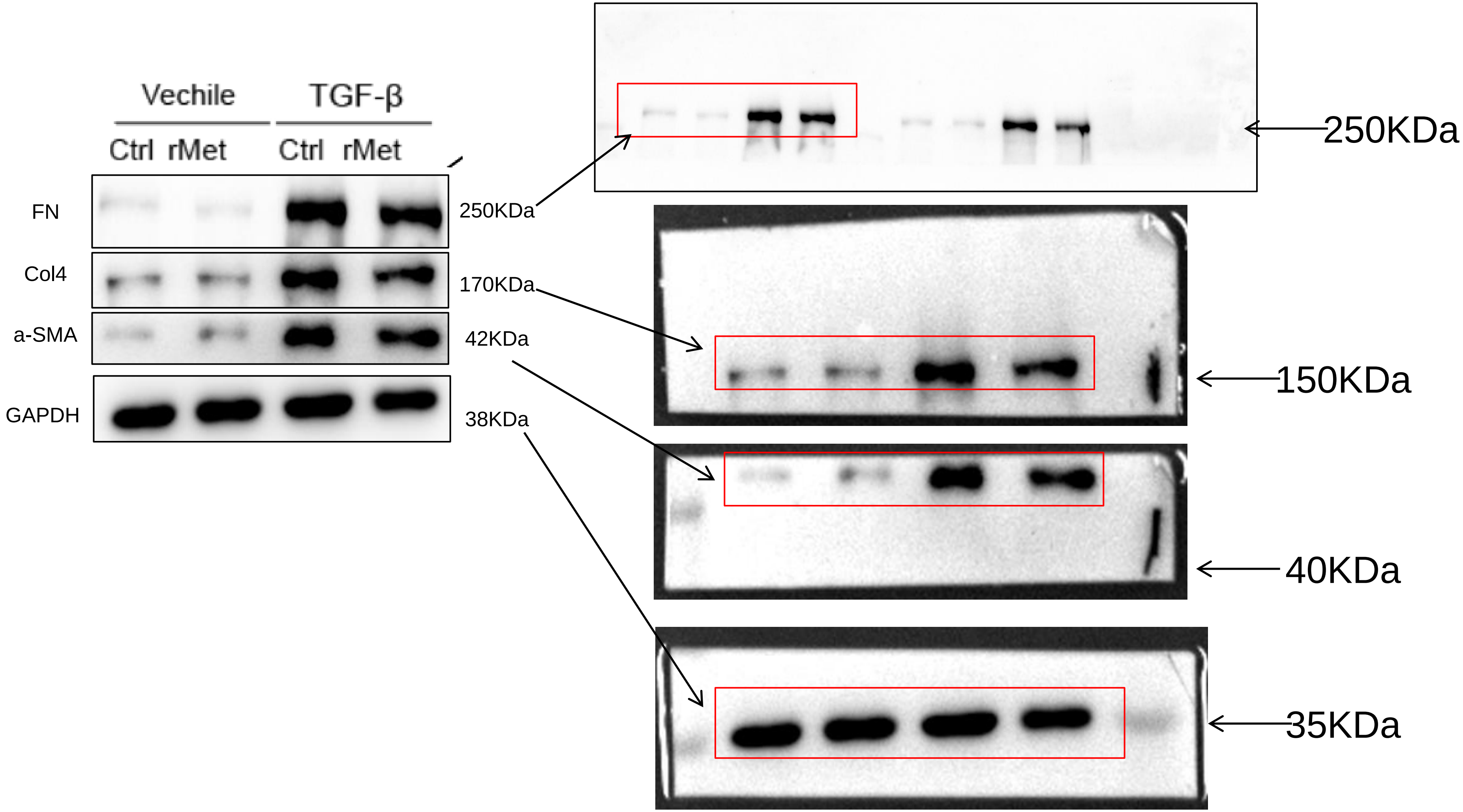

Vechile
TGF-β
Ctrl rMet Ctrl rMet
FN
Col4
a-SMA
GAPDH
250KDa
170KDa
42KDa
38KDa
250KDa
150KDa
40KDa
35KDa

Fig.4

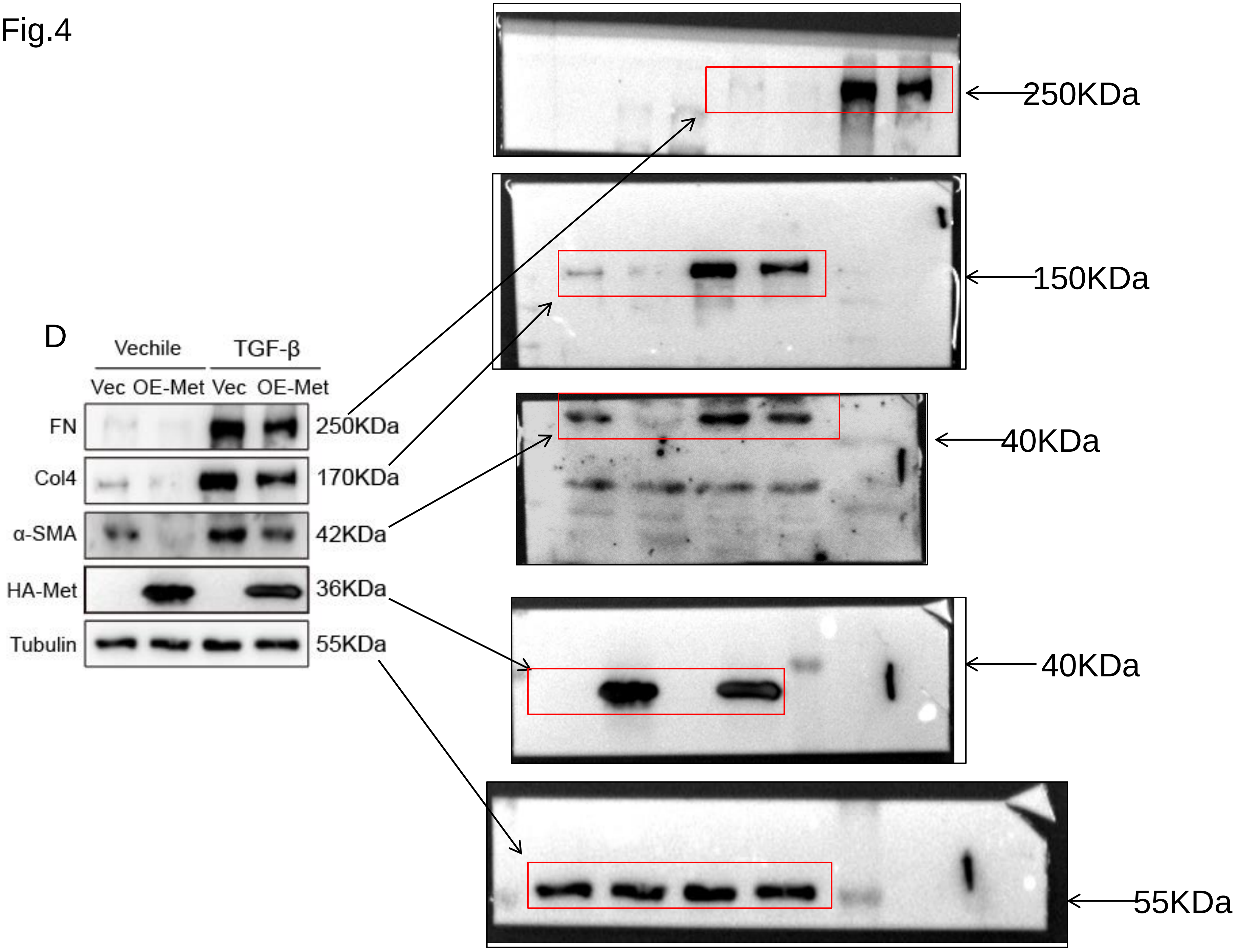
D
Vechile
TGF-β
Vec OE-Met Vec OE-Met
FN
Col4
α-SMA
HA-Met
Tubulin
250KDa
170KDa
42KDa
36KDa
55KDa
250KDa
150KDa
40KDa
40KDa
55KDa

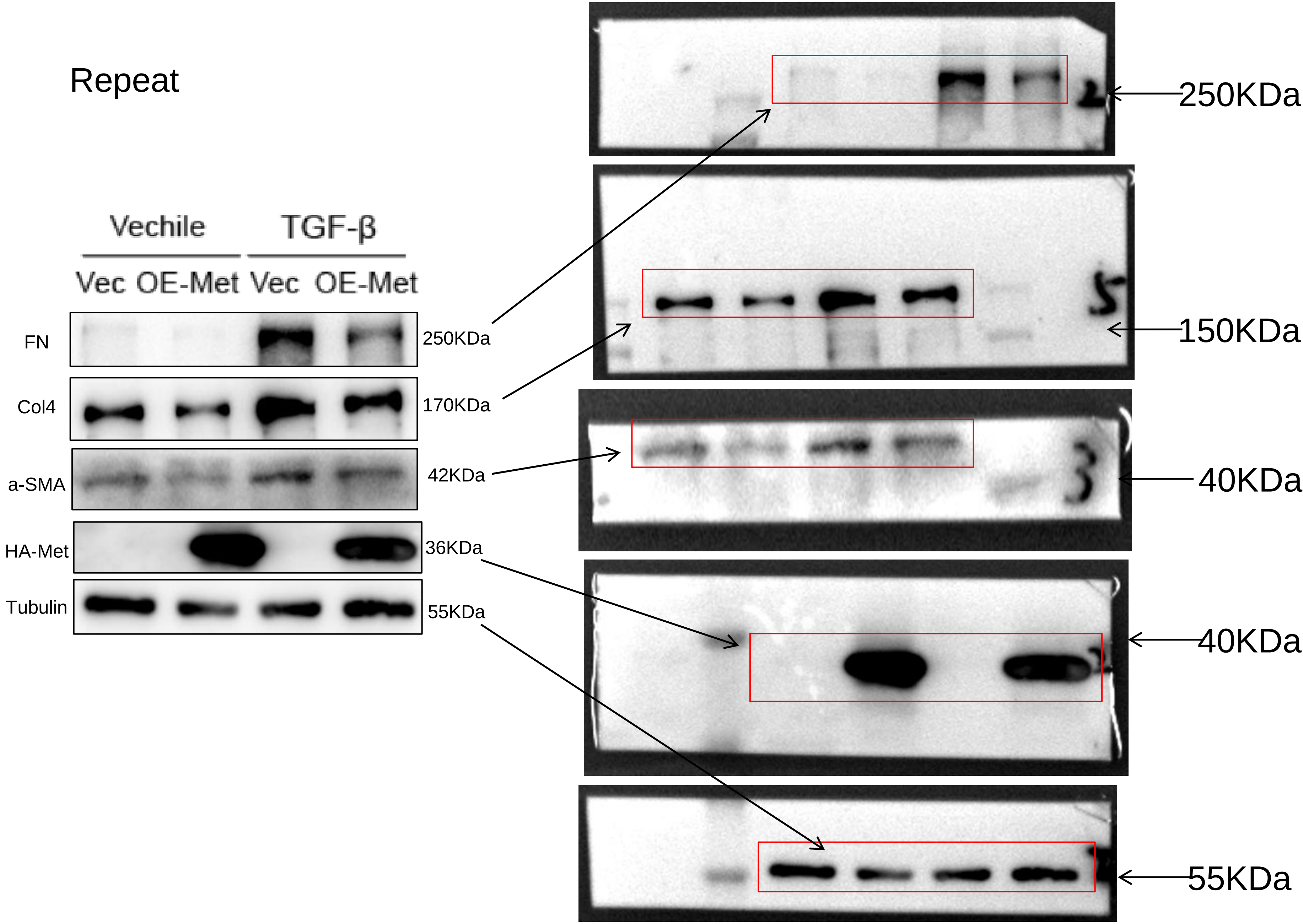

Repeat
Vechile
TGF-β
Vec OE-Met Vec OE-Met
FN
Col4
a-SMA
HA-Met
Tubulin
250KDa
170KDa
42KDa
36KDa
55KDa
250KDa
150KDa
40KDa
40KDa
55KDa

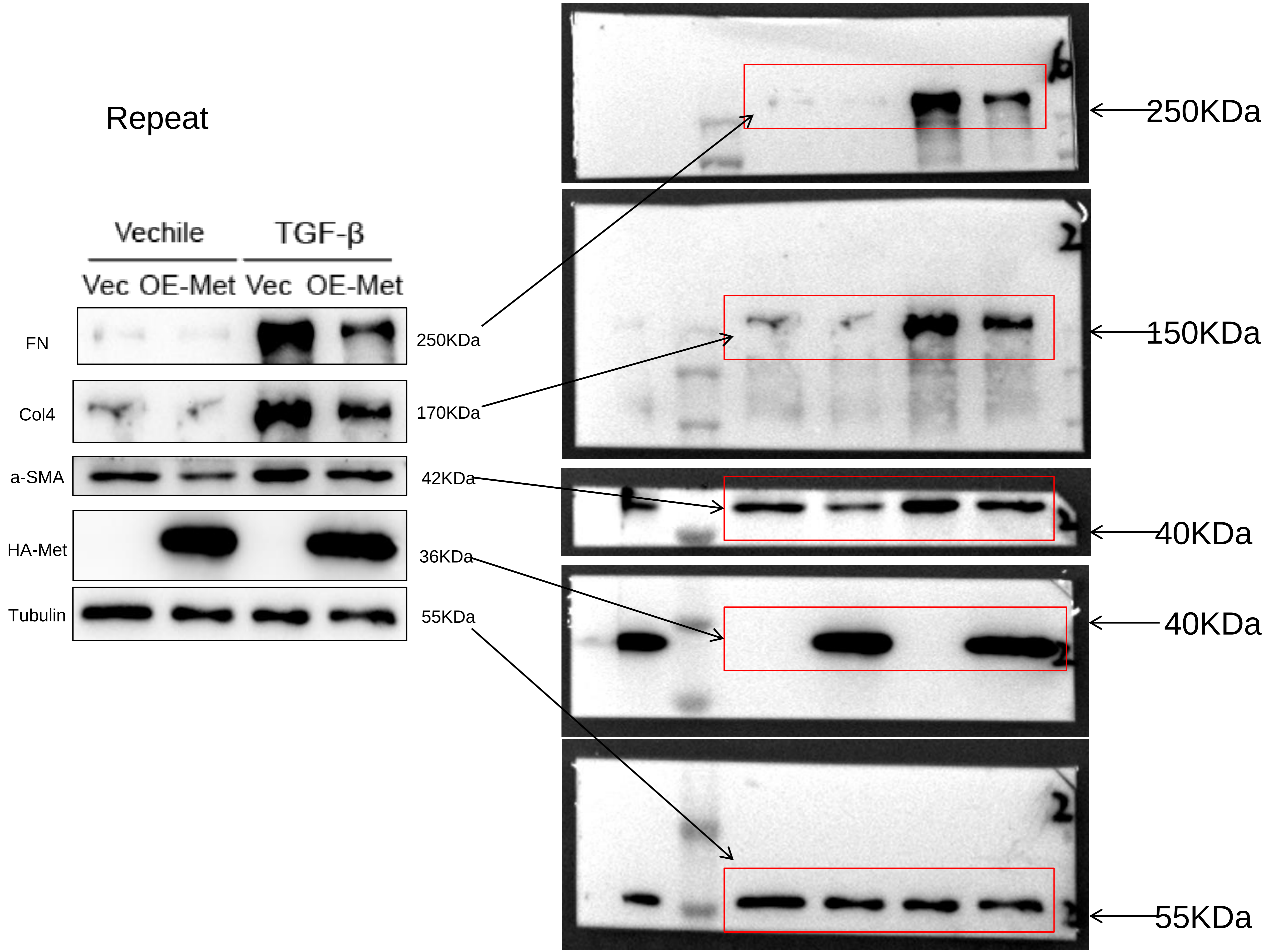
Repeat
Vechile
TGF-β
Vec
OE-Met
Vec
OE-Met
FN
Col4
a-SMA
HA-Met
Tubulin
250KDa
170KDa
42KDa
36KDa
55KDa
250KDa
150KDa
40KDa
40KDa
55KDa

Fig.5

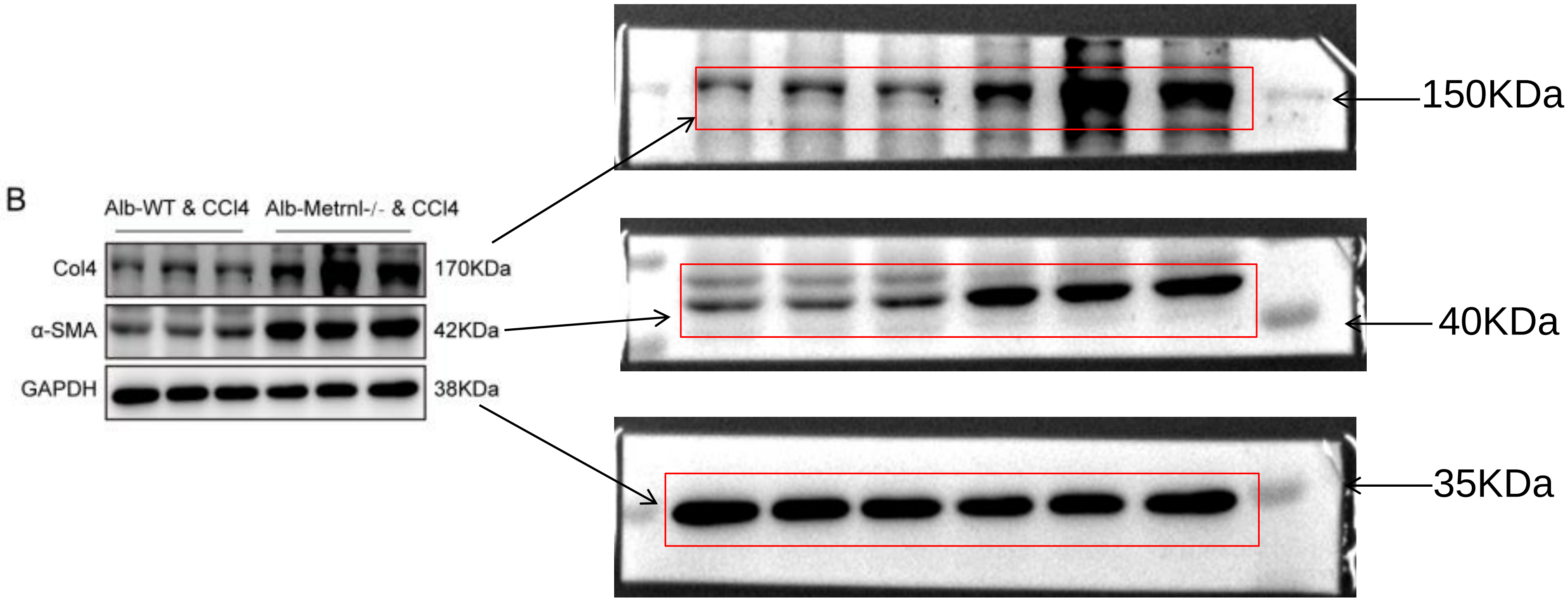
B
Alb-WT & CCl4
Alb-Metrnl-/- & CCl4
Col4
170KDa
α-SMA
42KDa
GAPDH
38KDa
150KDa
40KDa
35KDa

Repeat

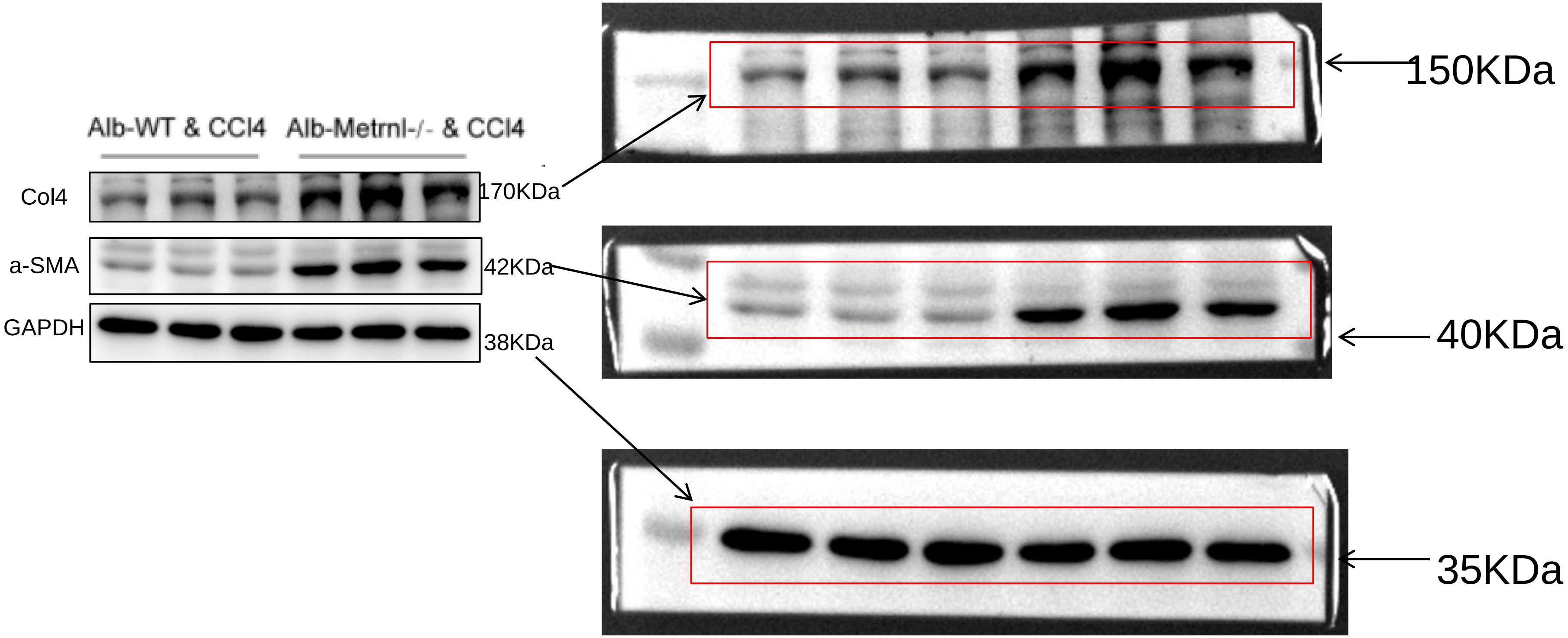

Alb-WT & CCl4
Alb-Metrnl-/- & CCl4
Col4
a-SMA
GAPDH
170KDa
42KDa
38KDa
150KDa
40KDa
35KDa

Repeat

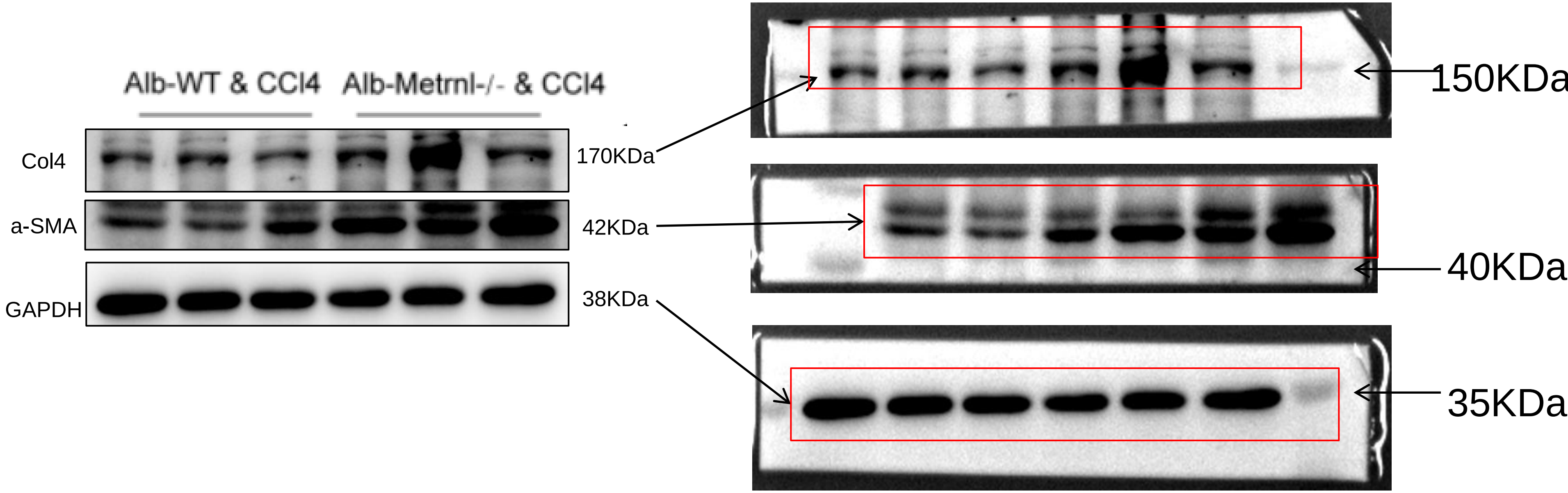
Alb-WT & CCl4
Alb-Metrnl-/- & CCl4
Col4
a-SMA
GAPDH
170KDa
42KDa
38KDa
150KDa
40KDa
35KDa

Fig.5

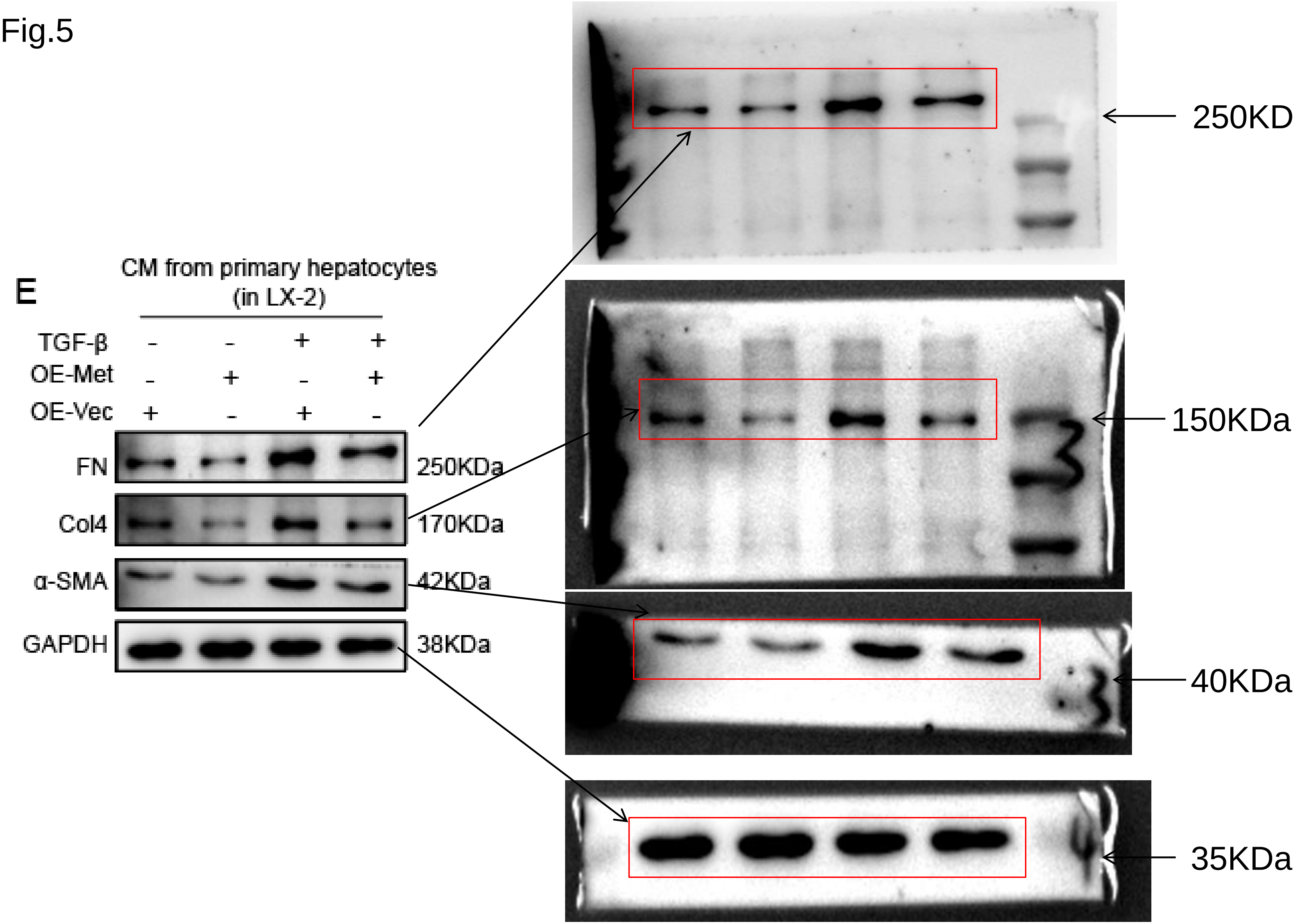
E
CM from primary hepatocytes
(in LX-2)
TGF-β - - + +
OE-Met - + - +
OE-Vec + - + -
FN
Col4
α-SMA
GAPDH
250KDa
170KDa
42KDa
38KDa
250KD
150KDa
40KDa
35KDa

Repeat

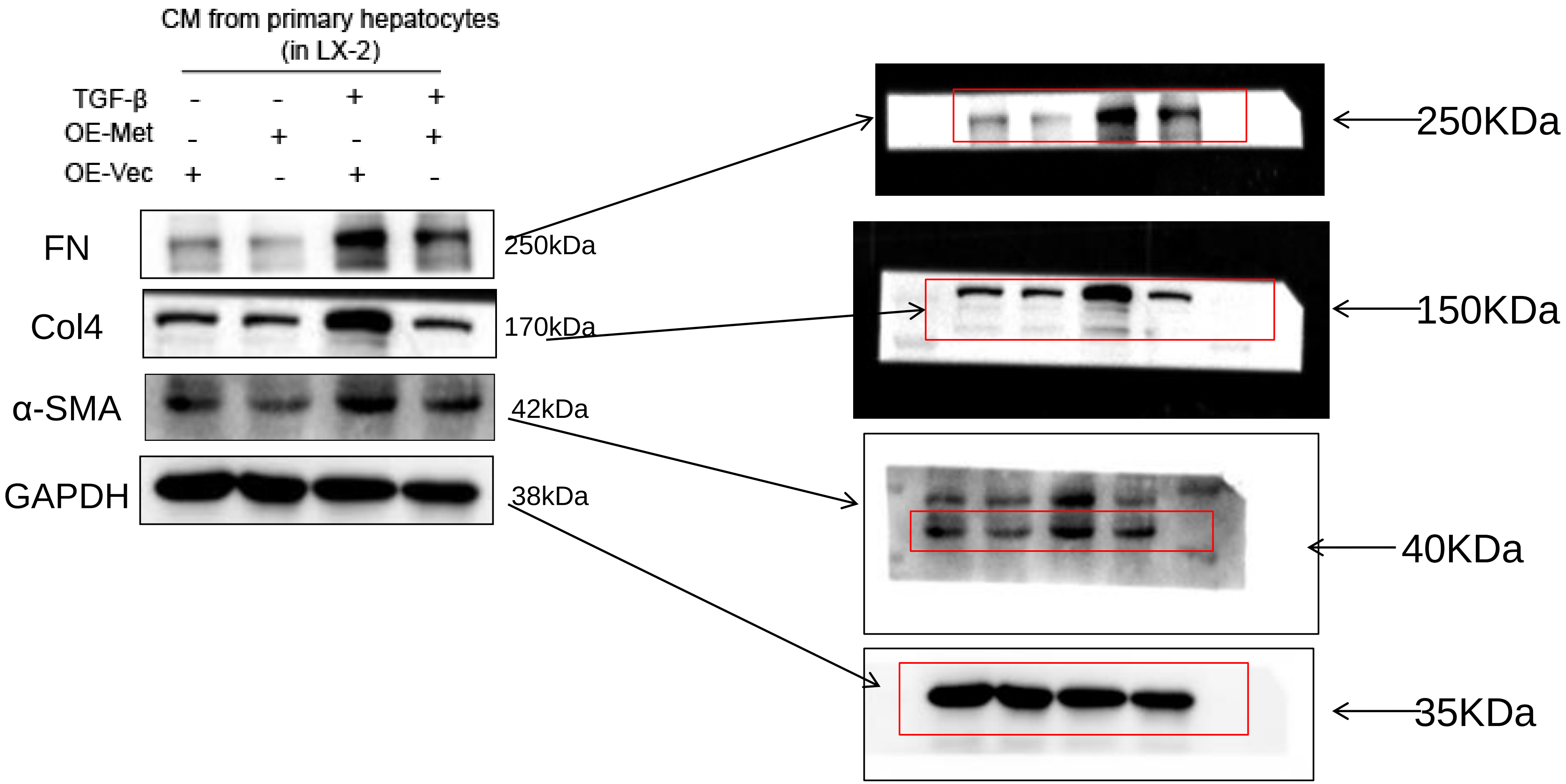

CM from primary hepatocytes
(in LX-2)
TGF-β - - + +
OE-Met - + - +
OE-Vec + - + -
FN
Col4
α-SMA
GAPDH
250kDa
170kDa
42kDa
38kDa
250KDa
150KDa
40KDa
35KDa

Repeat

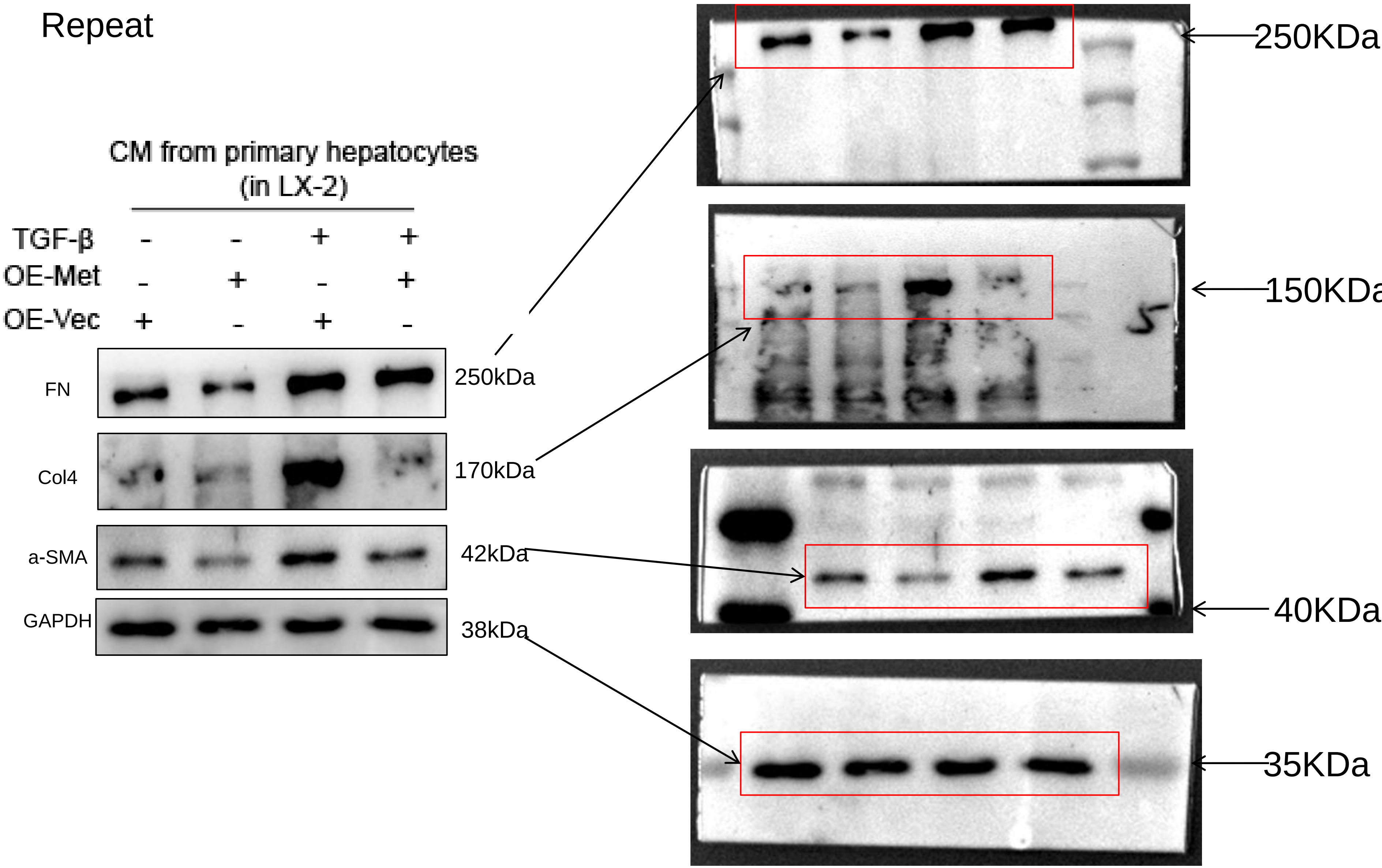
CM from primary hepatocytes
(in LX-2)
TGF-β - - + +
OE-Met - + - +
OE-Vec + - + -
FN
Col4
a-SMA
GAPDH
250kDa
170kDa
42kDa
38kDa
250KDa
150KDa
40KDa
35KDa

Fig.5

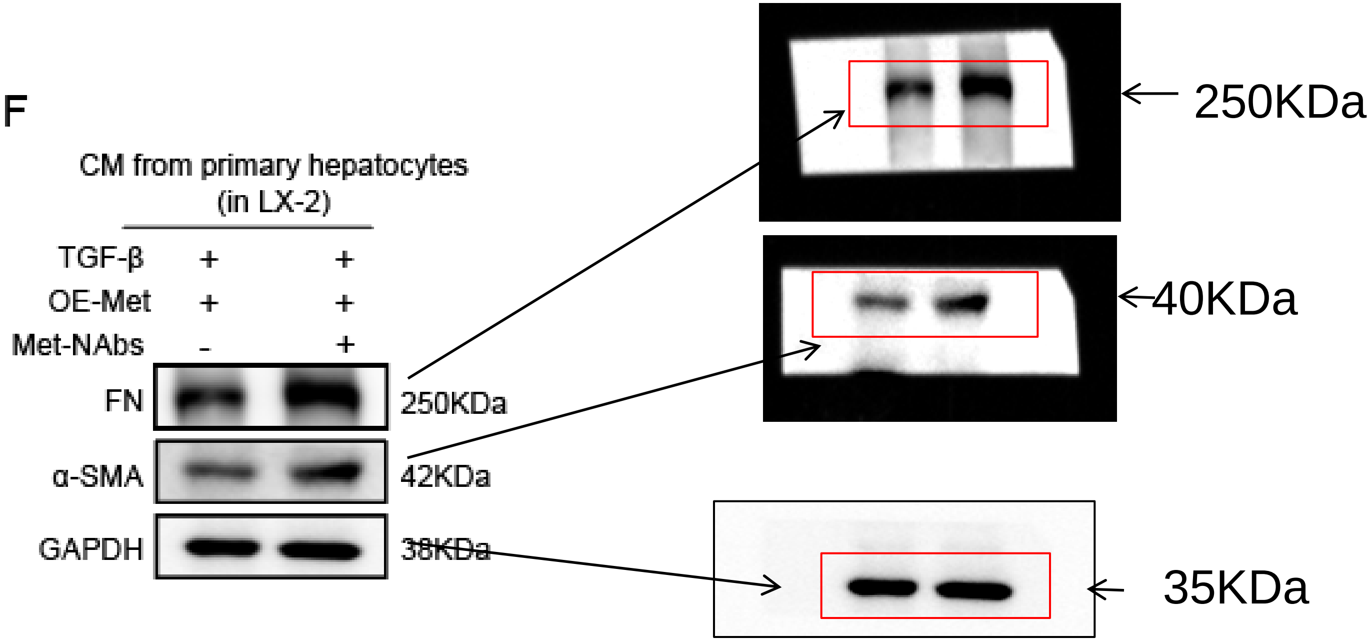
F
CM from primary hepatocytes
(in LX-2)
TGF-β + +
OE-Met + +
Met-NAbs - +
FN
250KDa
α-SMA
42KDa
GAPDH
38KDa
250KDa
40KDa
35KDa

Repeat

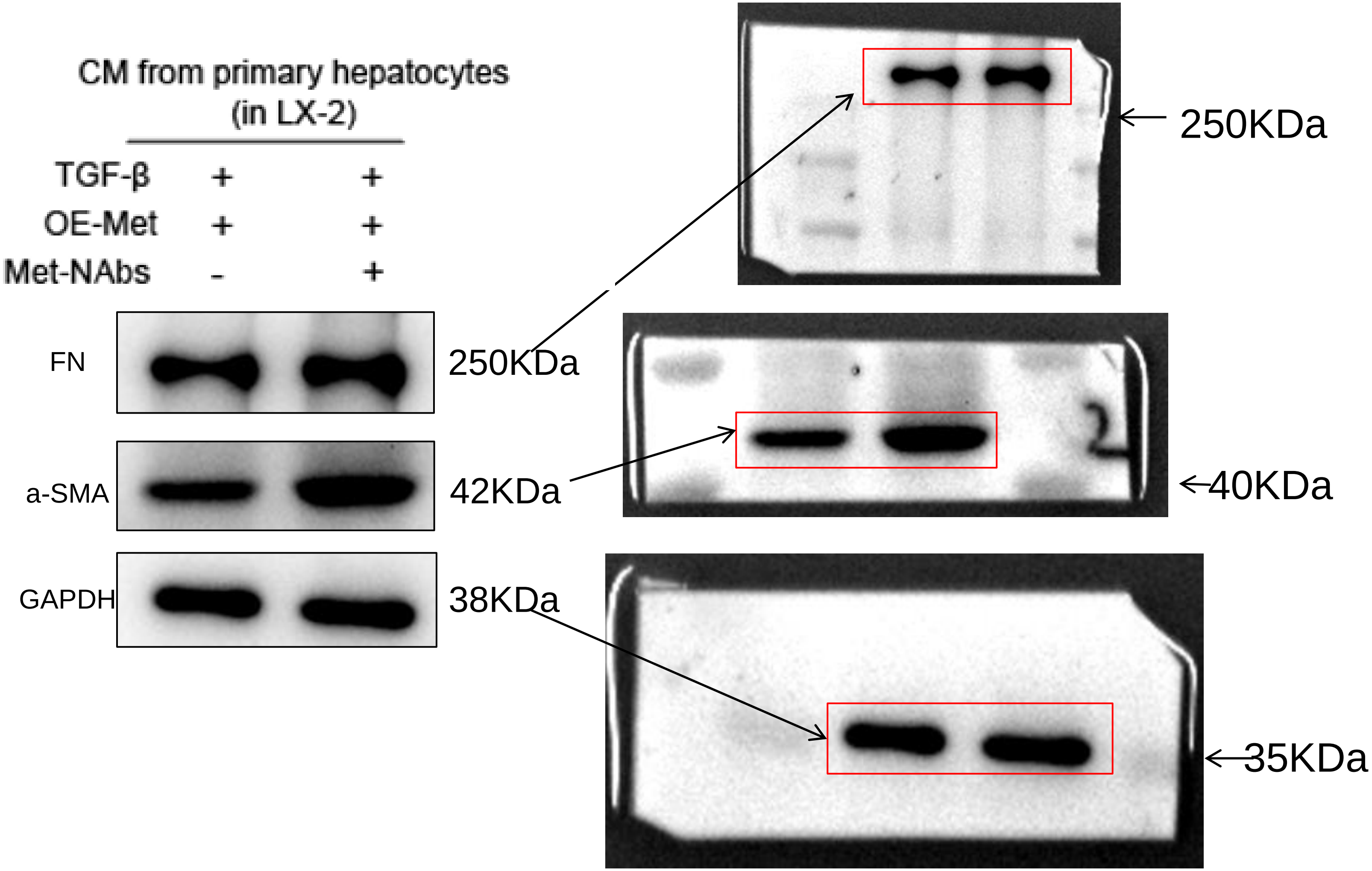

CM from primary hepatocytes
(in LX-2)
TGF-β + +
OE-Met + +
Met-NAbs - +
FN
250KDa
a-SMA
42KDa
GAPDH
38KDa
250KDa
40KDa
35KDa

Repeat

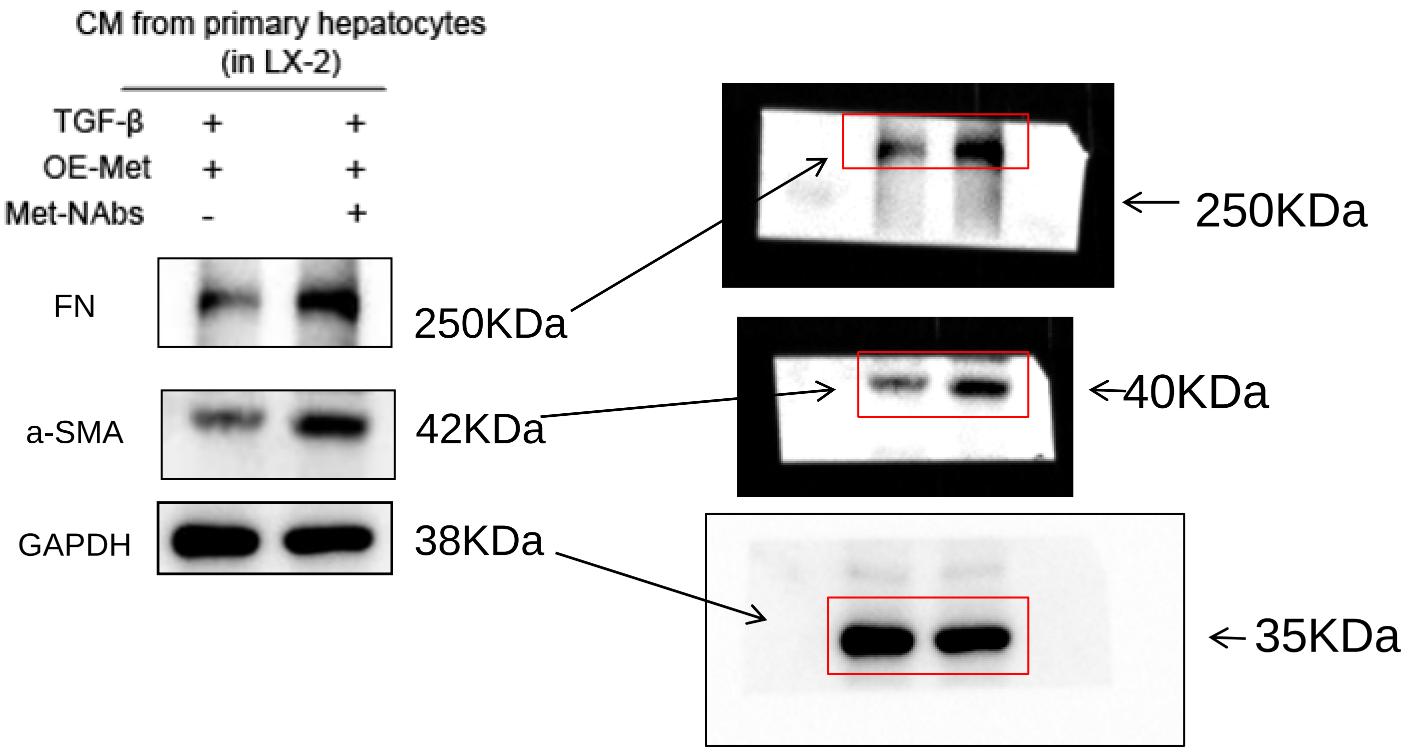
CM from primary hepatocytes
(in LX-2)
TGF-β + +
OE-Met + +
Met-NAbs - +
FN
a-SMA
GAPDH
250KDa
42KDa
38KDa
250KDa
40KDa
35KDa

Fig.5

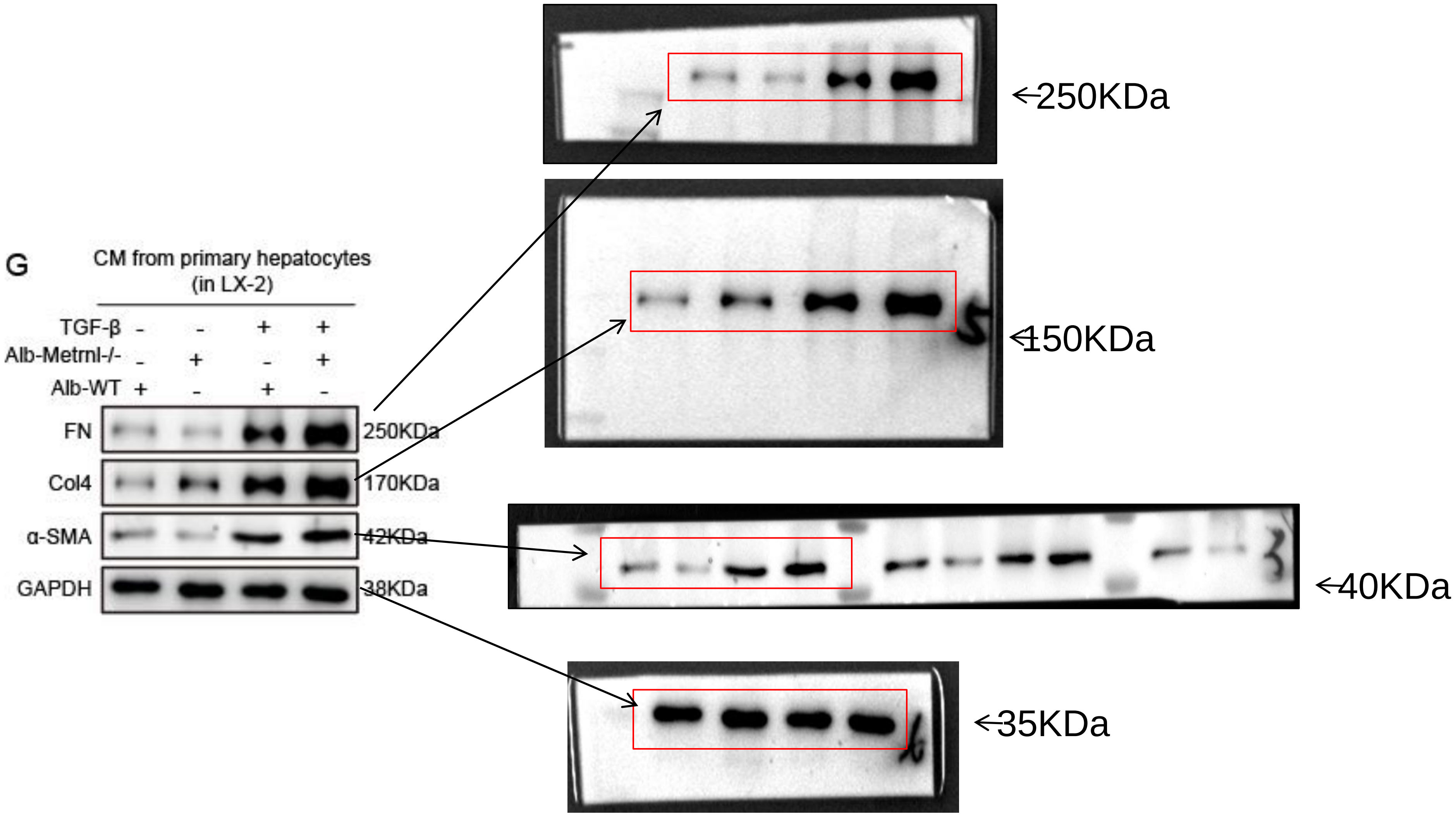
G
CM from primary hepatocytes
(in LX-2)
TGF-β - - + +
Alb-Metrnl-/- - + - +
Alb-WT + - + -
FN
Col4
α-SMA
GAPDH
250KDa
170KDa
42KDa
38KDa
250KDa
150KDa
40KDa
35KDa

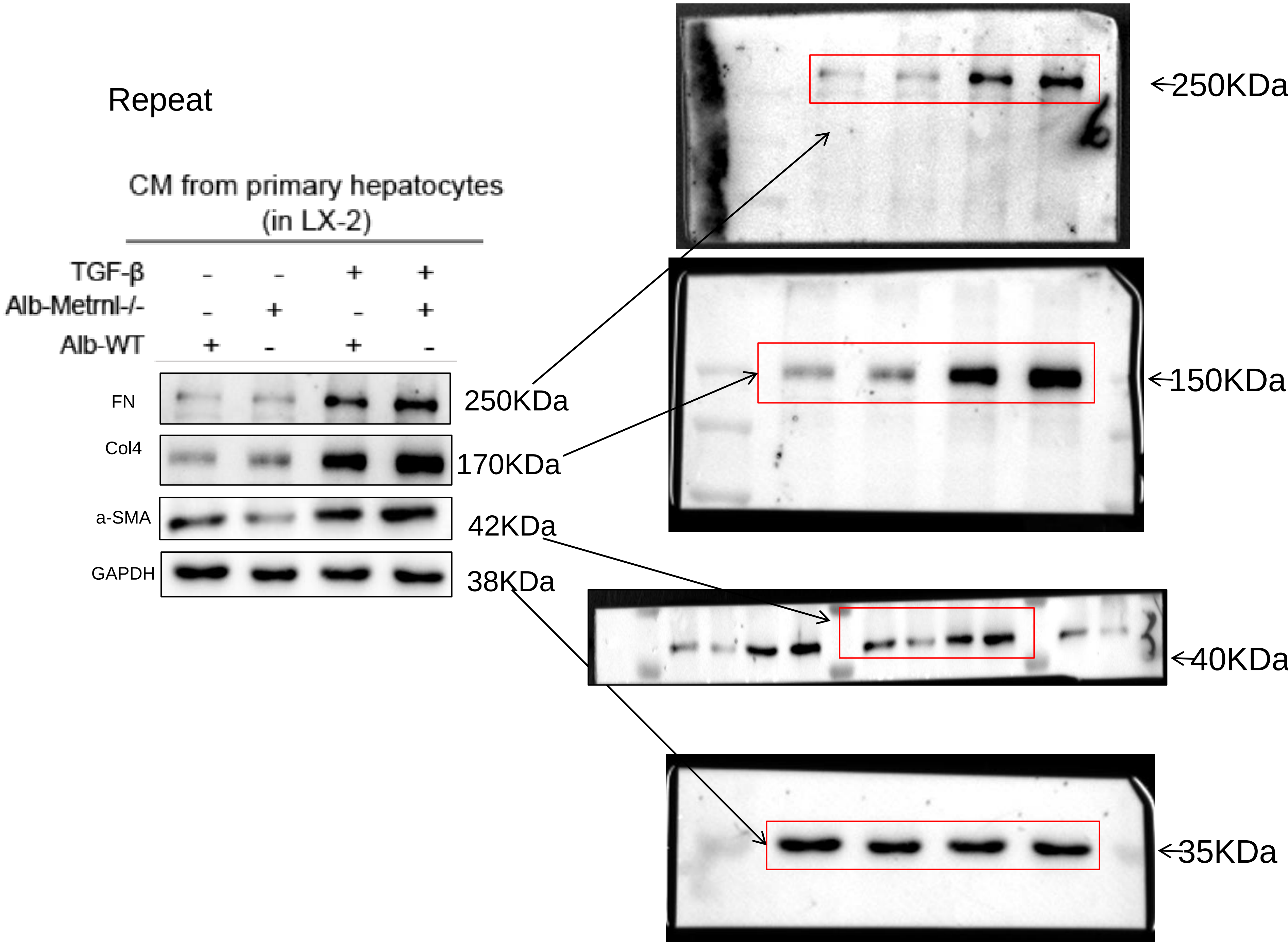

Repeat
CM from primary hepatocytes
(in LX-2)
TGF-β - - + +
Alb-Metrnl-/- - + - +
Alb-WT + - + -
FN
Col4
a-SMA
GAPDH
250KDa
170KDa
42KDa
38KDa
250KDa
150KDa
40KDa
35KDa

Repeat

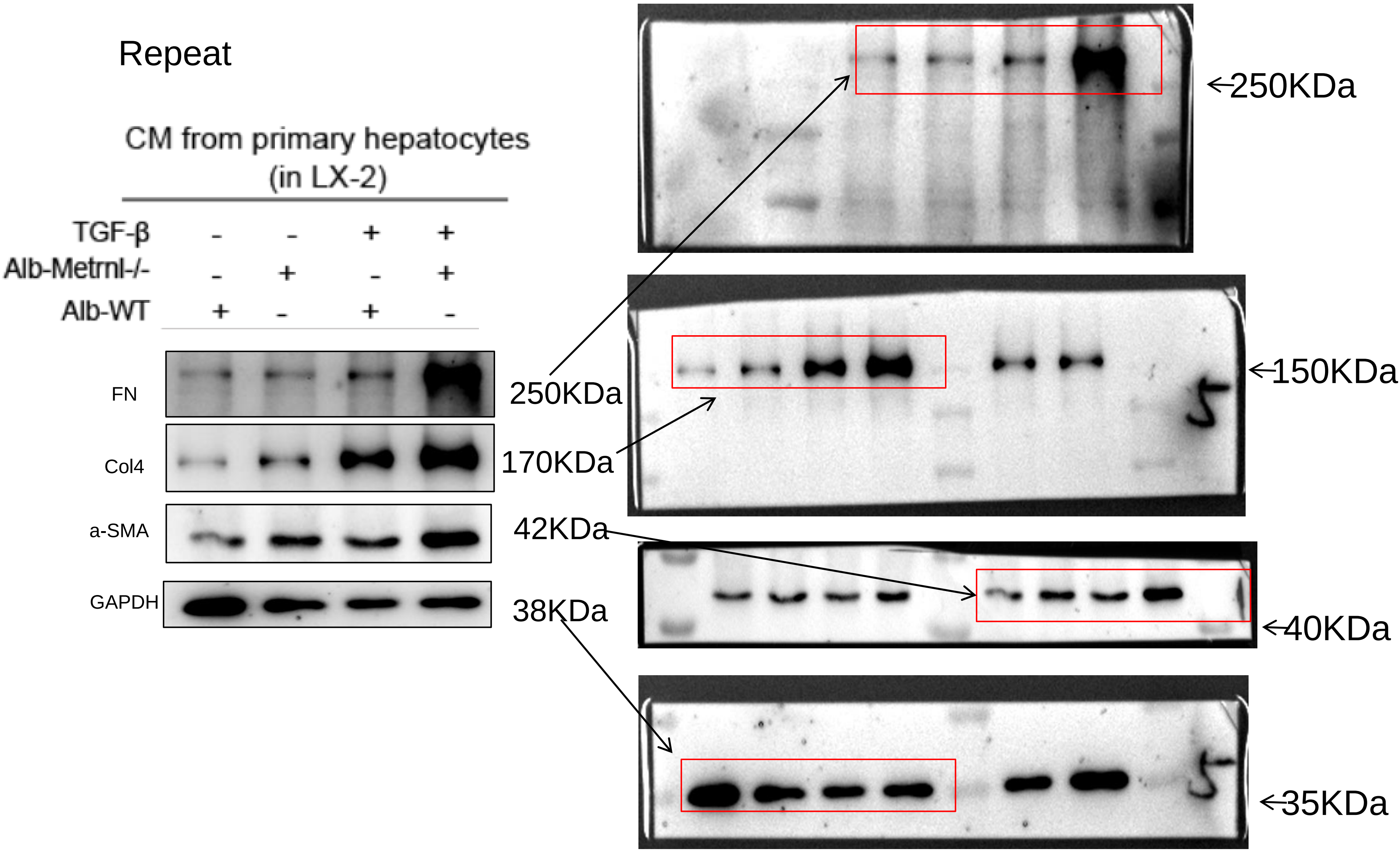

CM from primary hepatocytes
(in LX-2)
TGF-β
Alb-Metrnl-/-
Alb-WT
FN
Col4
a-SMA
GAPDH
250KDa
170KDa
42KDa
38KDa
250KDa
150KDa
40KDa
35KDa

Fig.6

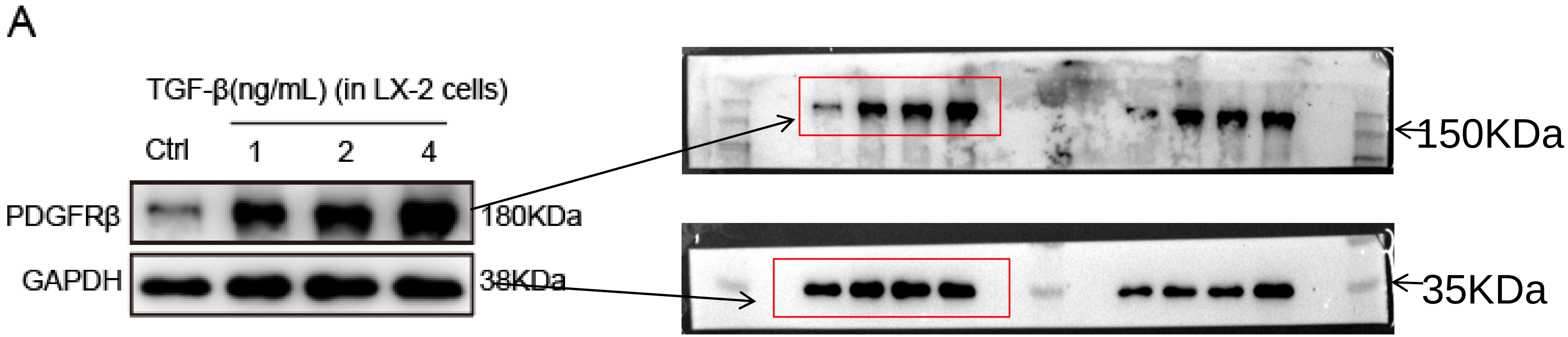
A
TGF-β(ng/mL) (in LX-2 cells)
Ctrl
1
2
4
PDGFRβ
GAPDH
180KDa
38KDa
150KDa
35KDa

Repeat

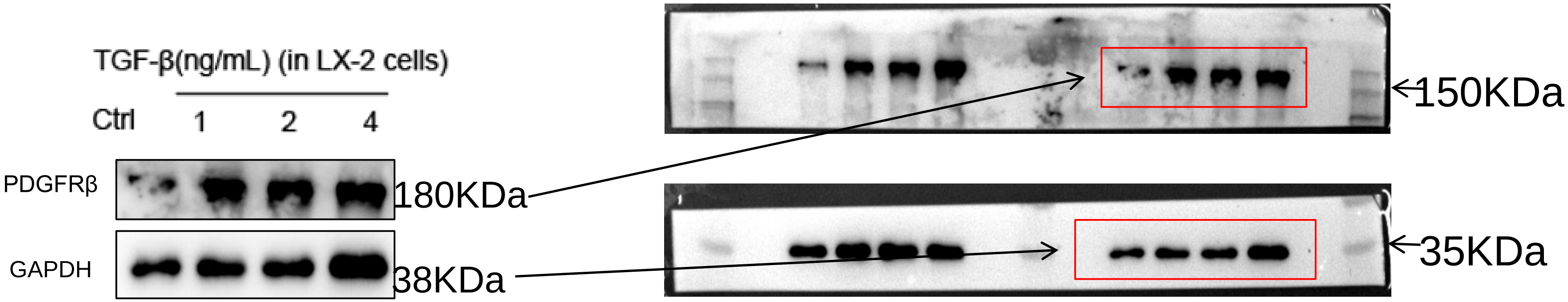

Repeat

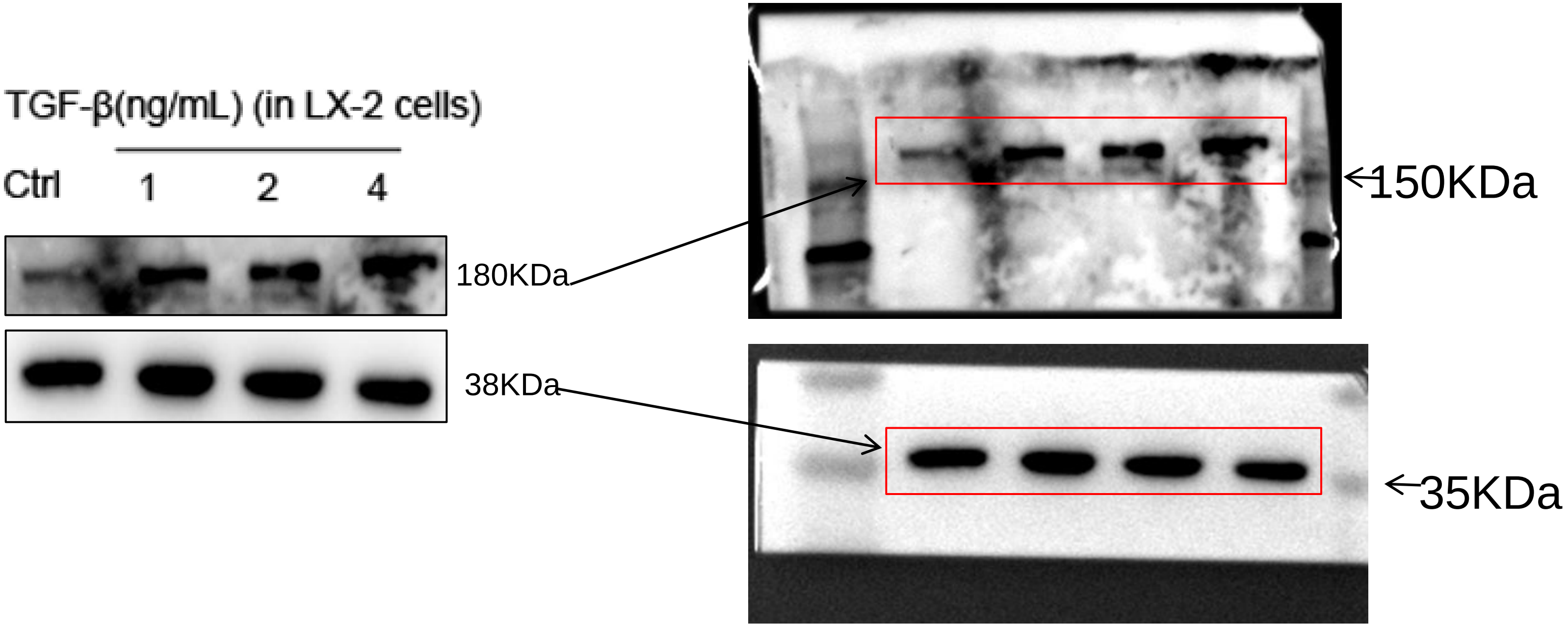

Fig.6

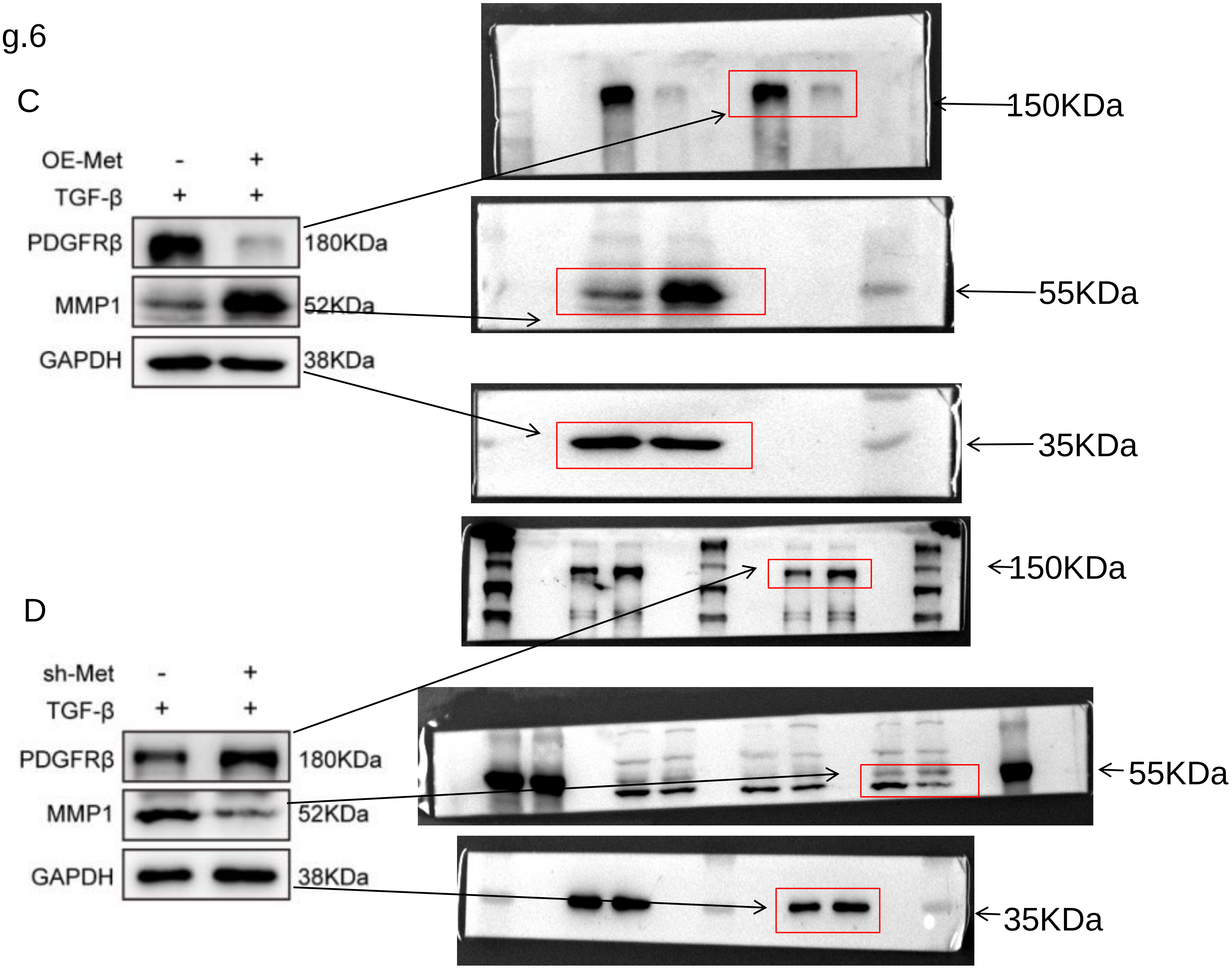
C
OE-Met - +
TGF-β + +
PDGFRβ
MMP1
GAPDH
180KDa
52KDa
38KDa
150KDa
55KDa
35KDa
D
sh-Met - +
TGF-β + +
PDGFRβ
MMP1
GAPDH
180KDa
52KDa
38KDa
150KDa
55KDa
35KDa

Repeat

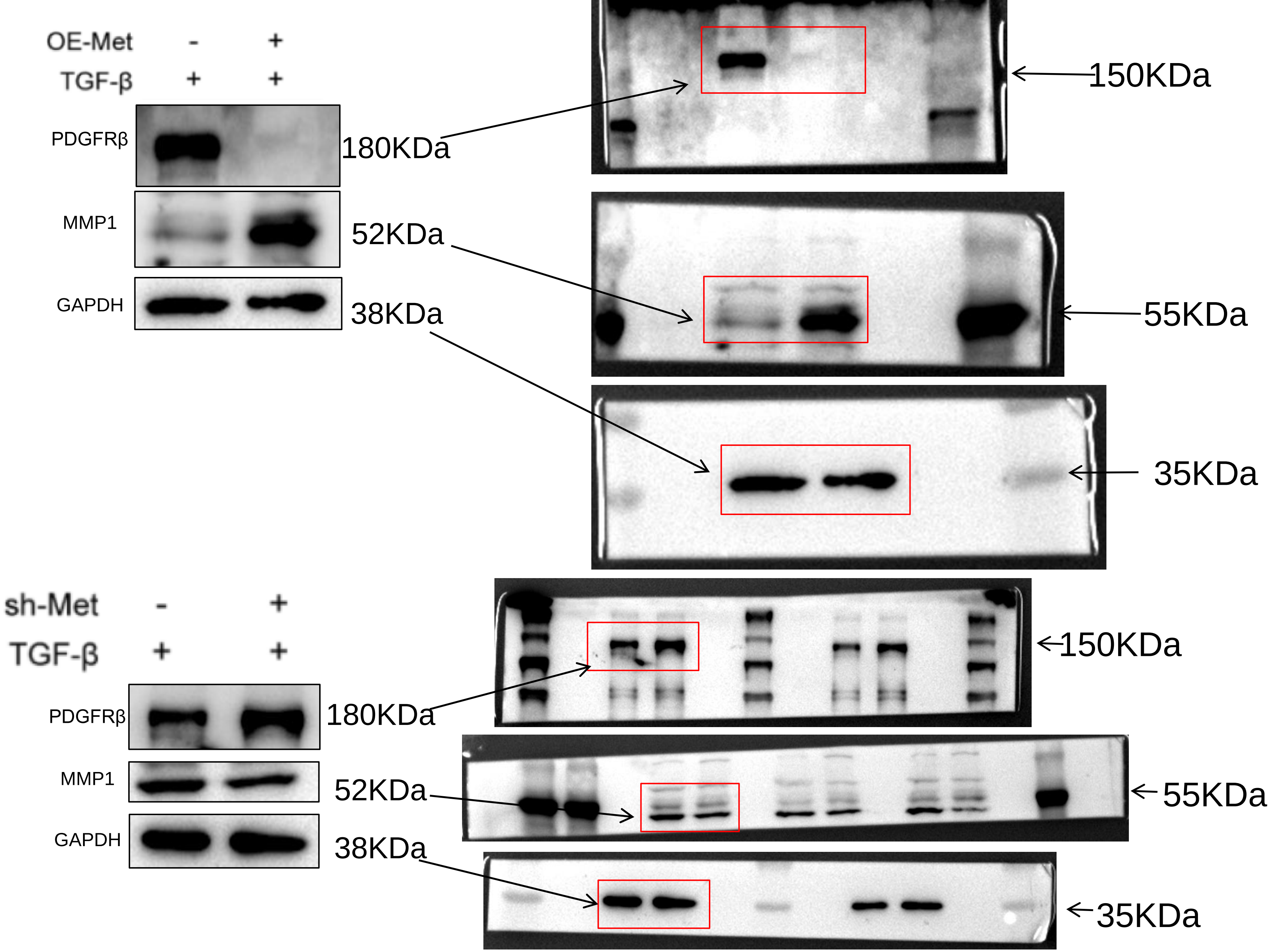

OE-Met - +
TGF-β + +
PDGFRβ
180KDa
MMP1
52KDa
GAPDH
38KDa
150KDa
55KDa
35KDa
sh-Met - +
TGF-β + +
PDGFRβ
180KDa
MMP1
52KDa
GAPDH
38KDa
150KDa
55KDa
35KDa

Repeat

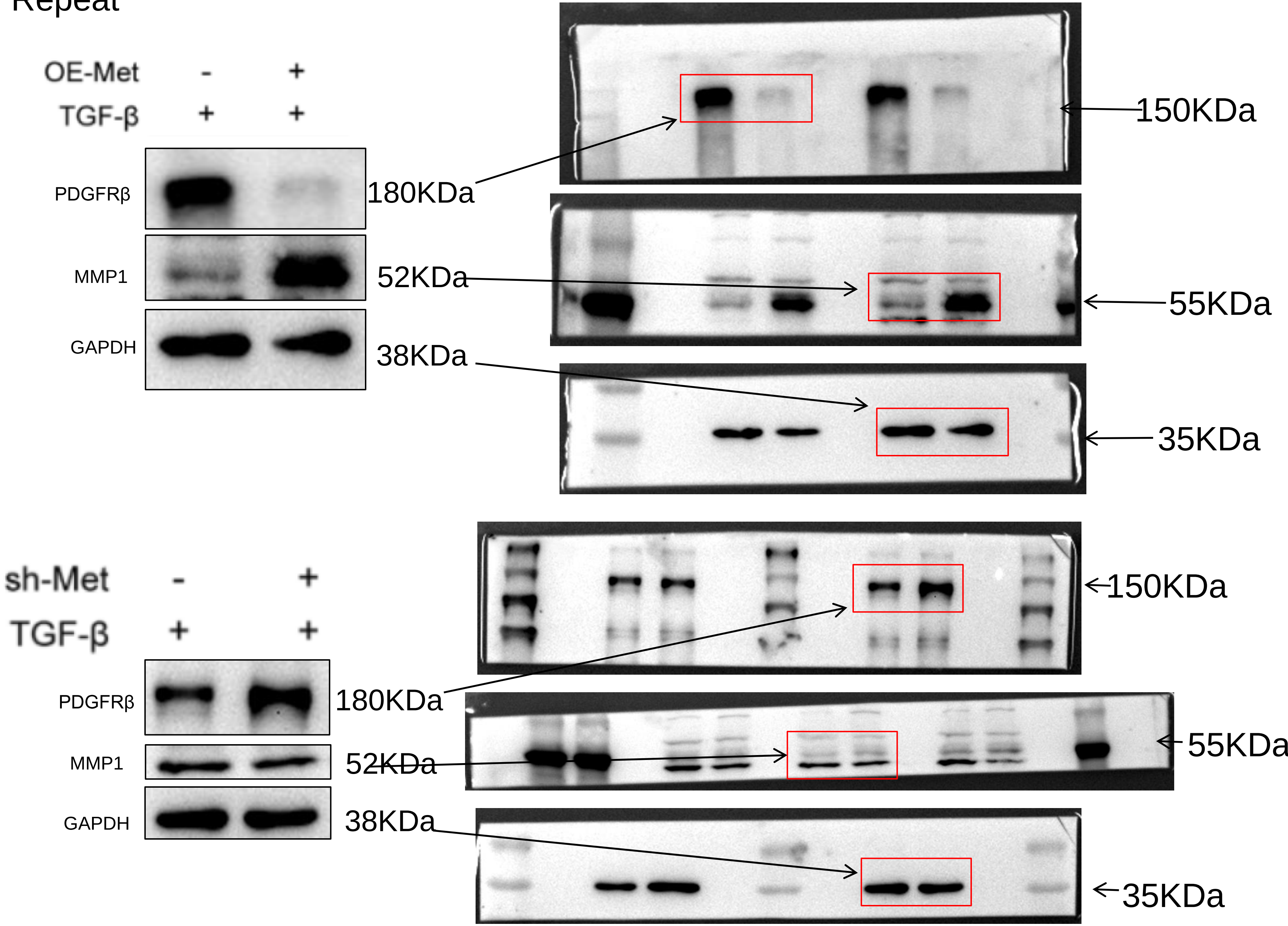
OE-Met - +
TGF-β + +
PDGFRβ
MMP1
GAPDH
180KDa
52KDa
38KDa
150KDa
55KDa
35KDa
sh-Met - +
TGF-β + +
PDGFRβ
MMP1
GAPDH
180KDa
52KDa
38KDa
150KDa
55KDa
35KDa

Fig.6

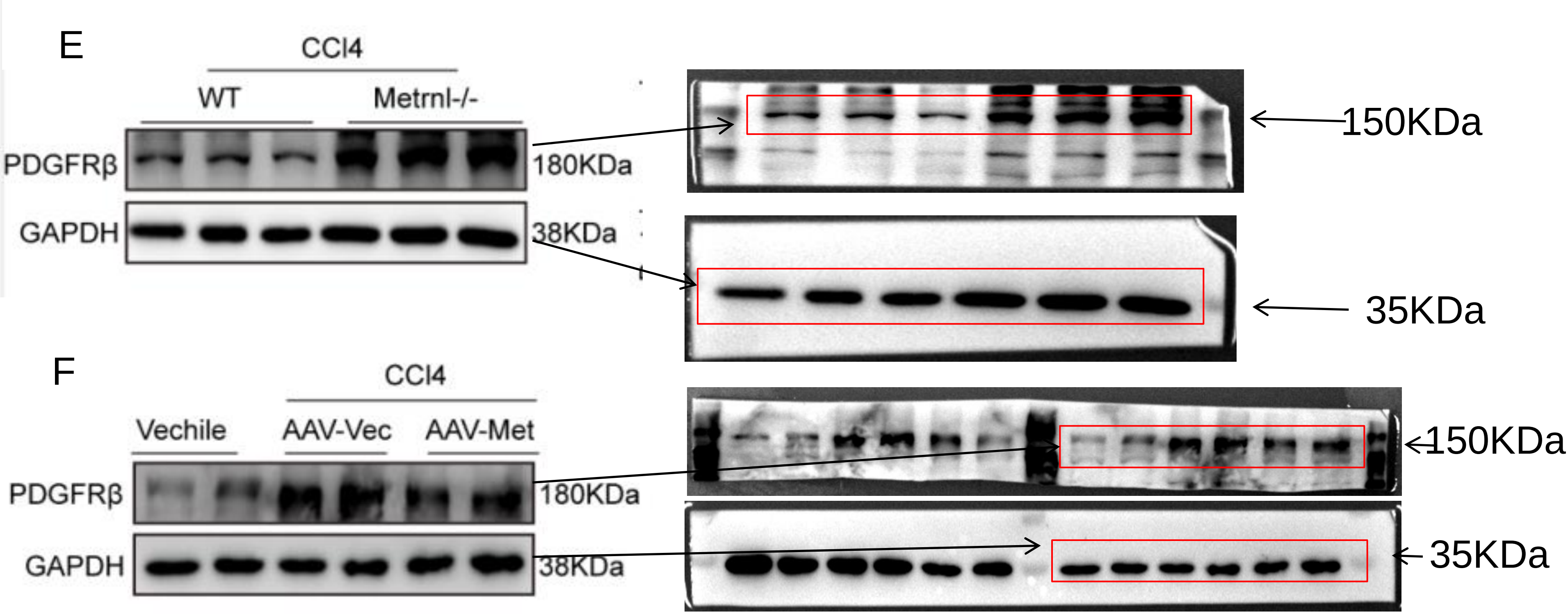
E
CCl4
WT
Metrnl-/-
PDGFRβ
180KDa
GAPDH
38KDa
150KDa
35KDa
F
CCl4
Vechile
AAV-Vec
AAV-Met
PDGFRβ
180KDa
GAPDH
38KDa
150KDa
35KDa

Repeat

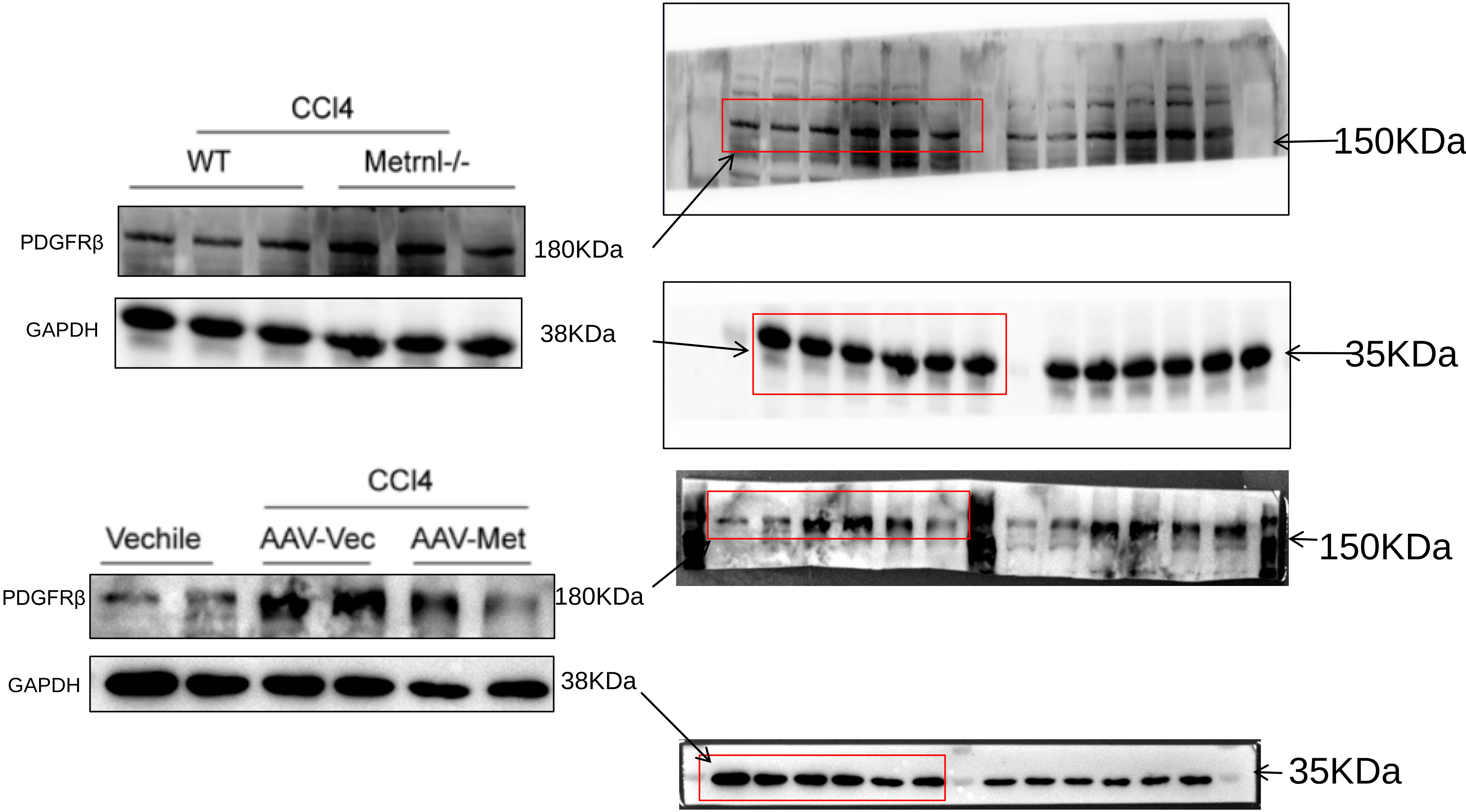
CCl4
WT
Metrnl-/-
PDGFRβ
180KDa
GAPDH
38KDa
150KDa
35KDa
CCl4
Vechile
AAV-Vec
AAV-Met
PDGFRβ
180KDa
GAPDH
38KDa
150KDa
35KDa

Repeat

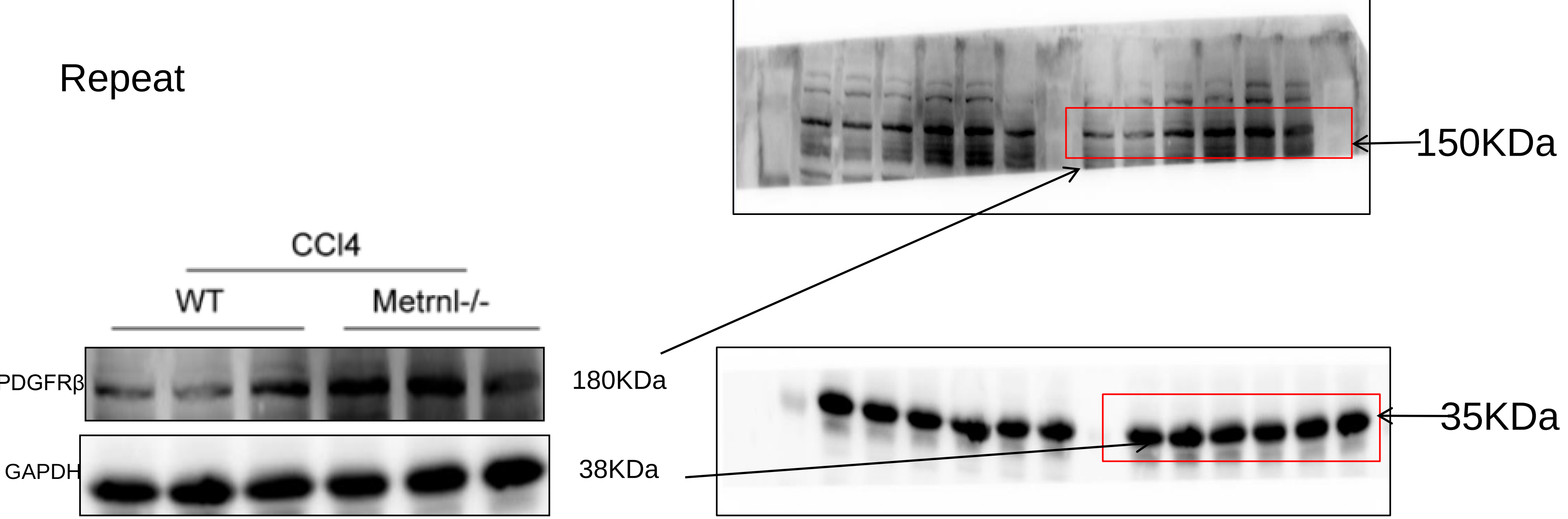

CCl4
WT
Metrnl-/-
PDGFRβ
GAPDH
180KDa
38KDa
150KDa
35KDa

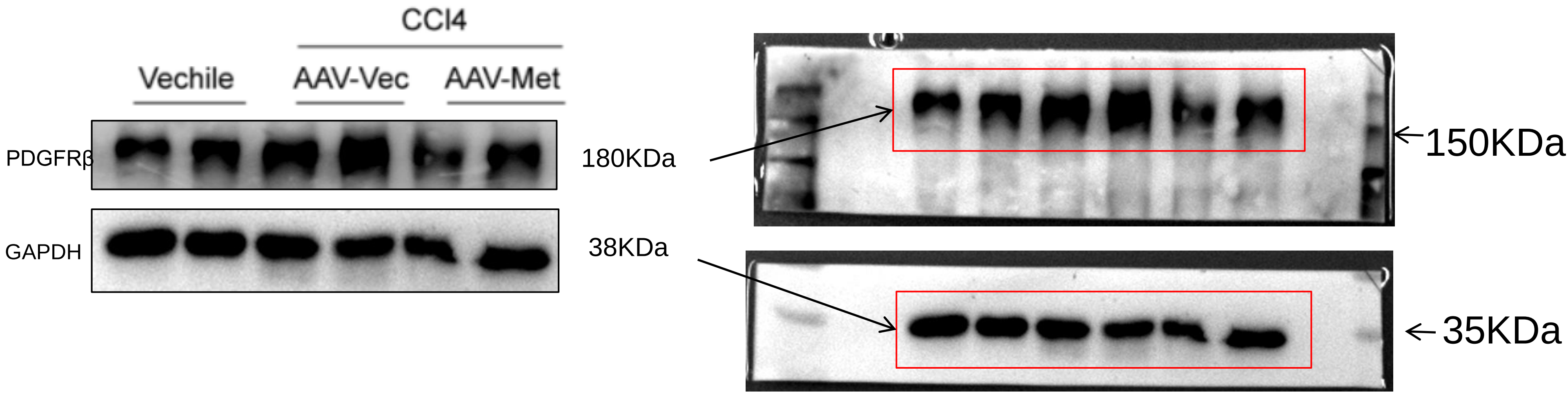

CCl4
Vechile
AAV-Vec
AAV-Met
PDGFRβ
GAPDH
180KDa
38KDa
150KDa
35KDa

Fig.6

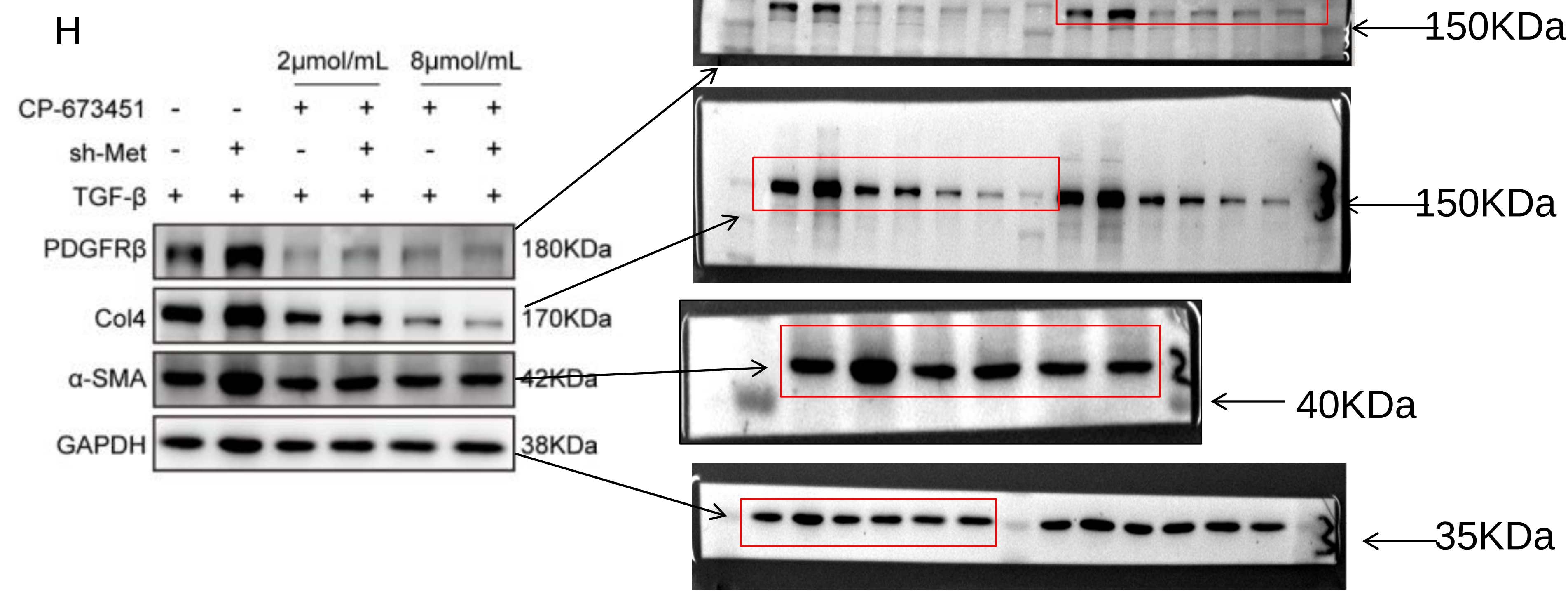

H
2μmol/mL
8μmol/mL
CP-673451 - - + + + +
sh-Met - + - + - +
TGF-β + + + + + +
PDGFRβ
180KDa
Col4
170KDa
α-SMA
42KDa
GAPDH
38KDa
150KDa
150KDa
40KDa
35KDa

Repeat

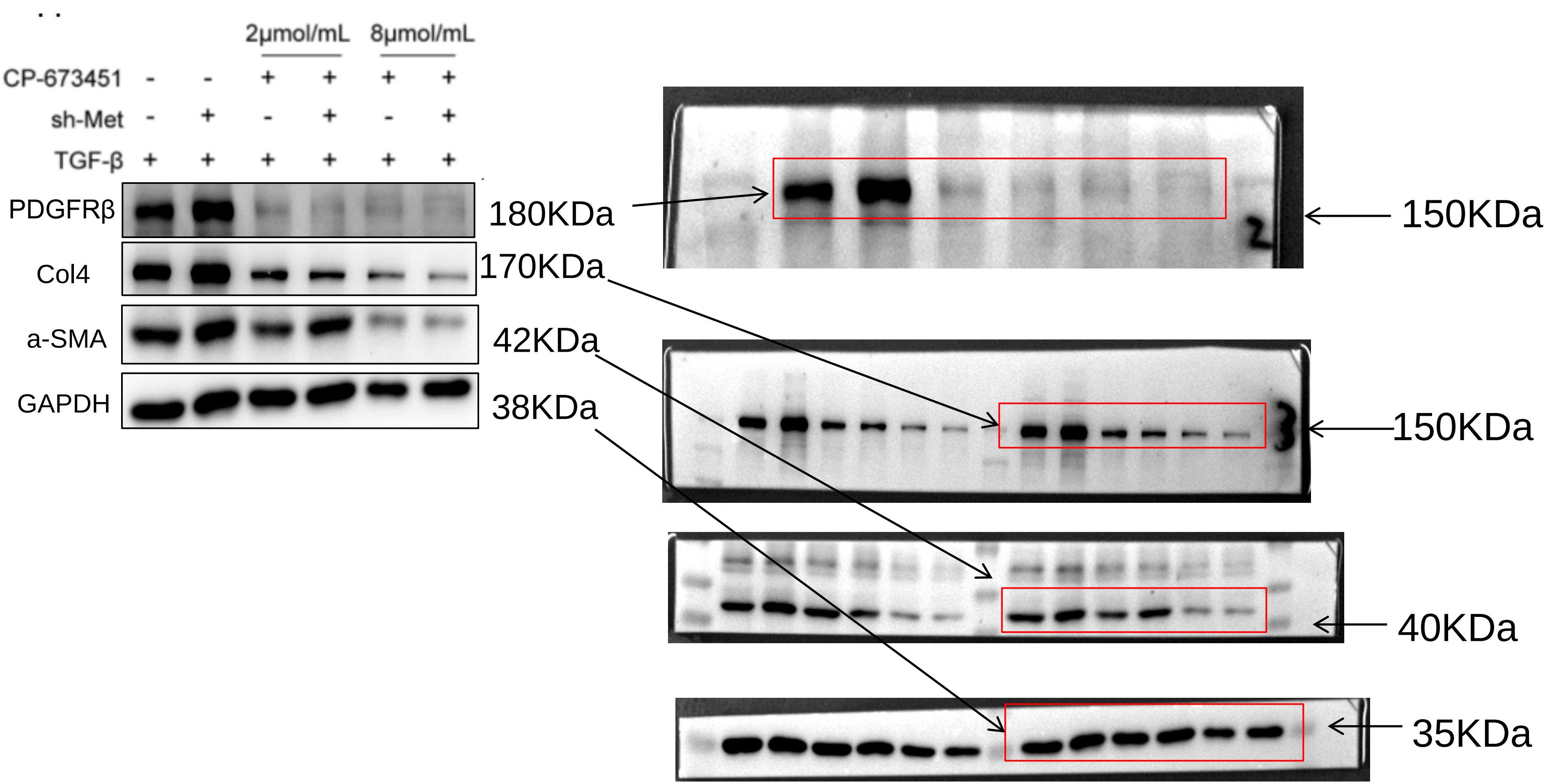
2μmol/mL
8μmol/mL
CP-673451 - - + + + +
sh-Met - + - + - +
TGF-β + + + + + +
PDGFRβ
Col4
a-SMA
GAPDH
180KDa
170KDa
42KDa
38KDa
150KDa
150KDa
40KDa
35KDa

Repeat

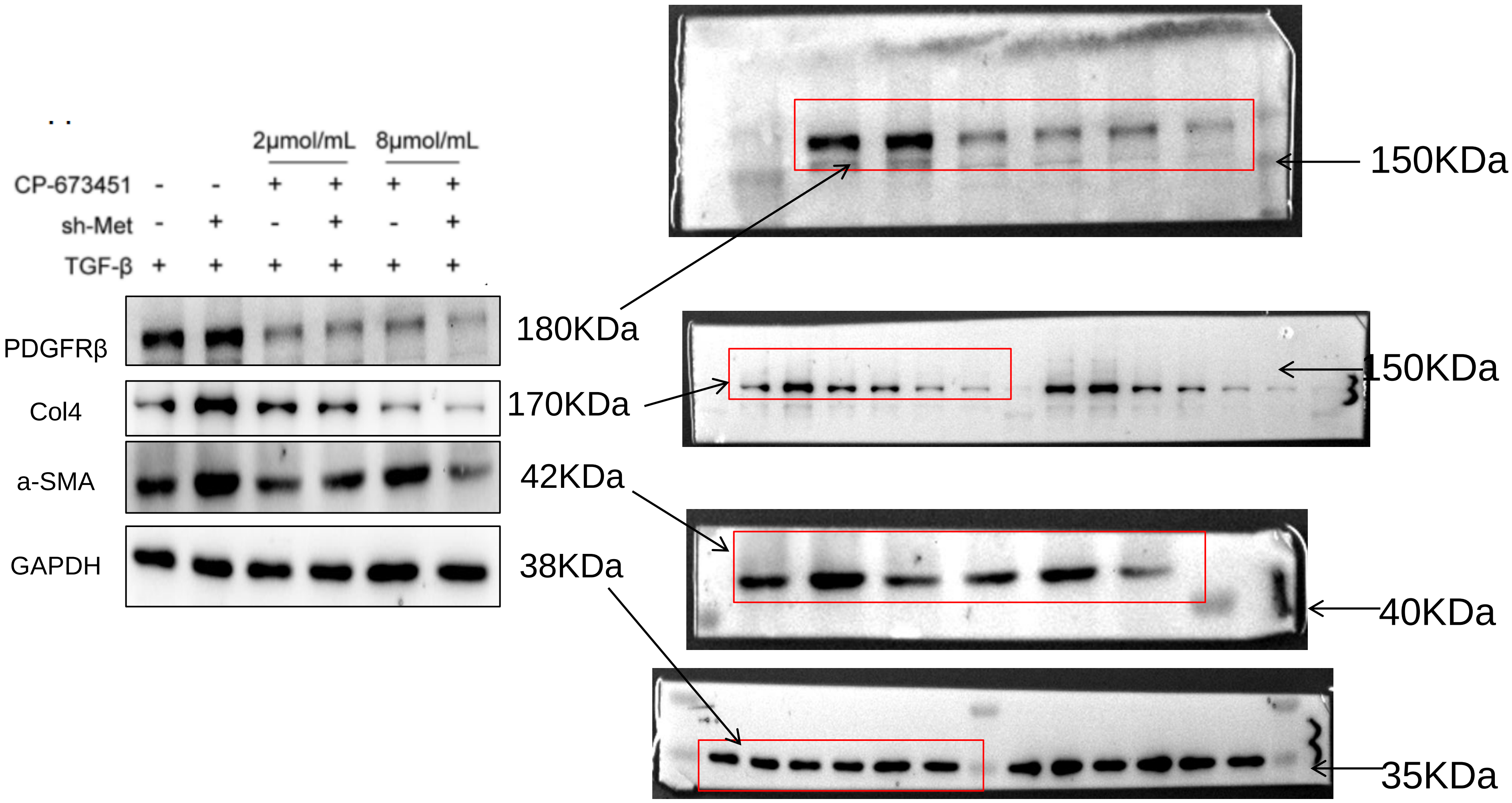
2μmol/mL
8μmol/mL
CP-673451 - - + + + +
sh-Met - + - + - +
TGF-β + + + + + +
PDGFRβ
Col4
a-SMA
GAPDH
180KDa
170KDa
42KDa
38KDa
150KDa
150KDa
40KDa
35KDa

Fig.7

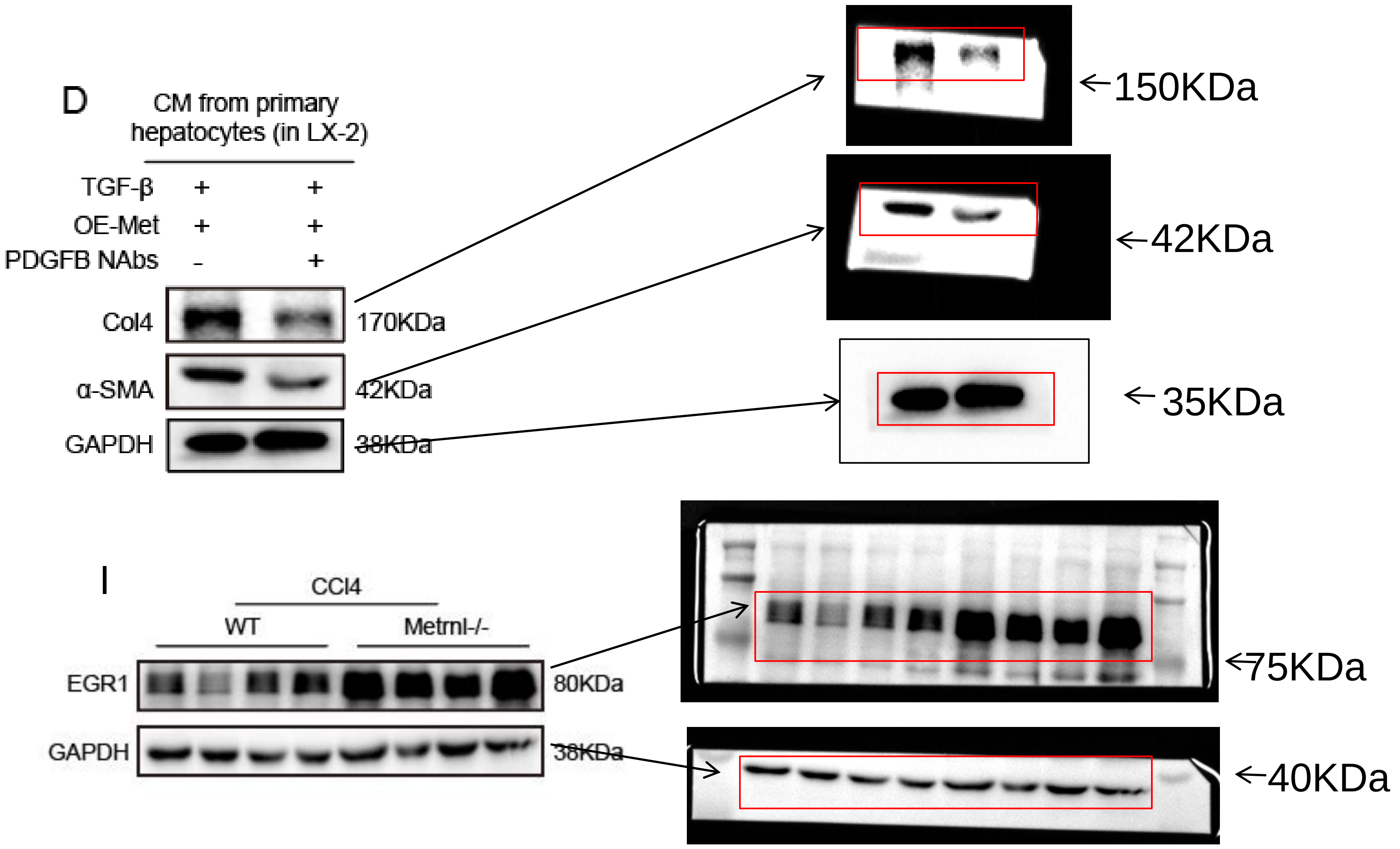
D
CM from primary hepatocytes (in LX-2)
TGF-β + +
OE-Met + +
PDGFB NAbs - +
Col4
170KDa
α-SMA
42KDa
GAPDH
38KDa
150KDa
42KDa
35KDa
I
CCl4
WT
Metrnl-/-
EGR1
80KDa
GAPDH
38KDa
75KDa
40KDa

Repeat

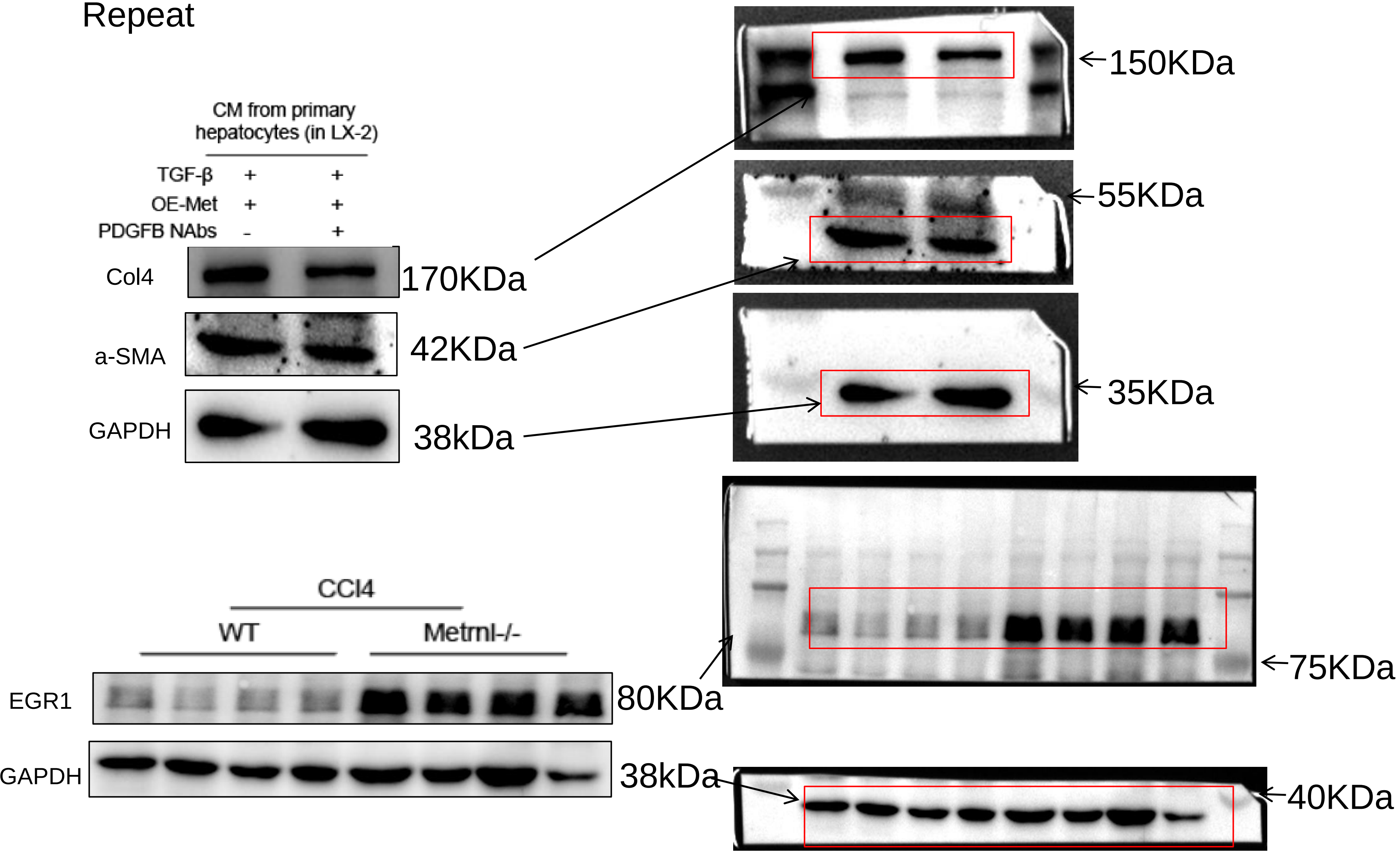

CM from primary hepatocytes (in LX-2)
TGF-β + +
OE-Met + +
PDGFB NAbs - +
Col4
170KDa
a-SMA
42KDa
GAPDH
38kDa
150KDa
55KDa
35KDa
CCl4
WT
Metrnl-/-
EGR1
80KDa
GAPDH
38kDa
75KDa
40KDa

Repeat

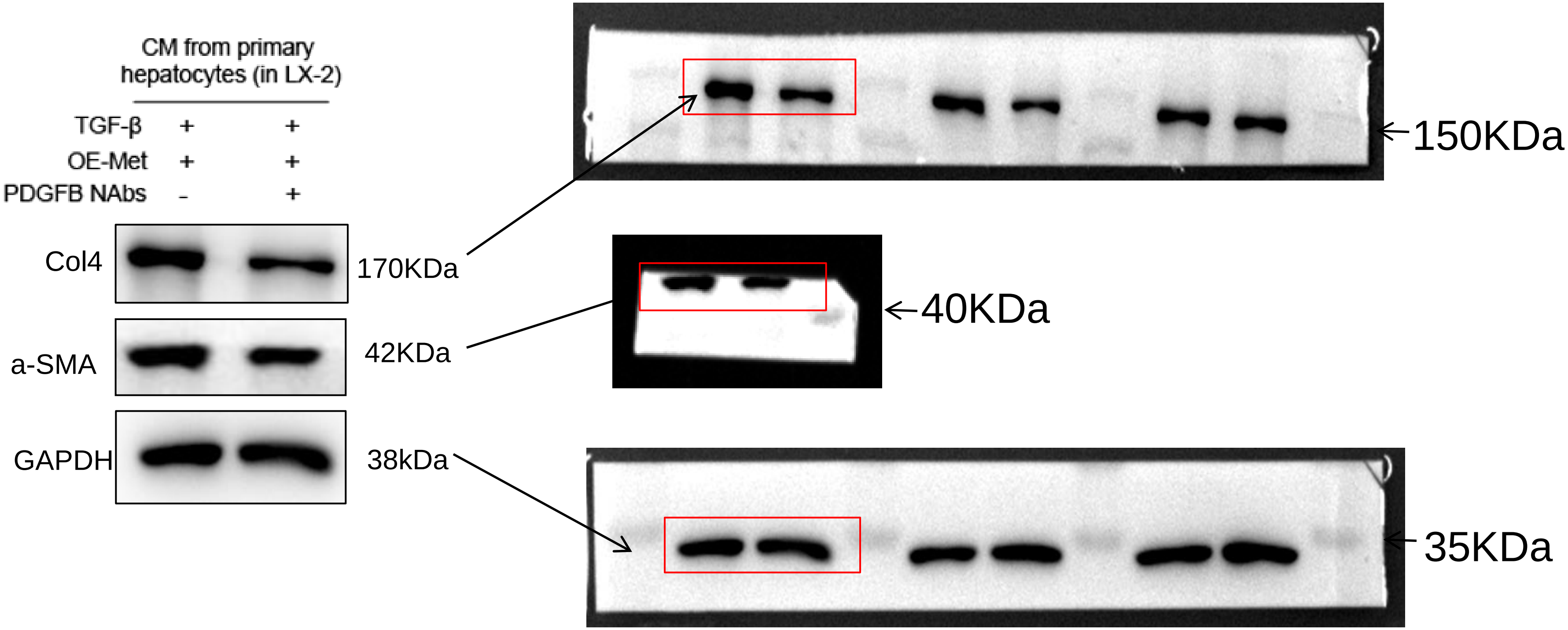

CM from primary hepatocytes (in LX-2)
TGF-β + +
OE-Met + +
PDGFB NAbs - +
Col4
a-SMA
GAPDH
170KDa
42KDa
38kDa
150KDa
40KDa
35KDa

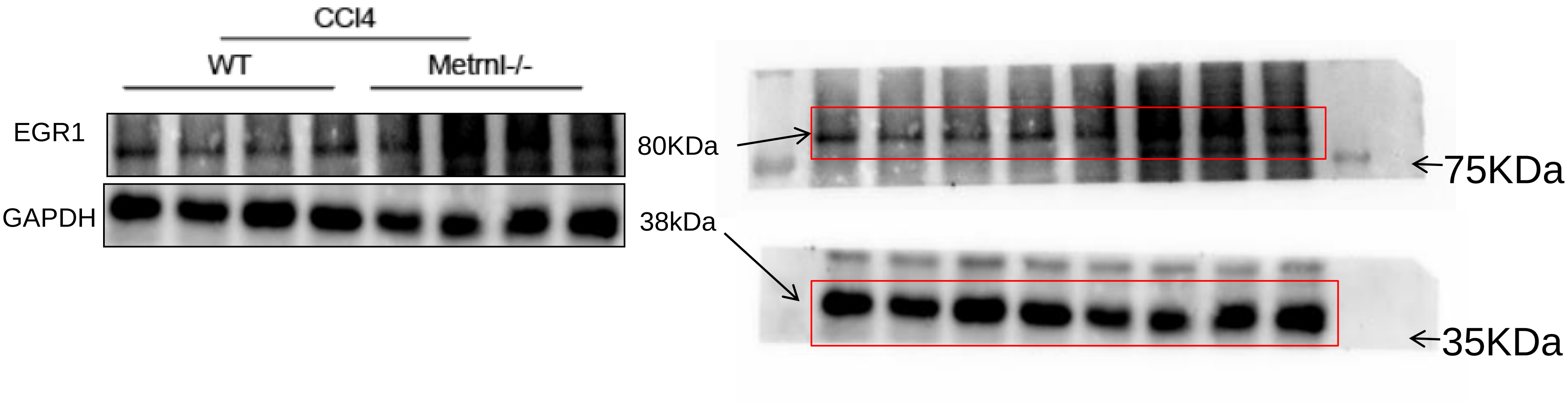

CCl4
WT
Metrnl-/-
EGR1
GAPDH
80KDa
38kDa
75KDa
35KDa

Fig.8

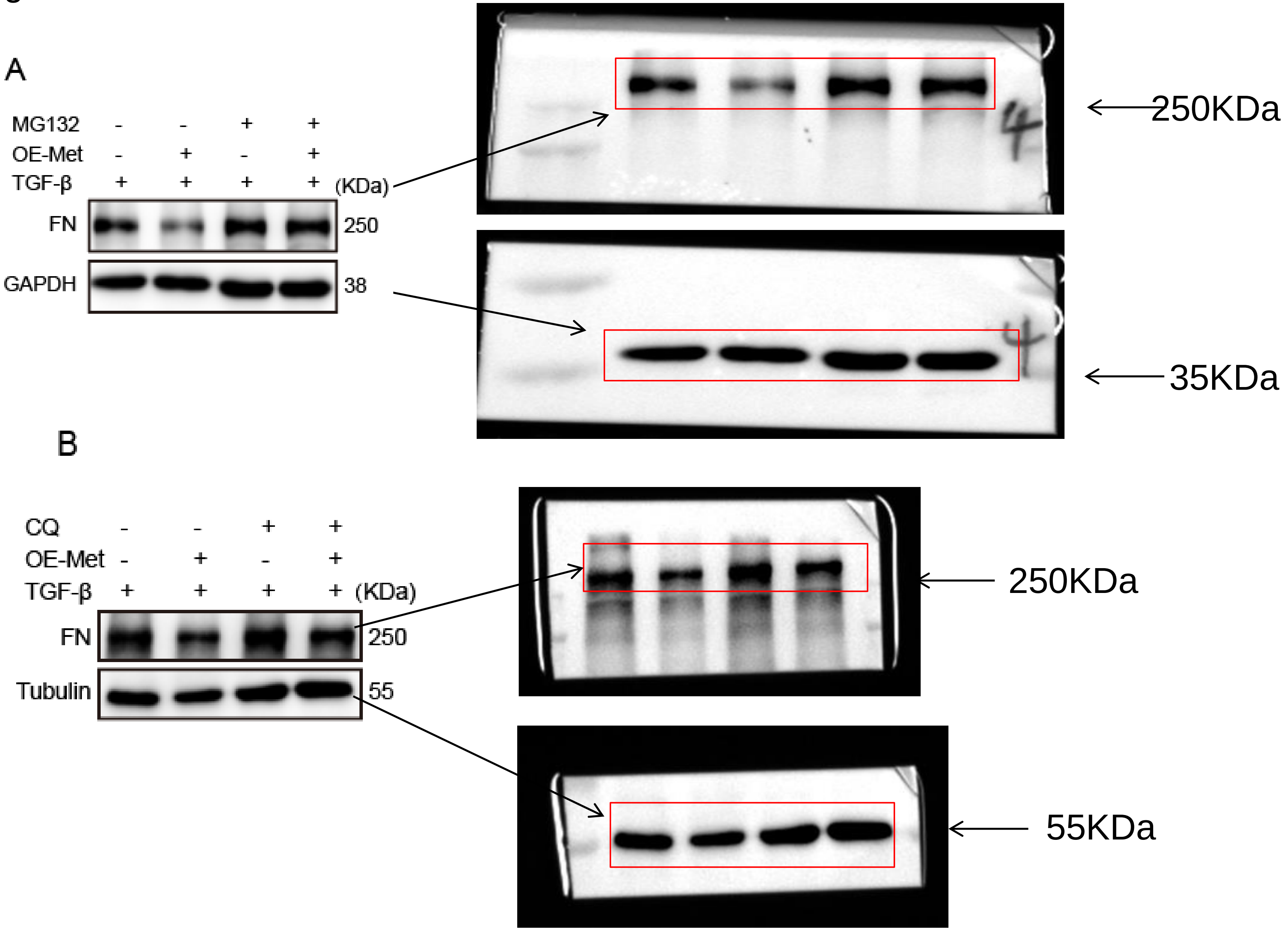
A
MG132 - - + +
OE-Met - + - +
TGF-β + + + +
(KDa)
FN
250
GAPDH
38
250KDa
35KDa
B
CQ - - + +
OE-Met - + - +
TGF-β + + + +
(KDa)
FN
250
Tubulin
55
250KDa
55KDa

Repeat

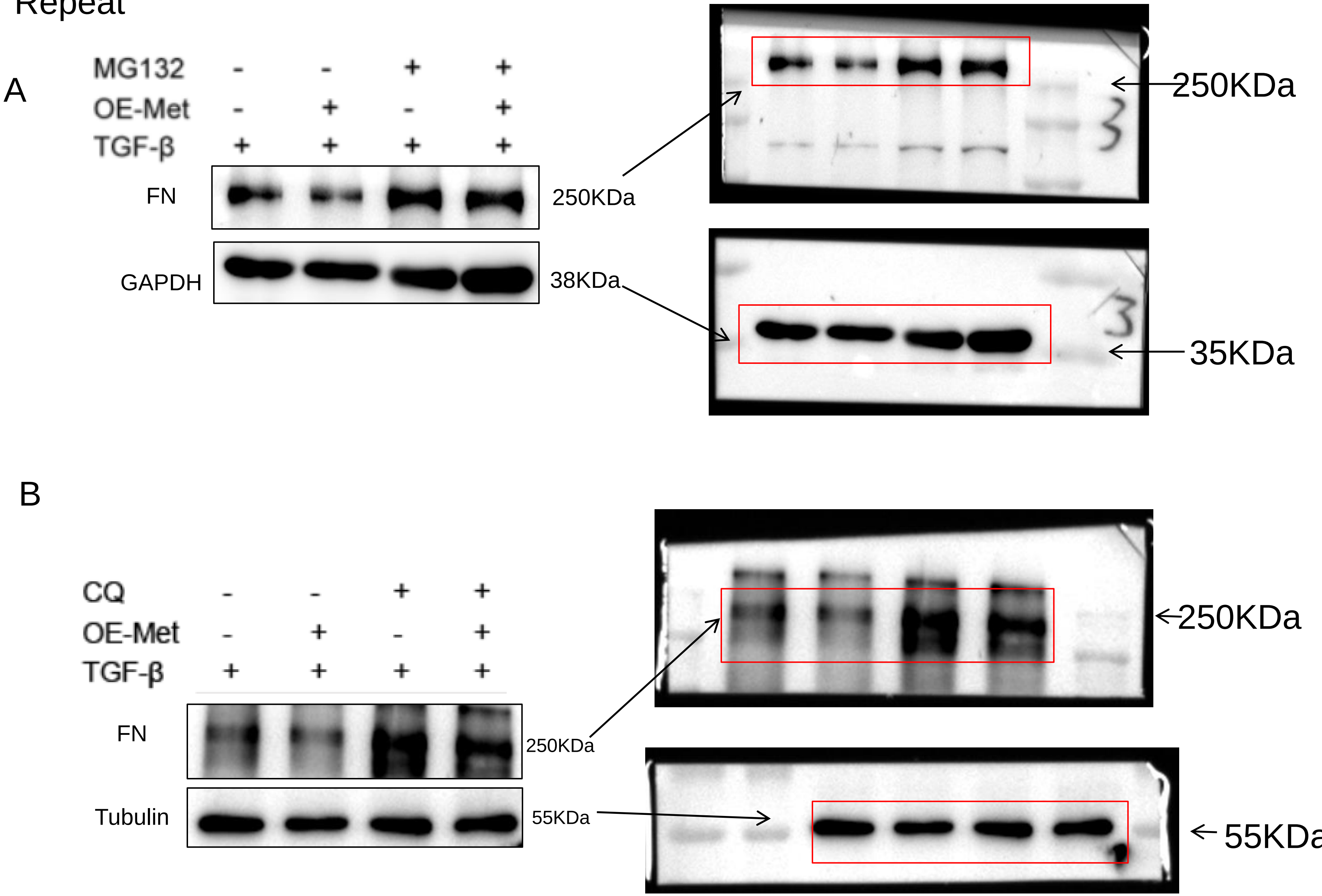

A
MG132 - - + +
OE-Met - + - +
TGF-β + + + +
FN
250KDa
GAPDH
38KDa
250KDa
35KDa
B
CQ - - + +
OE-Met - + - +
TGF-β + + + +
FN
250KDa
Tubulin
55KDa
250KDa
55KDa

Repeat

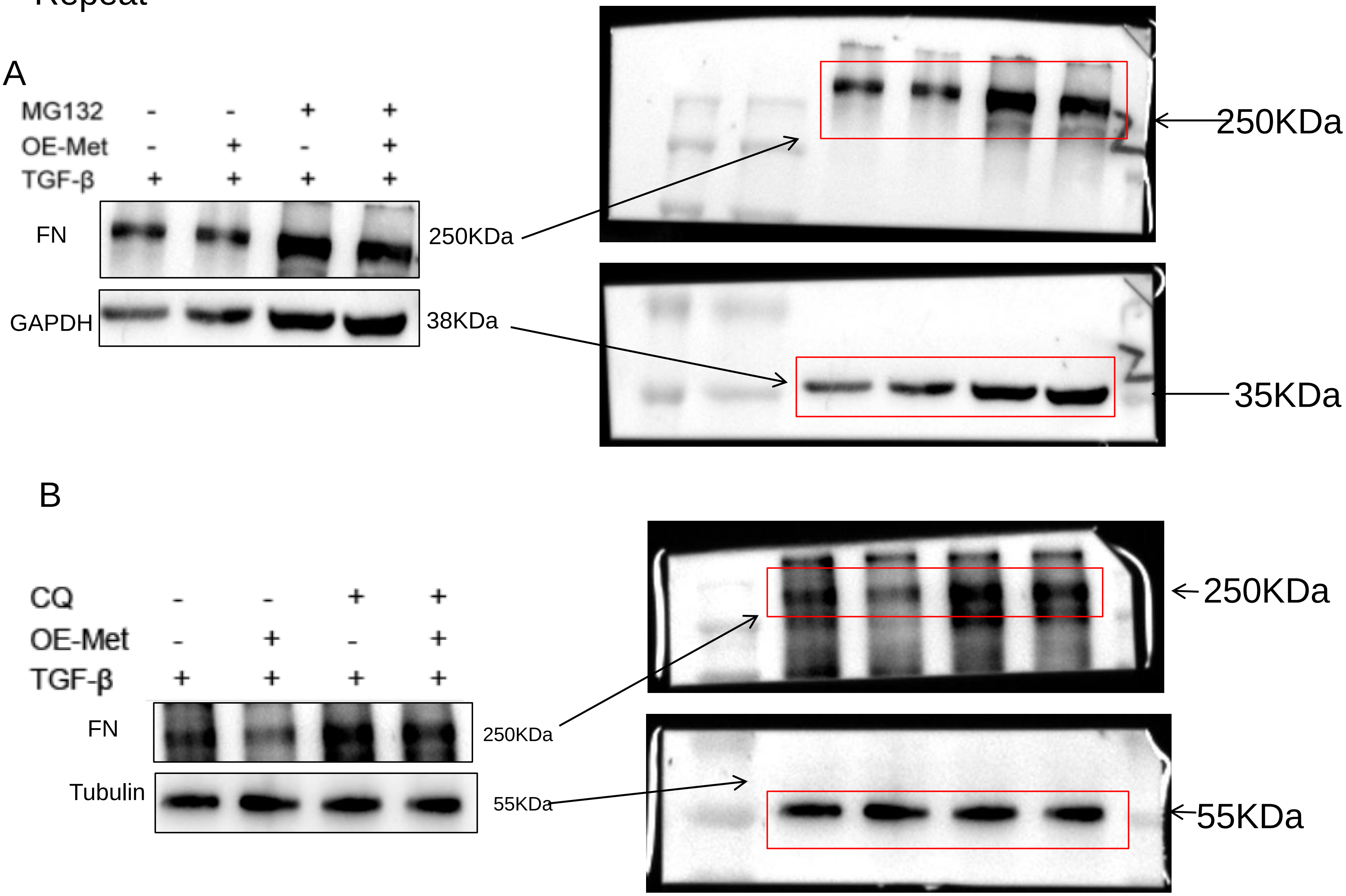
A
MG132 - - + +
OE-Met - + - +
TGF-β + + + +
FN
250KDa
GAPDH
38KDa
250KDa
35KDa
B
CQ - - + +
OE-Met - + - +
TGF-β + + + +
FN
250KDa
Tubulin
55KDa
250KDa
55KDa

Fig.8

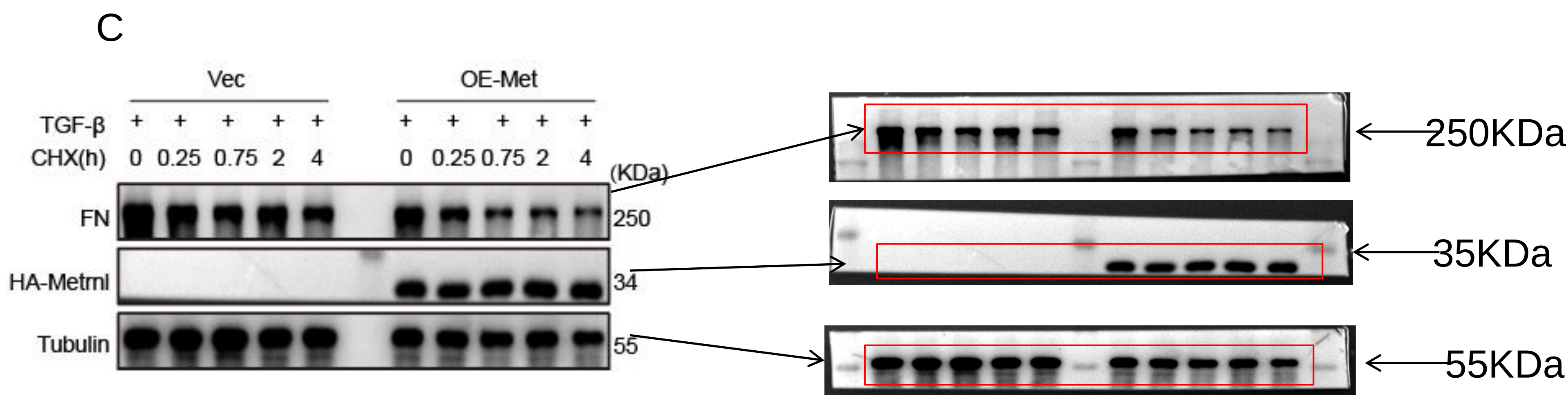
C
Vec
OE-Met
TGF-β + + + + + + + + + +
CHX(h) 0 0.25 0.75 2 4 0 0.25 0.75 2 4
(KDa)
FN
250
HA-Metrnl
34
Tubulin
55
250KDa
35KDa
55KDa

Repeat

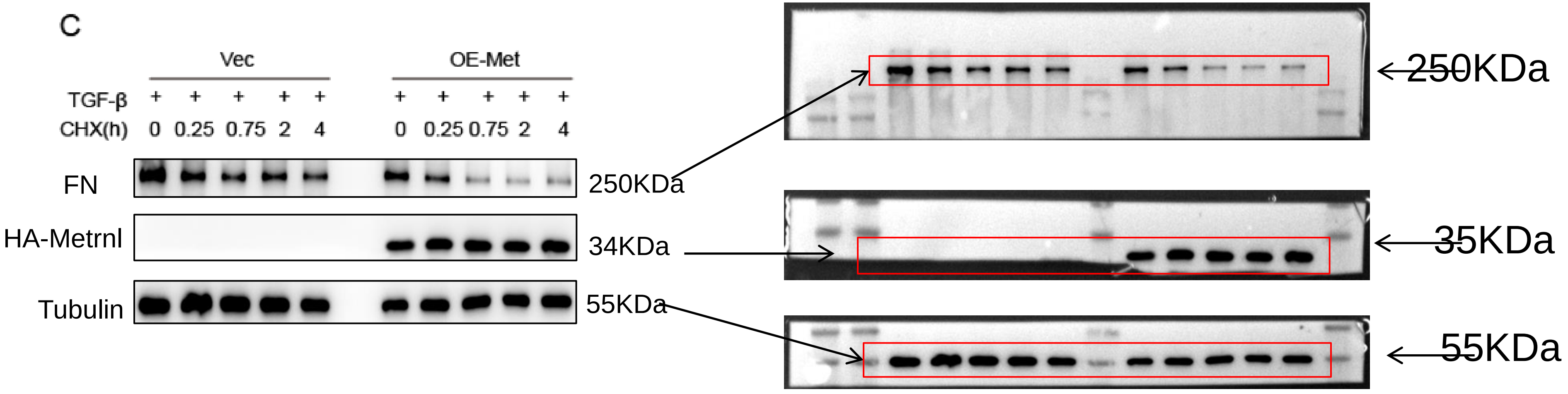

Repeat

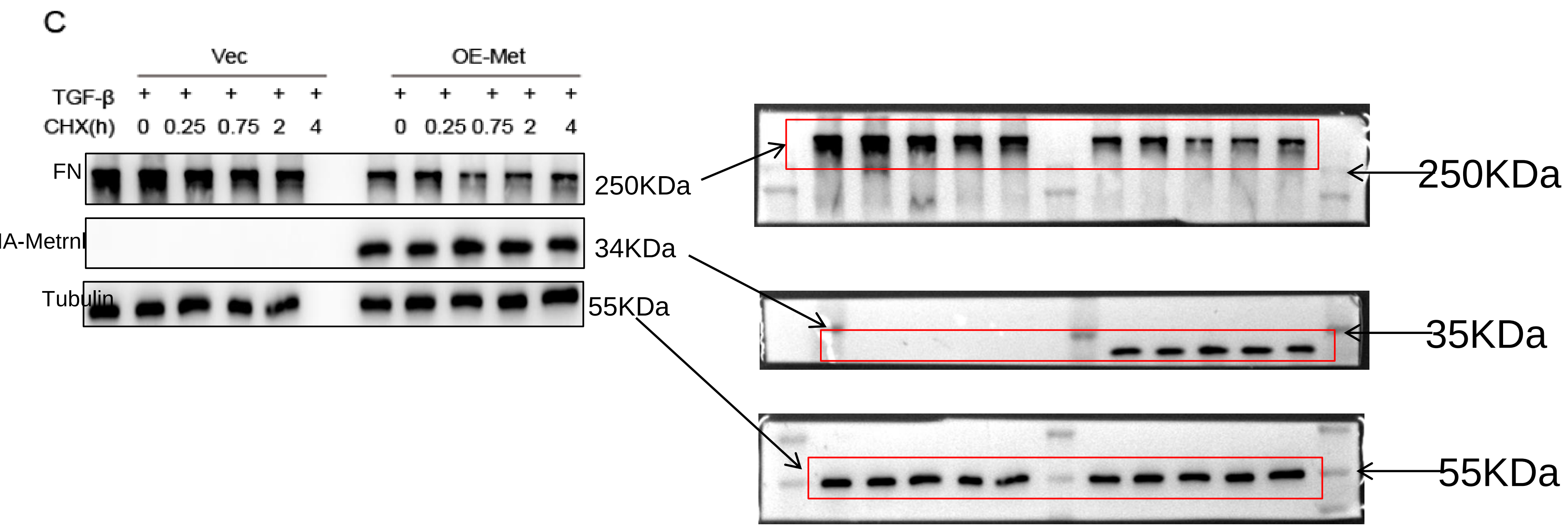

Fig.8

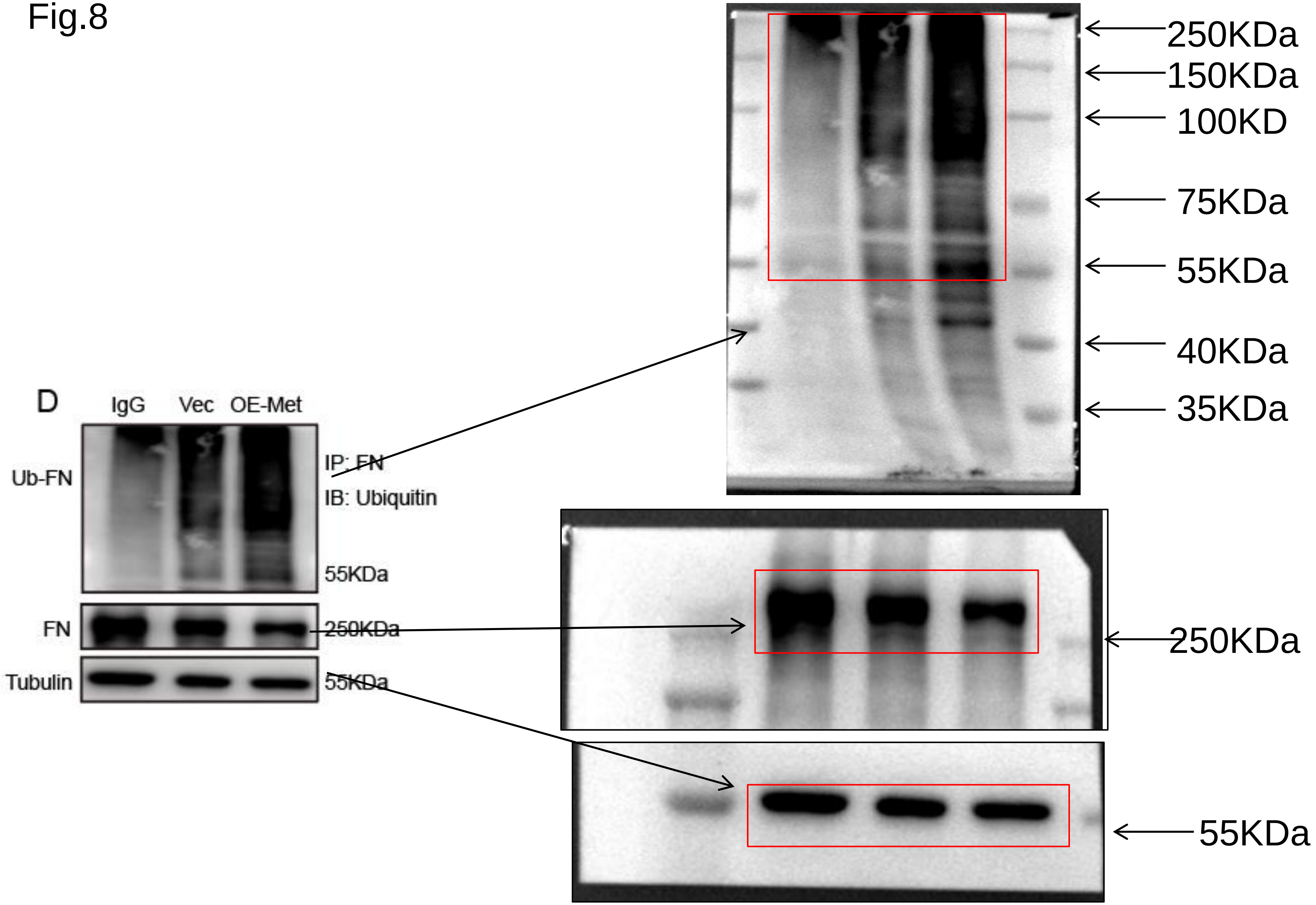
D
IgG
Vec
OE-Met
Ub-FN
FN
Tubulin
IP: FN
IB: Ubiquitin
55KDa
250KDa
55KDa
250KDa
150KDa
100KD
75KDa
55KDa
40KDa
35KDa
250KDa
55KDa

Repeat

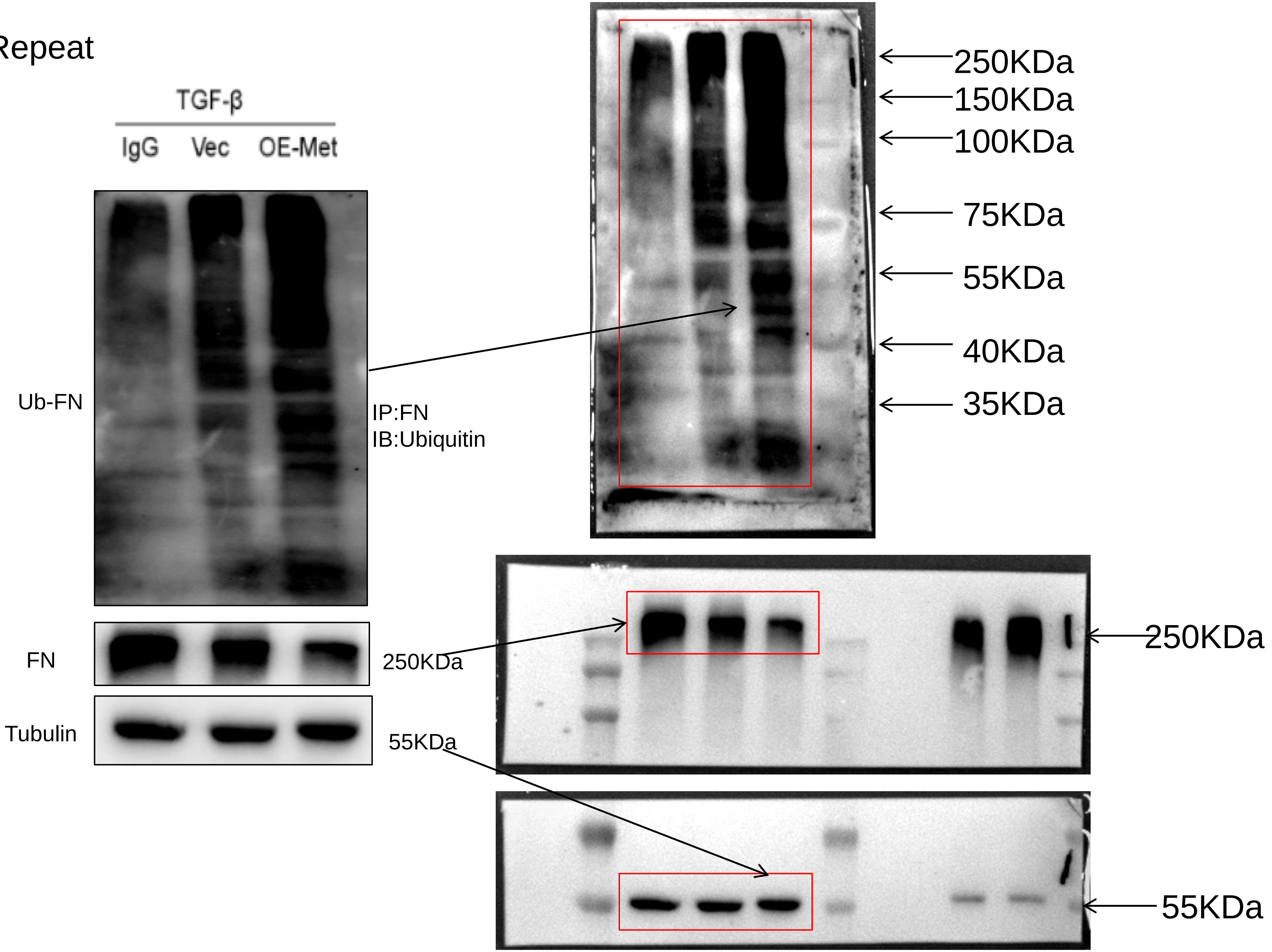
TGF-β
IgG
Vec
OE-Met
Ub-FN
IP:FN
IB:Ubiquitin
FN
Tubulin
250KDa
55KDa
250KDa
150KDa
100KDa
75KDa
55KDa
40KDa
35KDa
250KDa
55KDa

Repeat

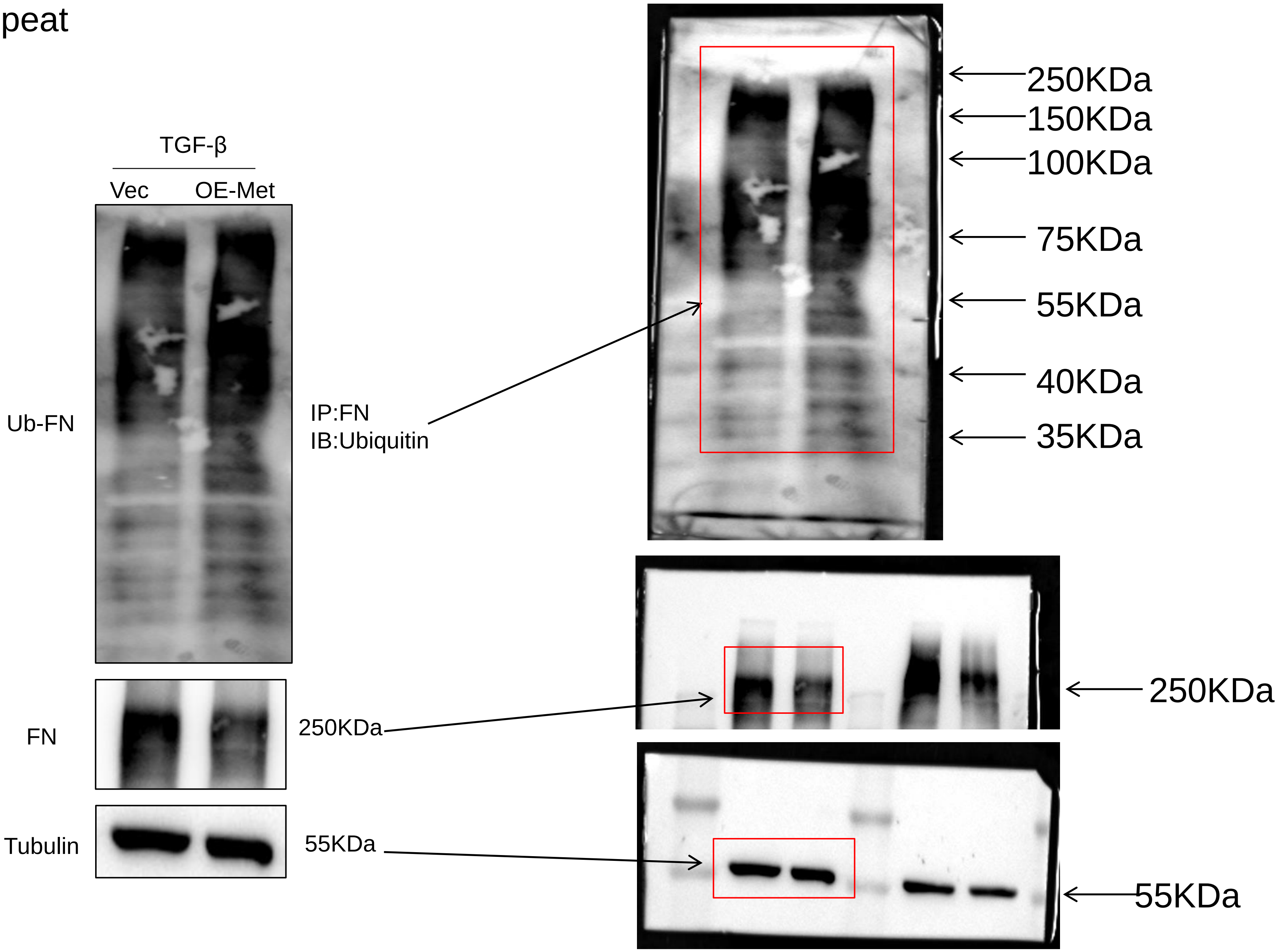
TGF-β
Vec
OE-Met
Ub-FN
IP:FN
IB:Ubiquitin
FN
250KDa
Tubulin
55KDa
250KDa
150KDa
100KDa
75KDa
55KDa
40KDa
35KDa
250KDa
55KDa

Fig.8

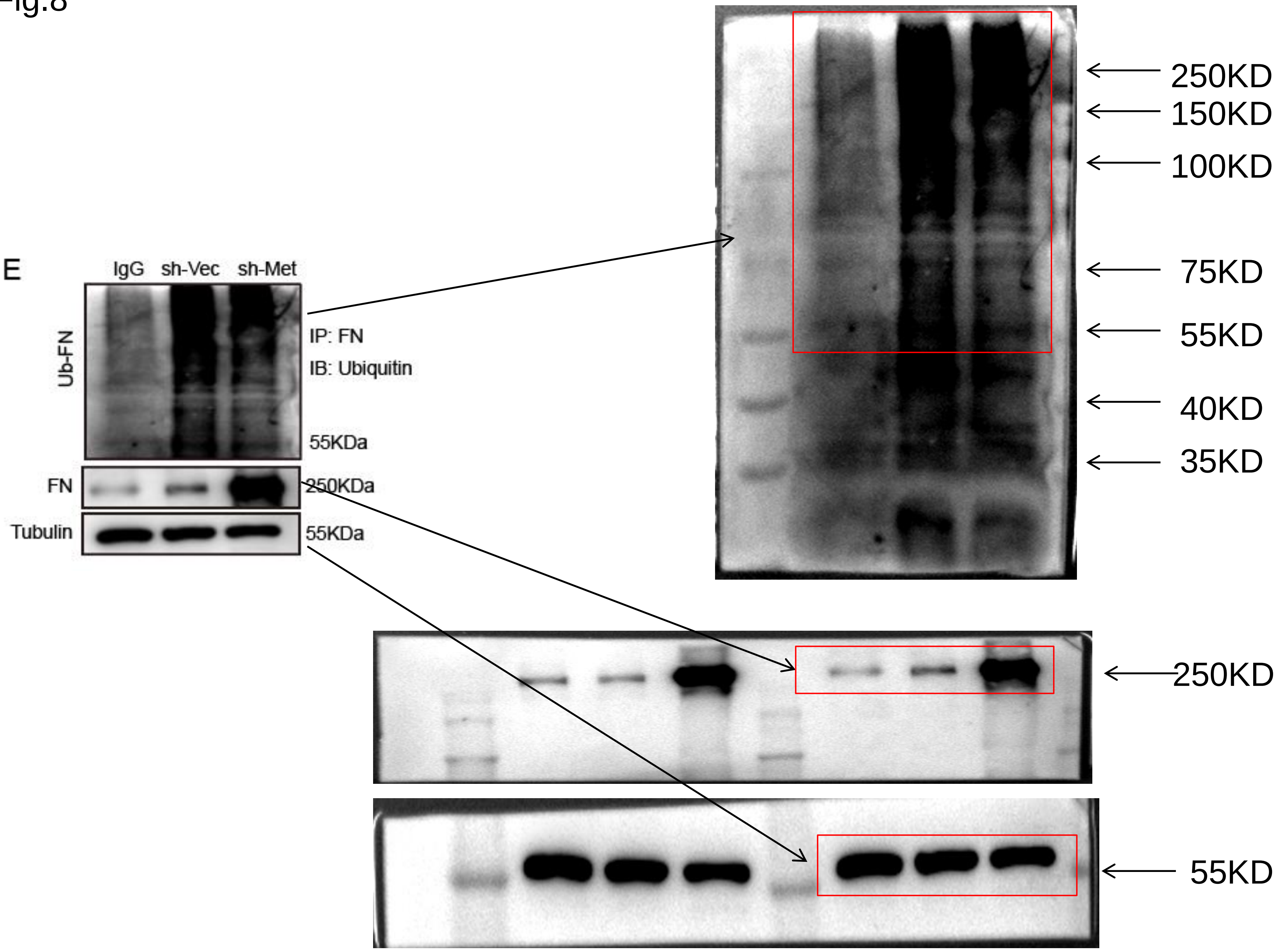
E
IgG
sh-Vec
sh-Met
Ub-FN
IP: FN
IB: Ubiquitin
55KDa
FN
250KDa
Tubulin
55KDa
250KD
150KD
100KD
75KD
55KD
40KD
35KD
250KD
55KD

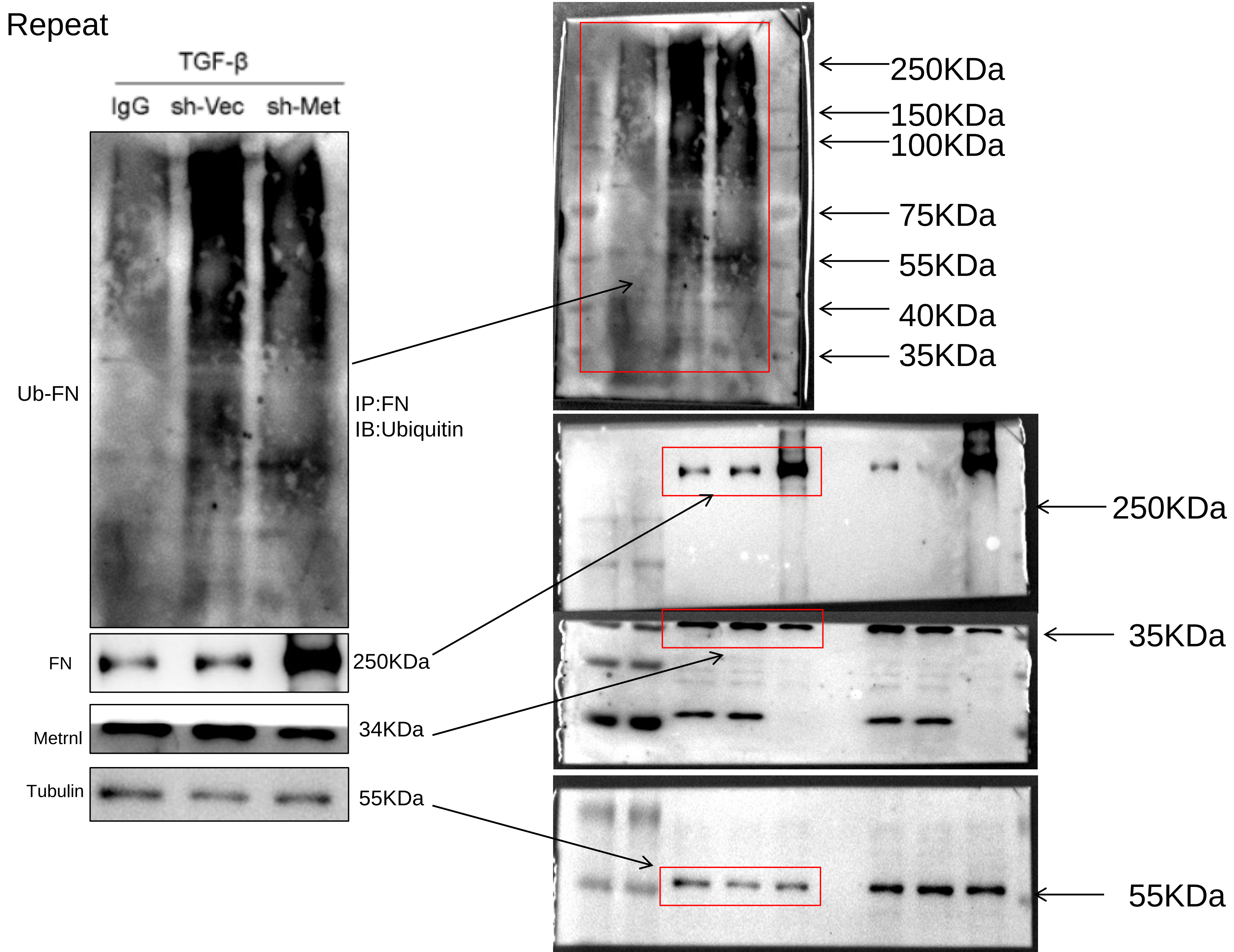
Repeat
TGF-β
IgG
sh-Vec
sh-Met
Ub-FN
IP:FN
IB:Ubiquitin
FN
250KDa
Metrnl
34KDa
Tubulin
55KDa
250KDa
150KDa
100KDa
75KDa
55KDa
40KDa
35KDa
250KDa
35KDa
55KDa

Fig.8

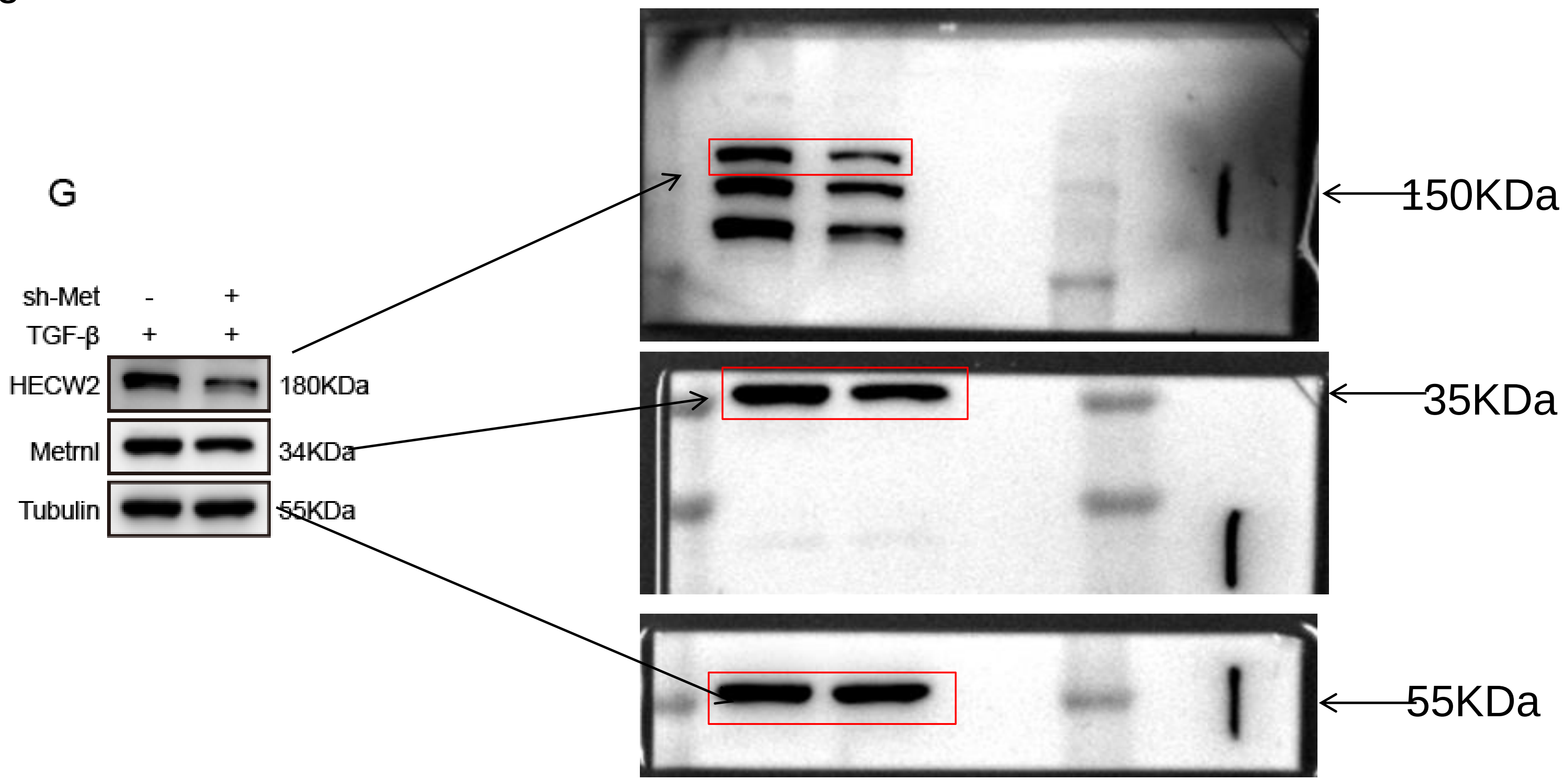
G
sh-Met - +
TGF-β + +
HECW2
180KDa
Metrnl
34KDa
Tubulin
55KDa
150KDa
35KDa
55KDa

Repeat

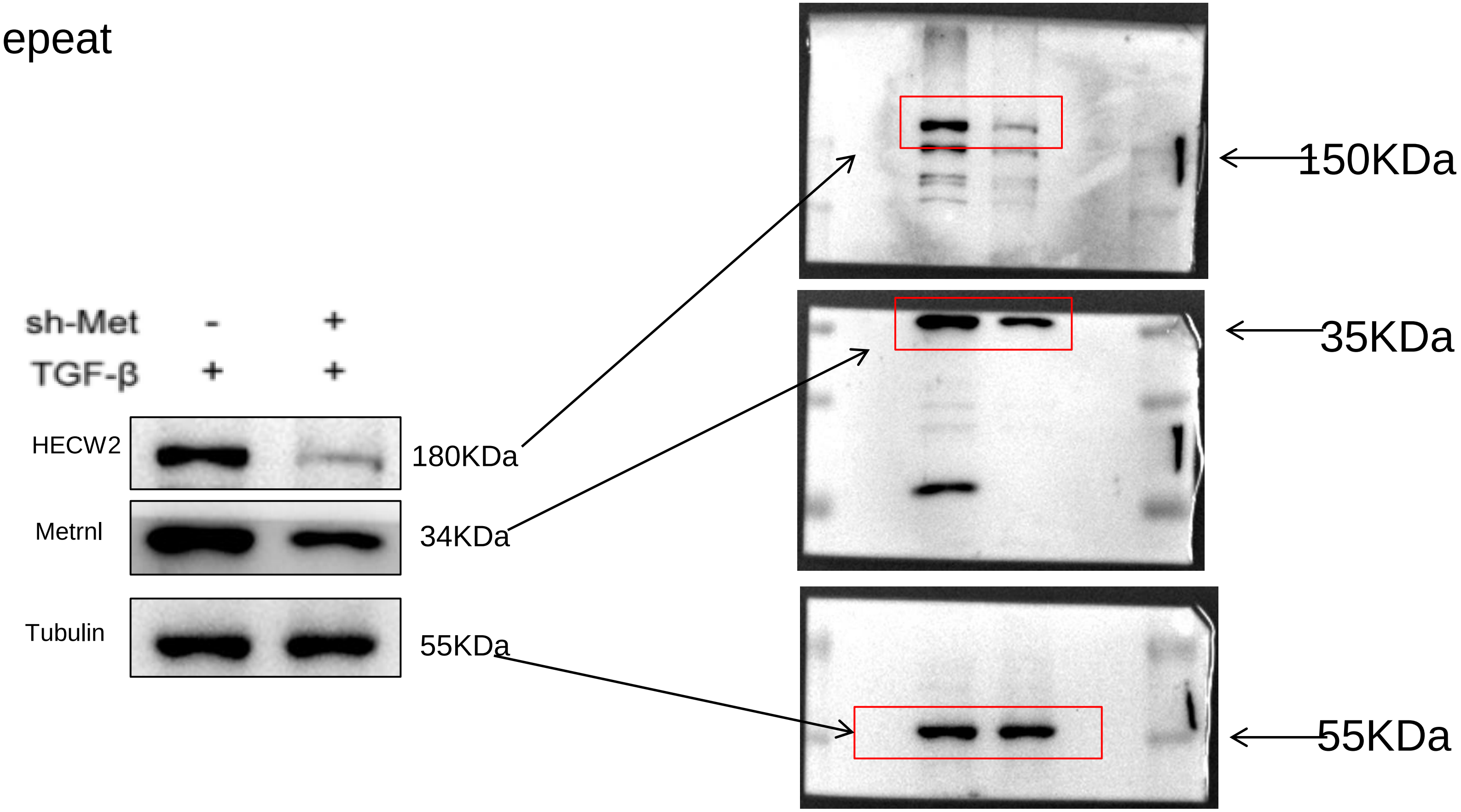
sh-Met - +
TGF-β + +
HECW2
Metrnl
Tubulin
180KDa
34KDa
55KDa
150KDa
35KDa
55KDa

Repeat

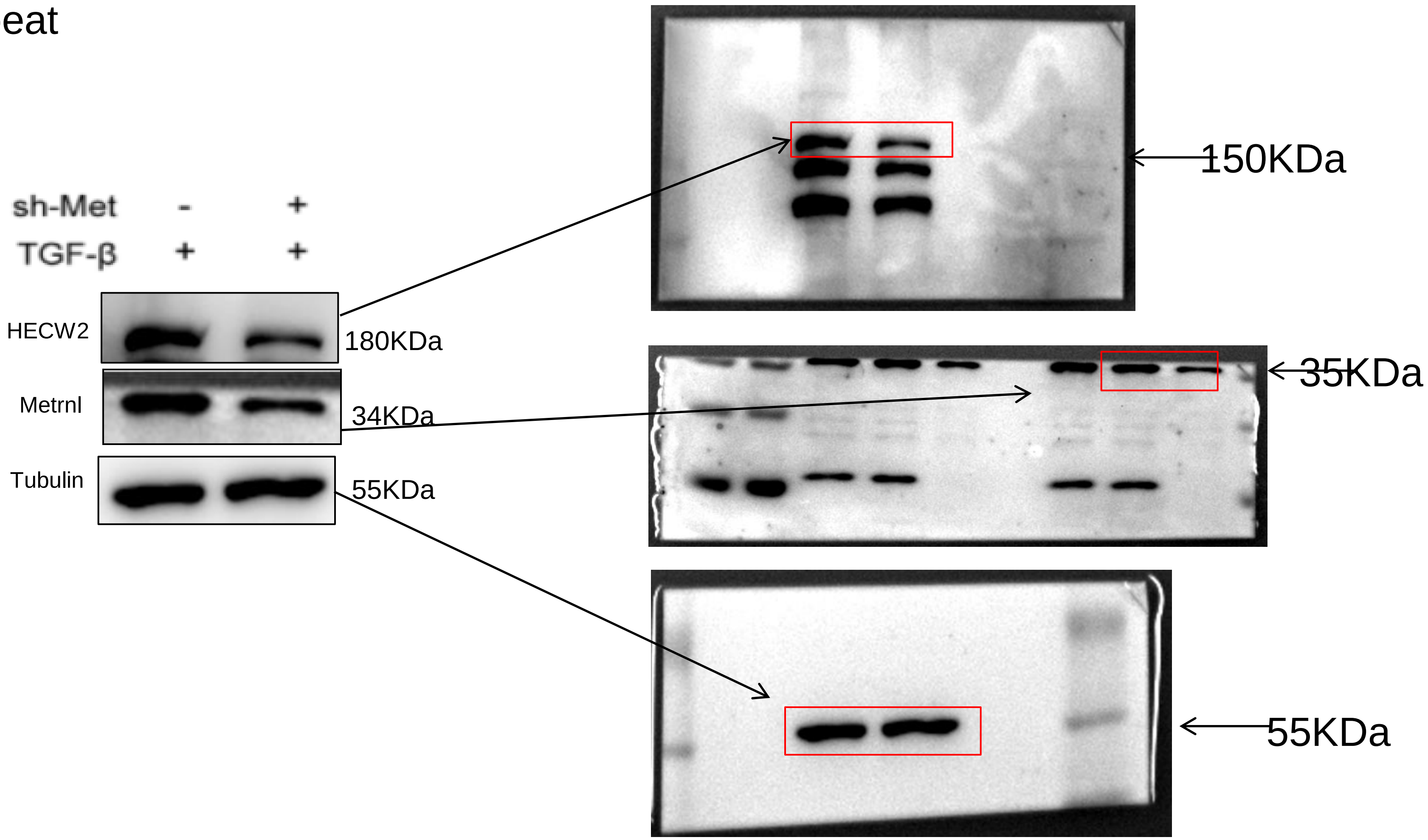
sh-Met - +
TGF-β + +
HECW2
Metrnl
Tubulin
180KDa
34KDa
55KDa
150KDa
35KDa
55KDa

Fig.8

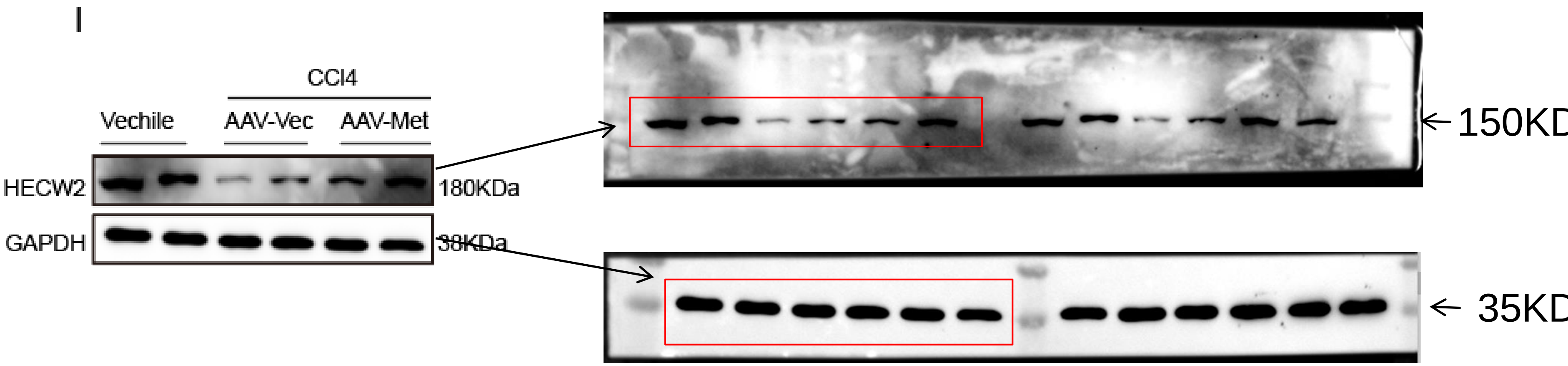
I
CCl4
Vechile
AAV-Vec
AAV-Met
HECW2
180KDa
GAPDH
38KDa
150KD
35KD

Repeat

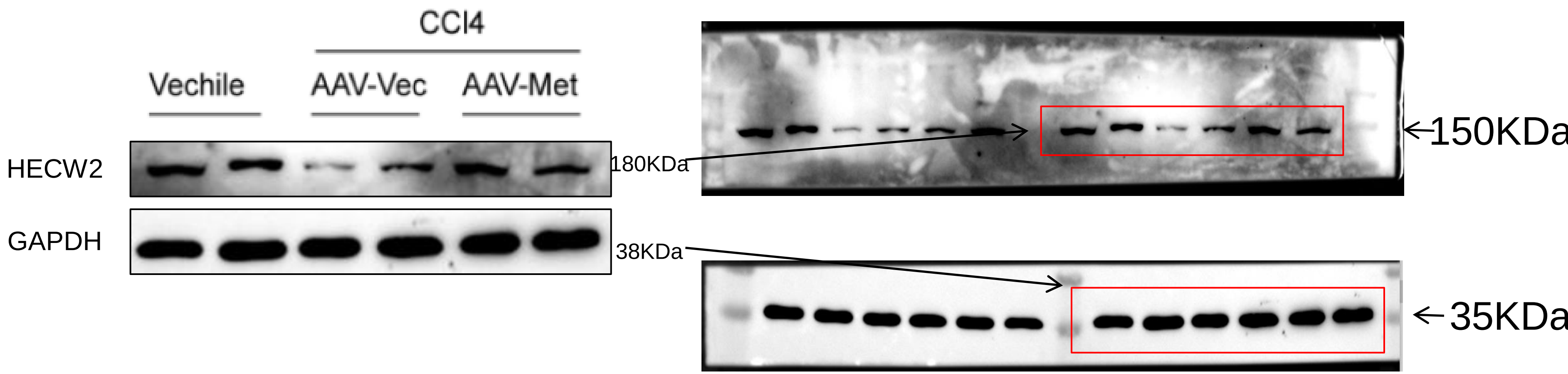

Repeat

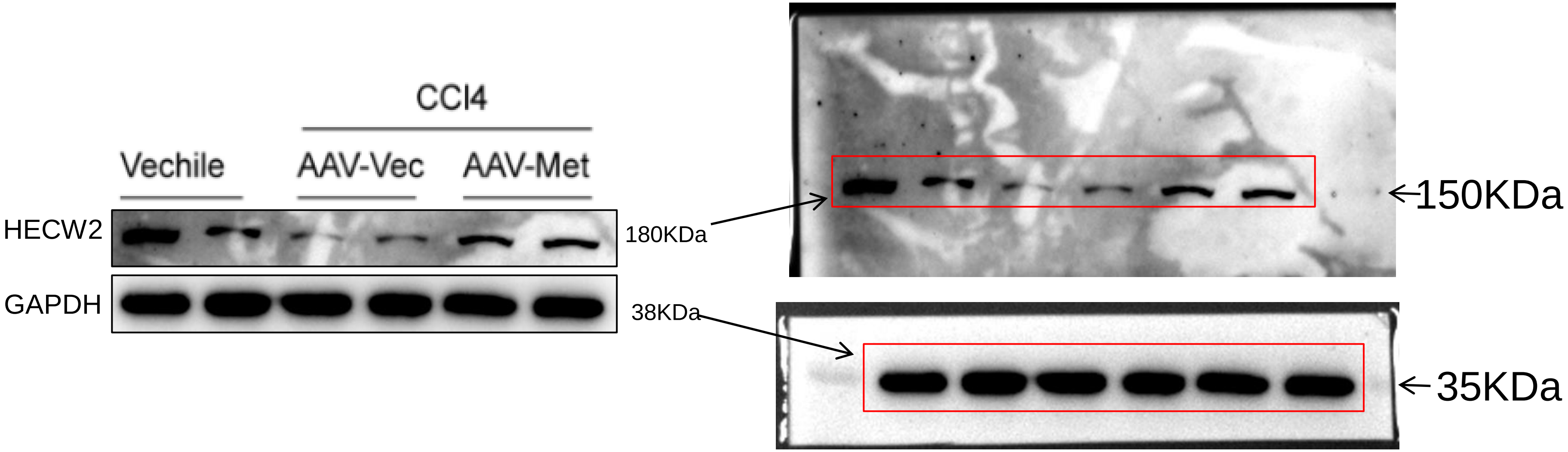

Fig.8

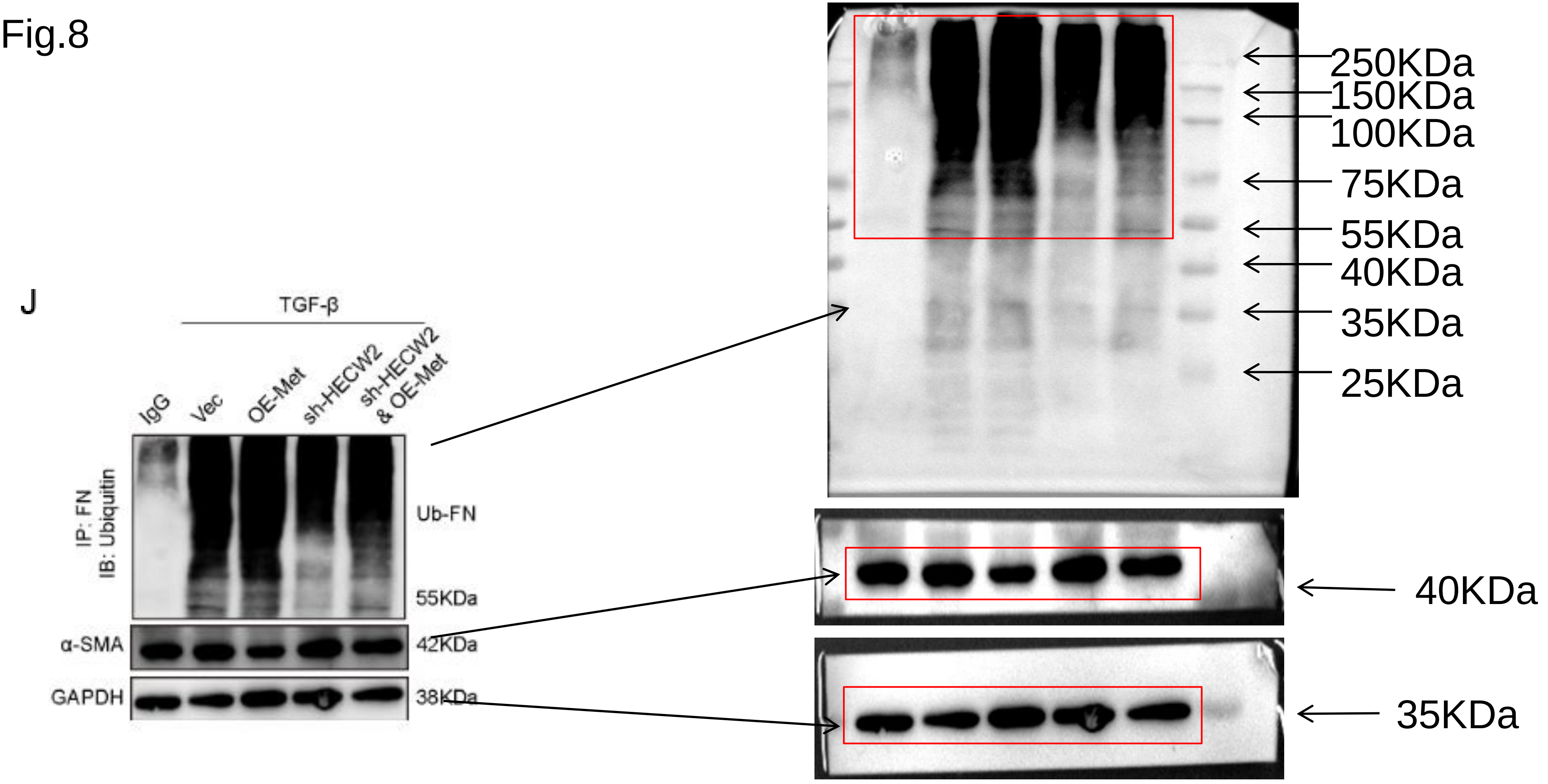
J
TGF-β
IgG
Vec
OE-Met
sh-HECW2
sh-HECW2 & OE-Met
IP: FN
IB: Ubiquitin
Ub-FN
55KDa
α-SMA
42KDa
GAPDH
38KDa
250KDa
150KDa
100KDa
75KDa
55KDa
40KDa
35KDa
25KDa
40KDa
35KDa

Repeat

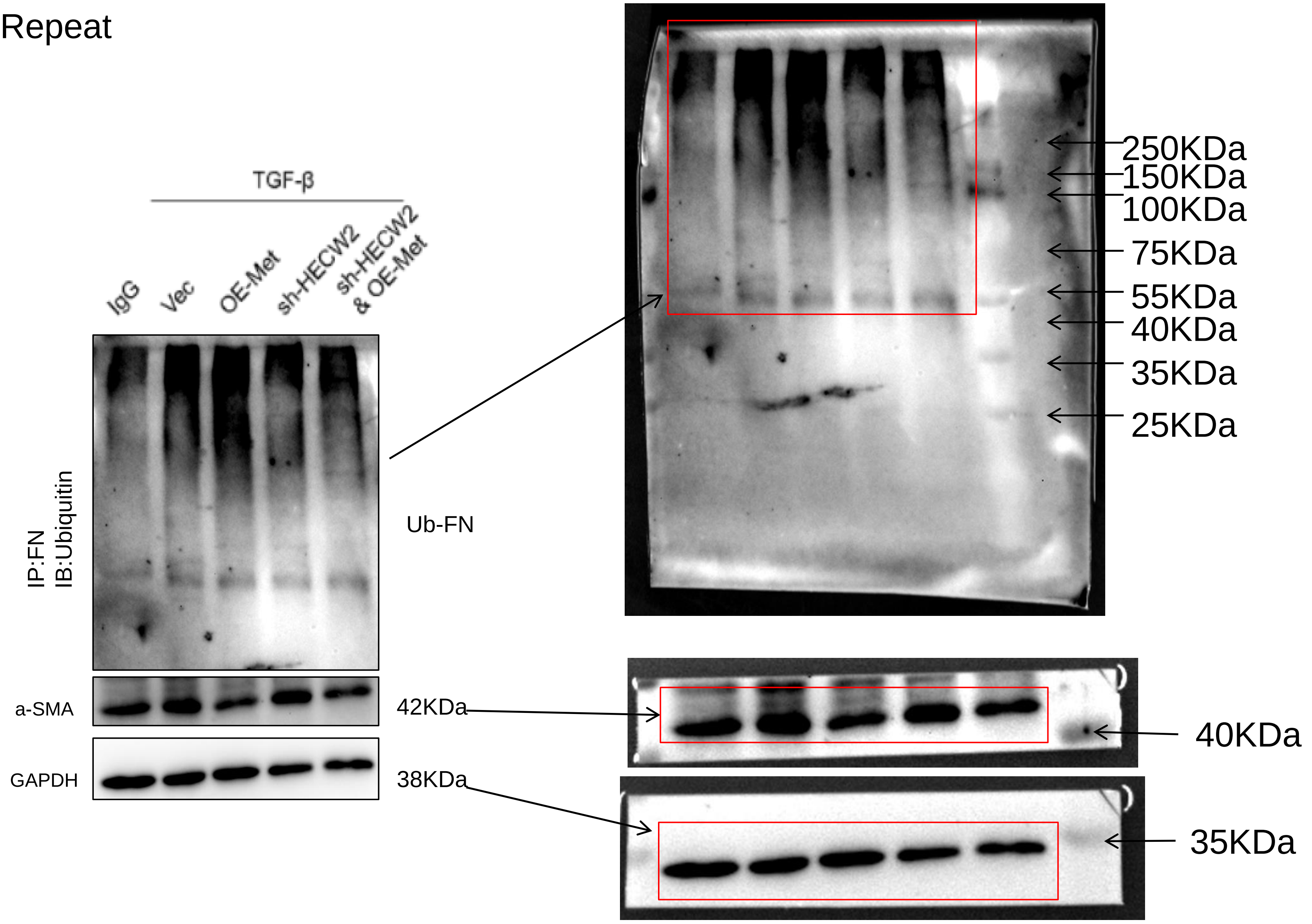

TGF-β
IgG
Vec
OE-Met
sh-HECW2
sh-HECW2 & OE-Met
IP:FN
IB:Ubiquitin
Ub-FN
a-SMA
42KDa
GAPDH
38KDa
250KDa
150KDa
100KDa
75KDa
55KDa
40KDa
35KDa
25KDa
40KDa
35KDa

Fig.8

K

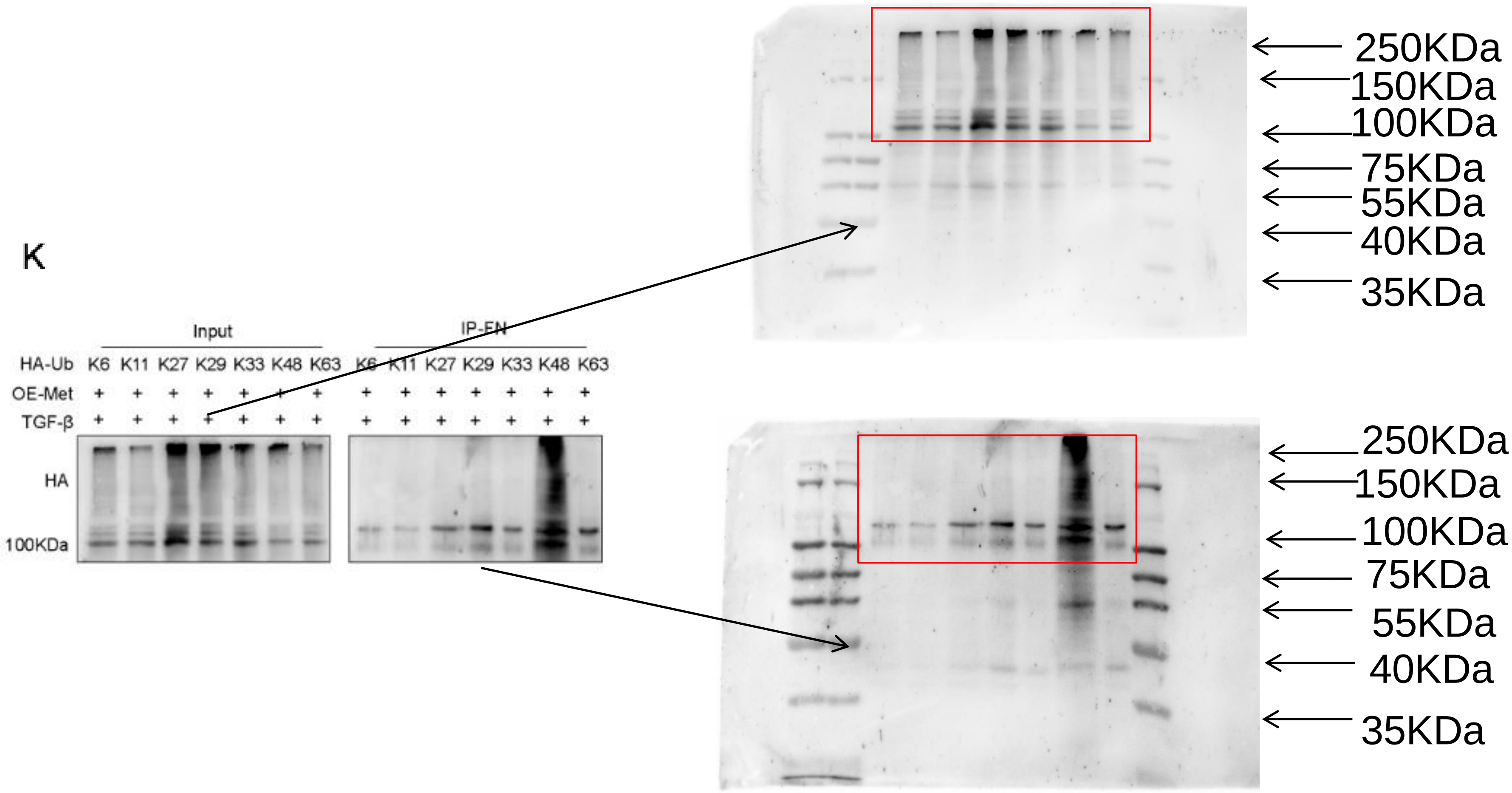
Input
IP-FN
HA-Ub K6 K11 K27 K29 K33 K48 K63 K6 K11 K27 K29 K33 K48 K63
OE-Met + + + + + + + + + + + + + +
TGF-β + + + + + + + + + + + + + +
HA
100KDa
250KDa
150KDa
100KDa
75KDa
55KDa
40KDa
35KDa
250KDa
150KDa
100KDa
75KDa
55KDa
40KDa
35KDa

Repeat

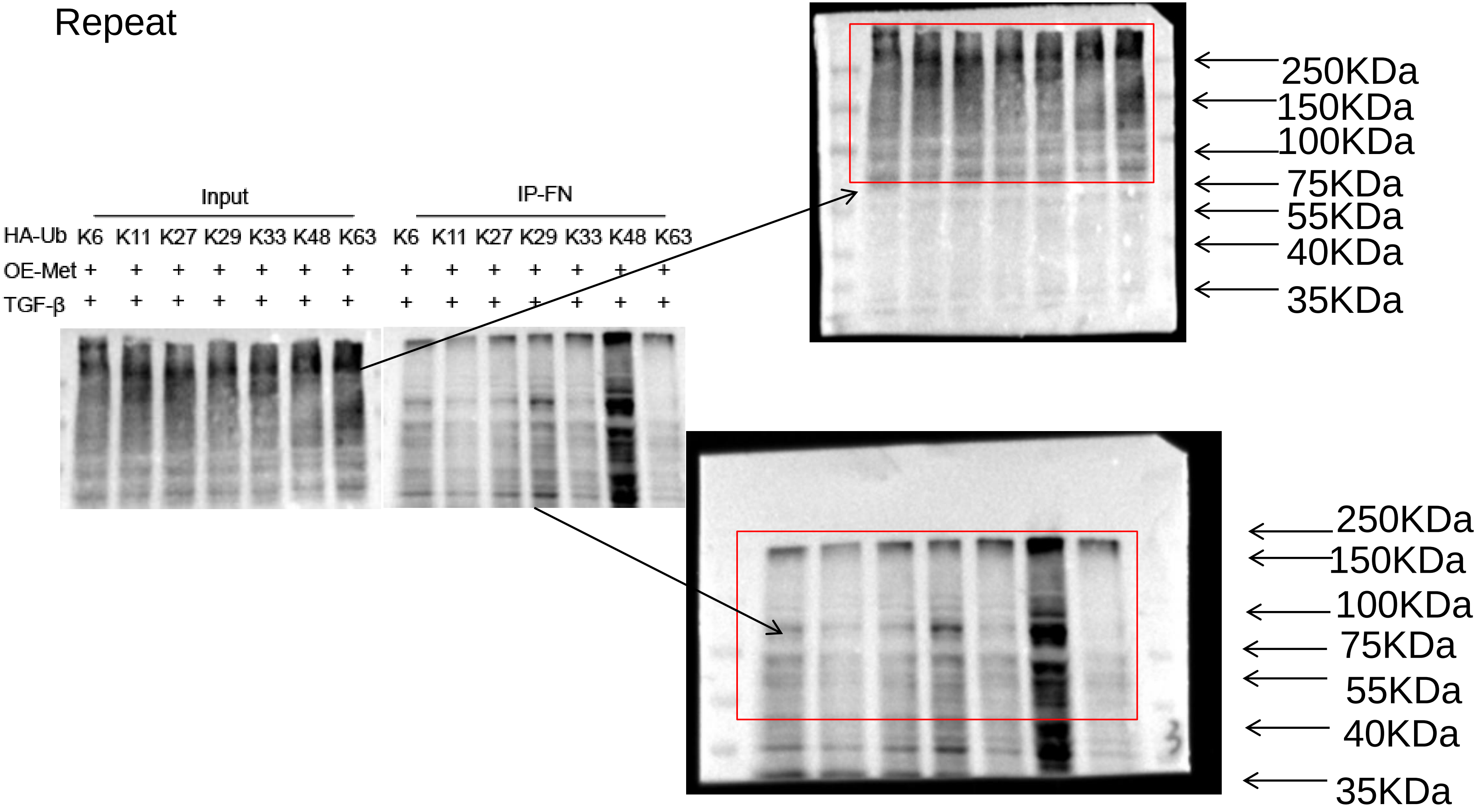

Input
IP-FN
HA-Ub K6 K11 K27 K29 K33 K48 K63 K6 K11 K27 K29 K33 K48 K63
OE-Met + + + + + + + + + + + + + +
TGF-β + + + + + + + + + + + + + +
250KDa
150KDa
100KDa
75KDa
55KDa
40KDa
35KDa
250KDa
150KDa
100KDa
75KDa
55KDa
40KDa
35KDa

Repeat

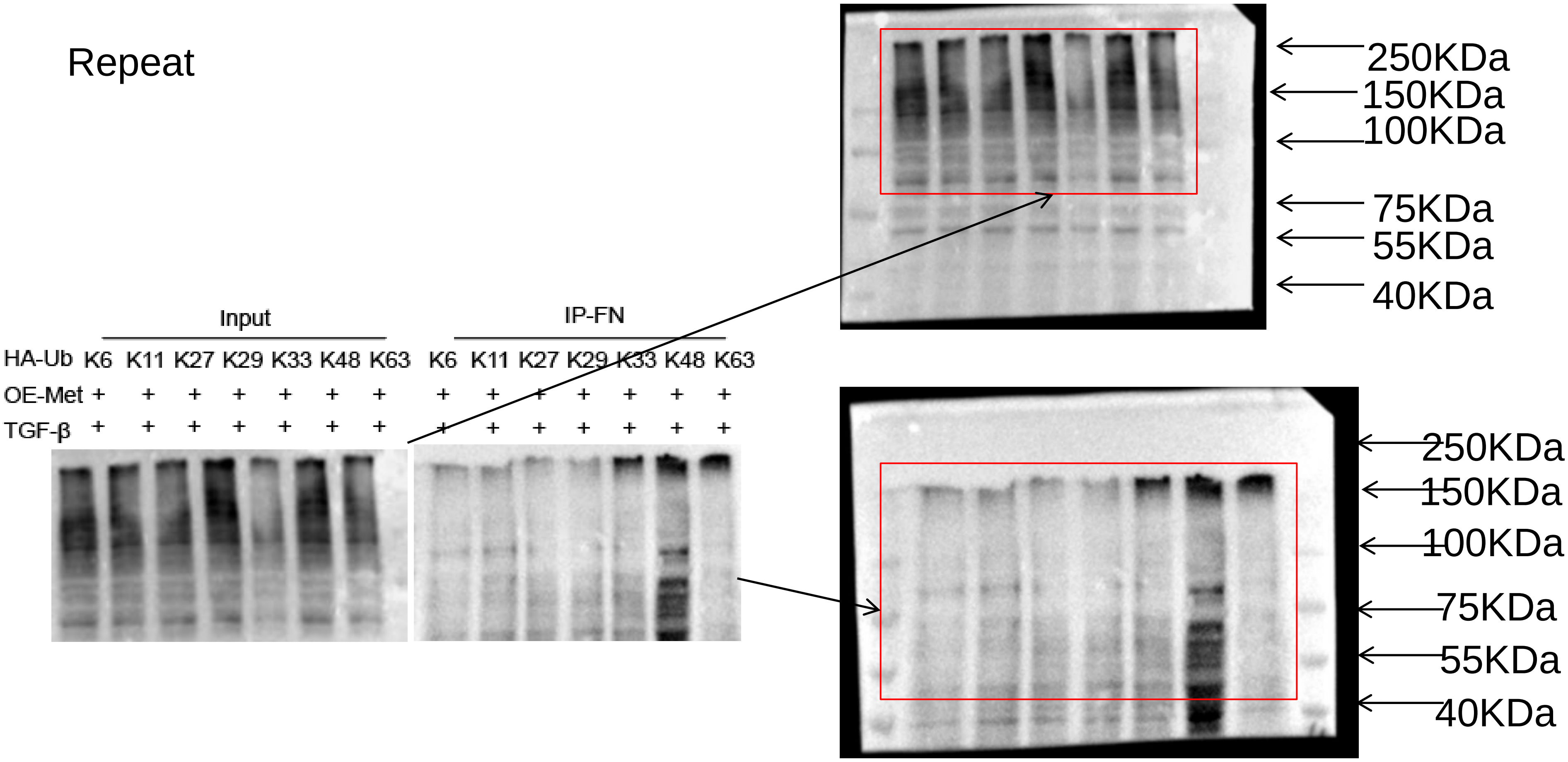

Input
IP-FN
HA-Ub K6 K11 K27 K29 K33 K48 K63 K6 K11 K27 K29 K33 K48 K63
OE-Met + + + + + + + + + + + + + +
TGF-β + + + + + + + + + + + + + +
250KDa
150KDa
100KDa
75KDa
55KDa
40KDa
250KDa
150KDa
100KDa
75KDa
55KDa
40KDa

Supplementary Fig.1

A

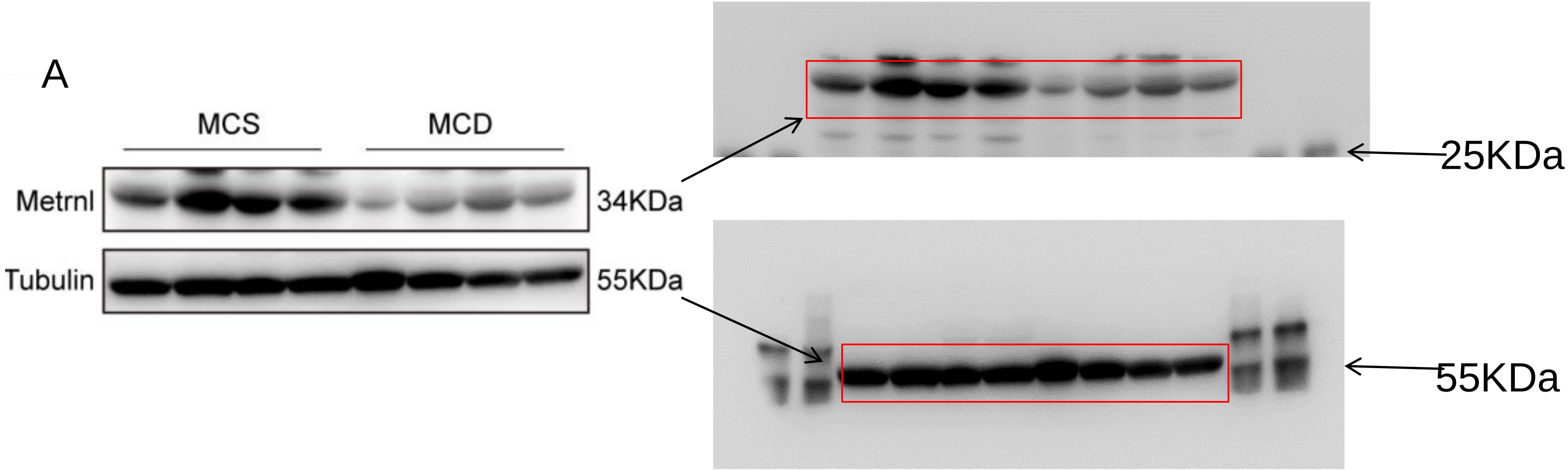

Supplementary Fig.1

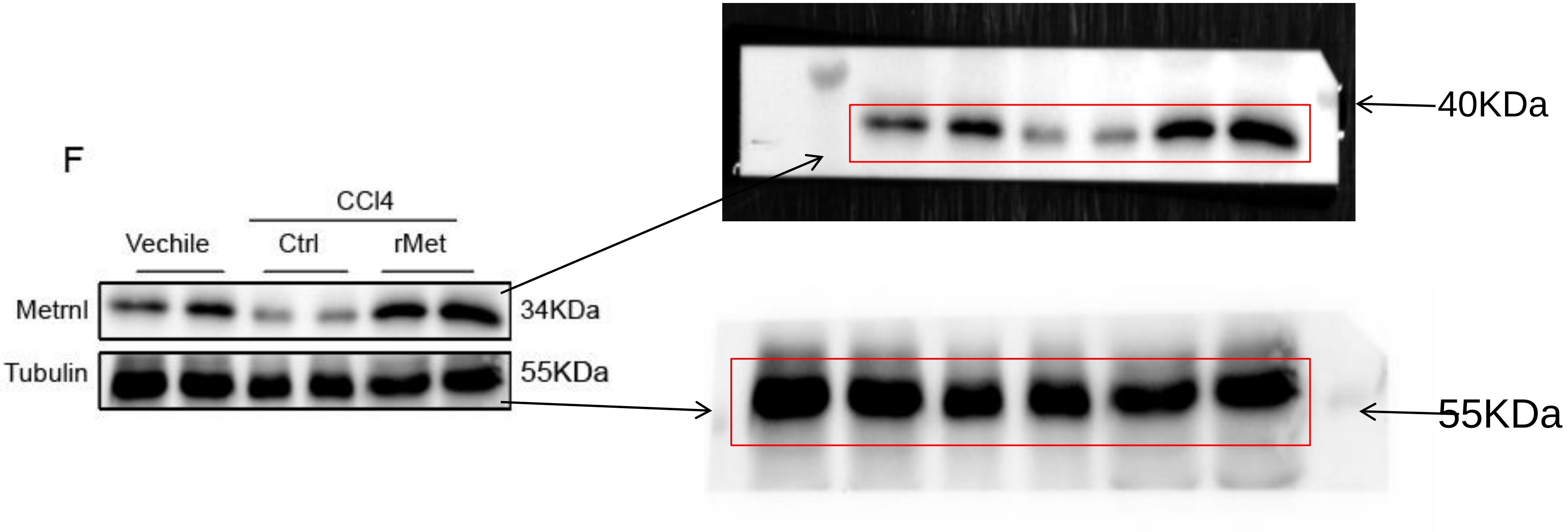

Repeat

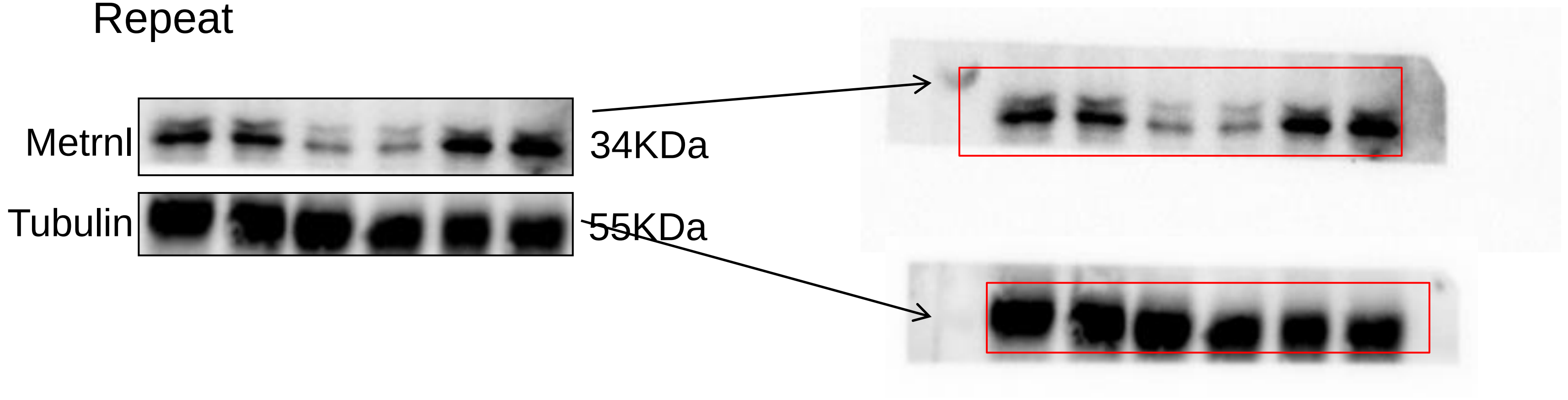

Repeat

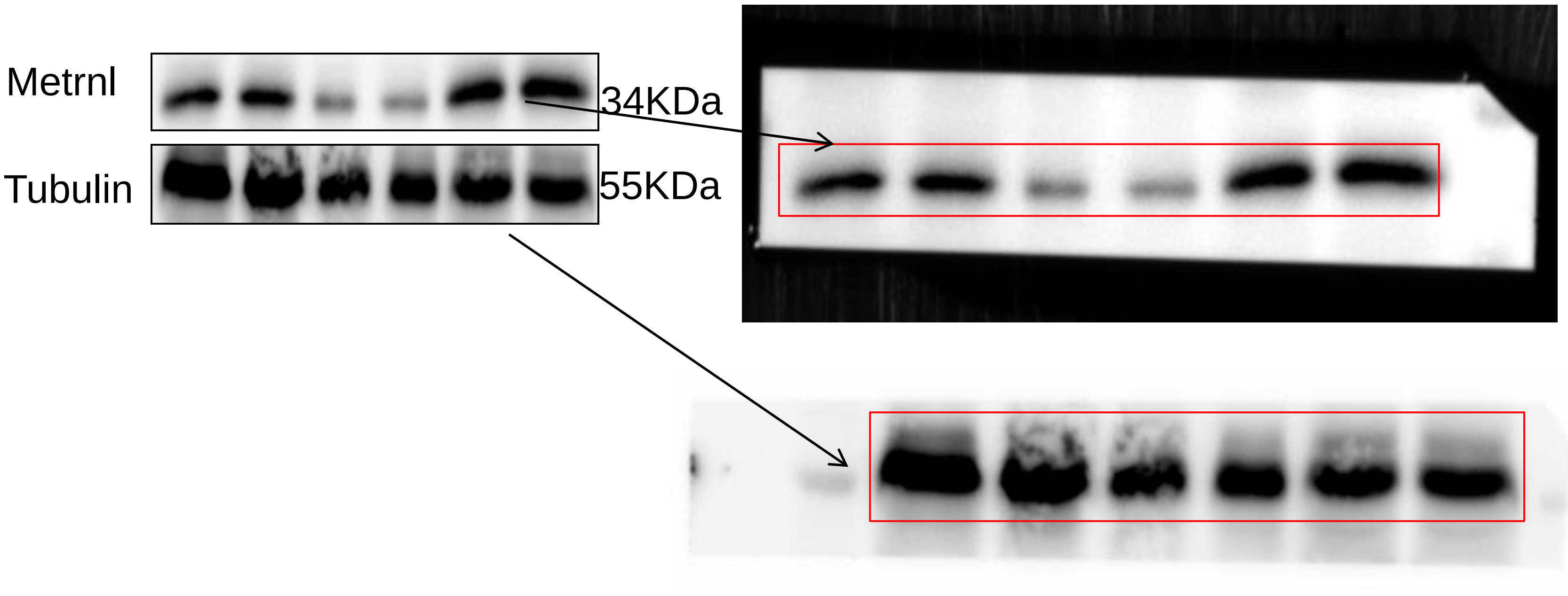

Supplementary Fig.1

I

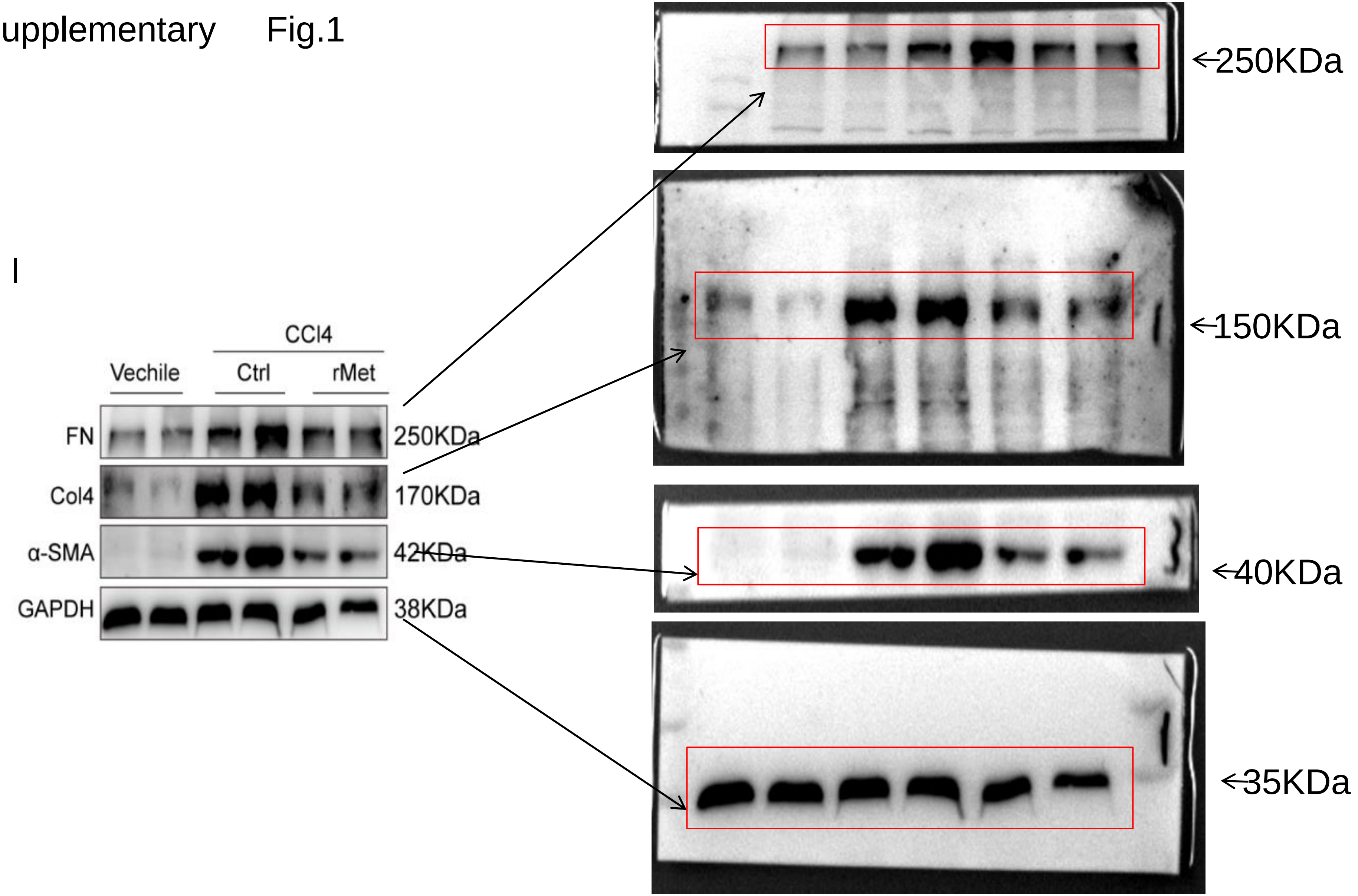

Repeat

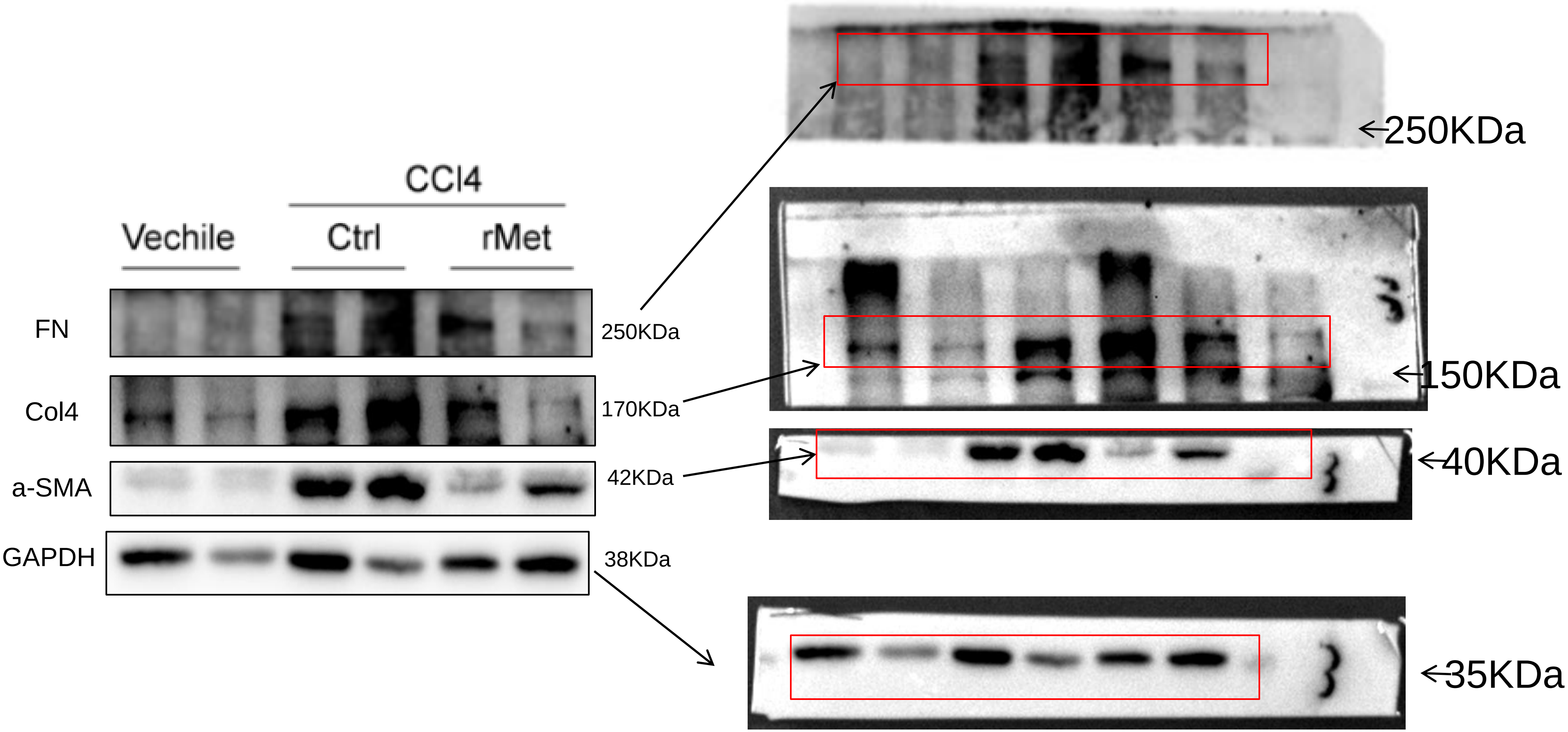
CCl4
Vechile
Ctrl
rMet
FN
Col4
a-SMA
GAPDH
250KDa
170KDa
42KDa
38KDa
250KDa
150KDa
40KDa
35KDa

Repeat

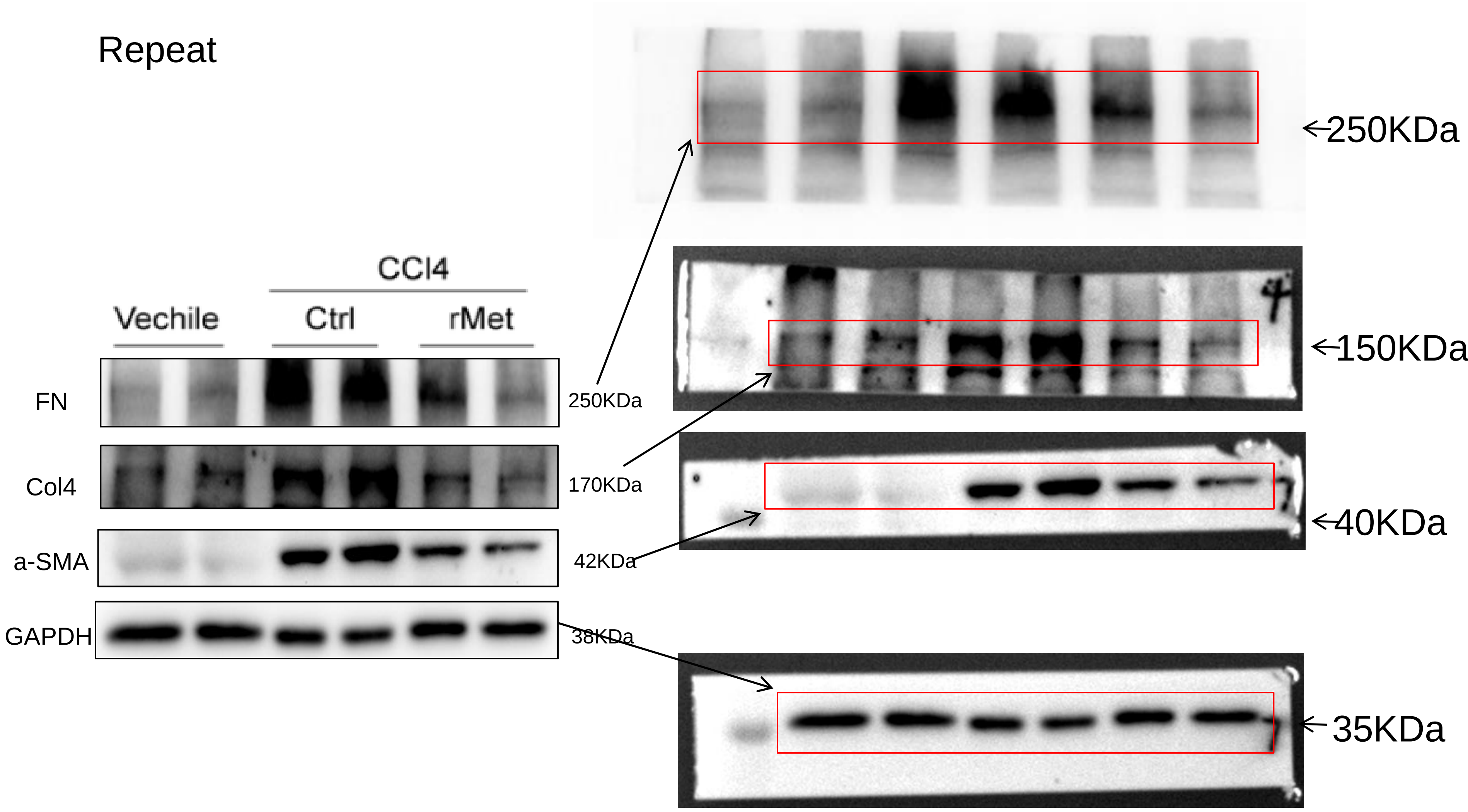

CCl4
Vechile
Ctrl
rMet
FN
Col4
a-SMA
GAPDH
250KDa
170KDa
42KDa
38KDa
250KDa
150KDa
40KDa
35KDa

Supplementary Fig.1

J

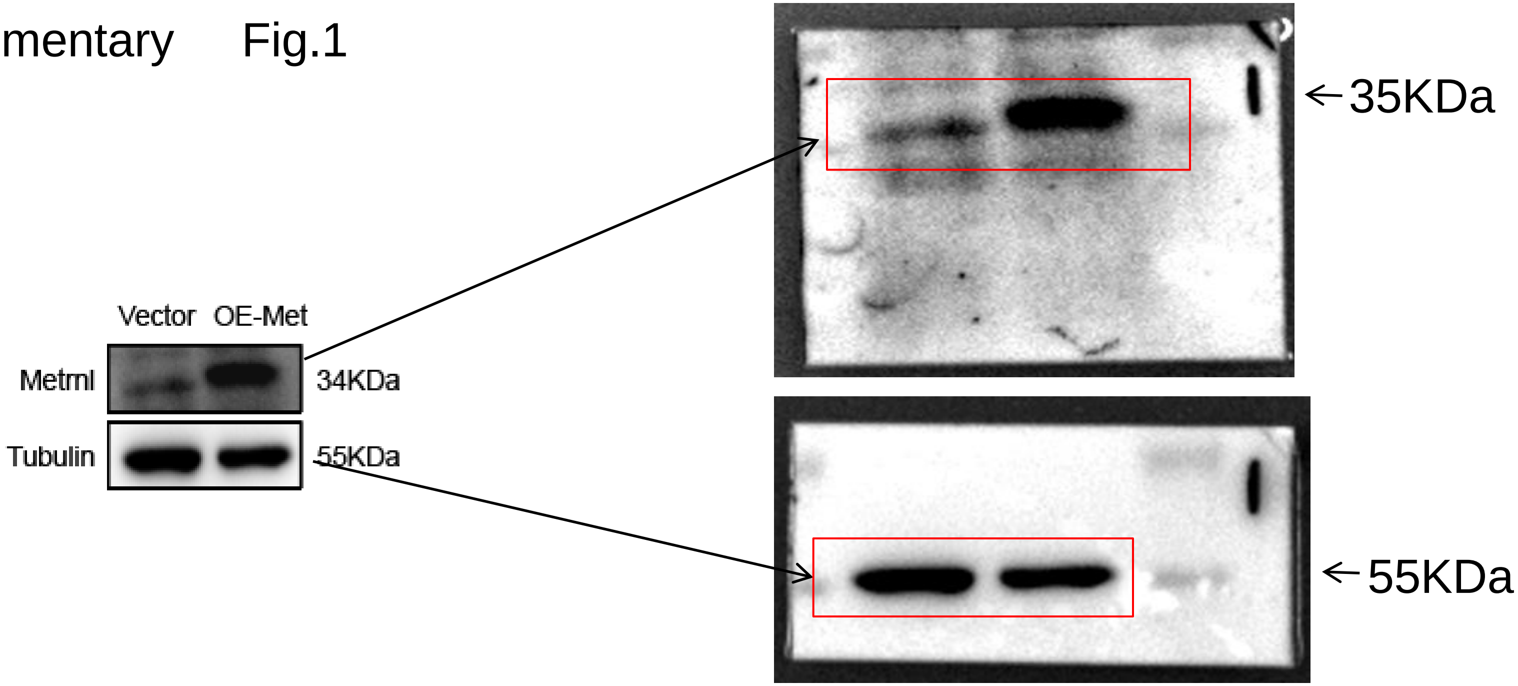

Repeat

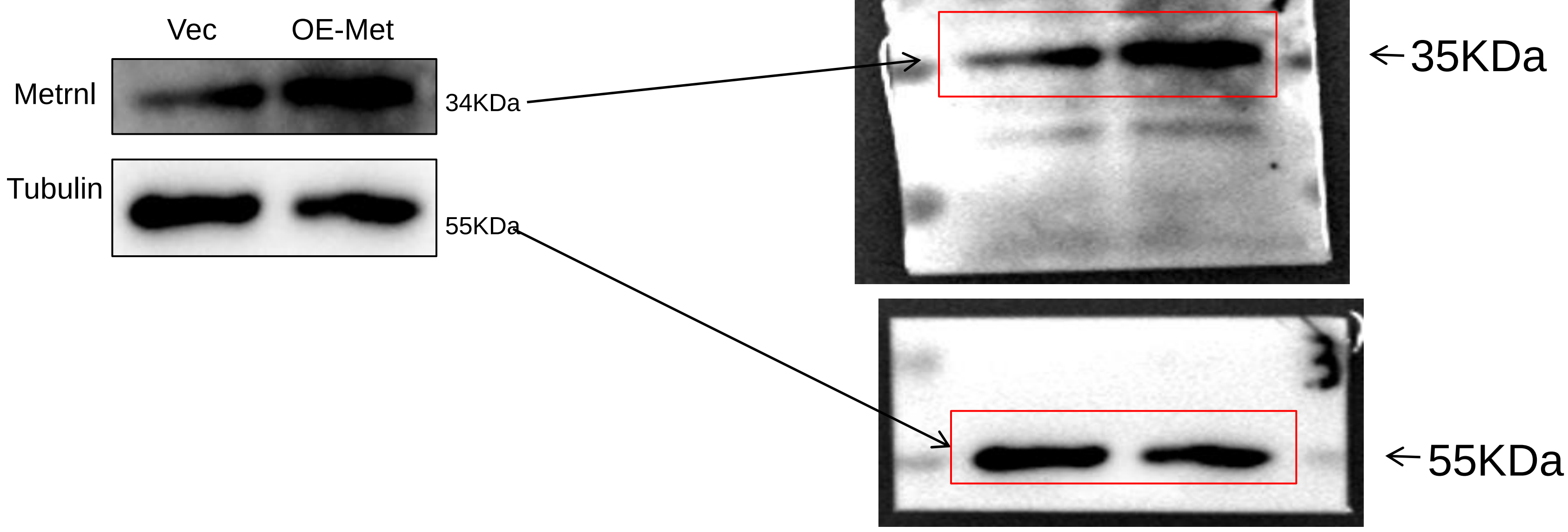

Repeat

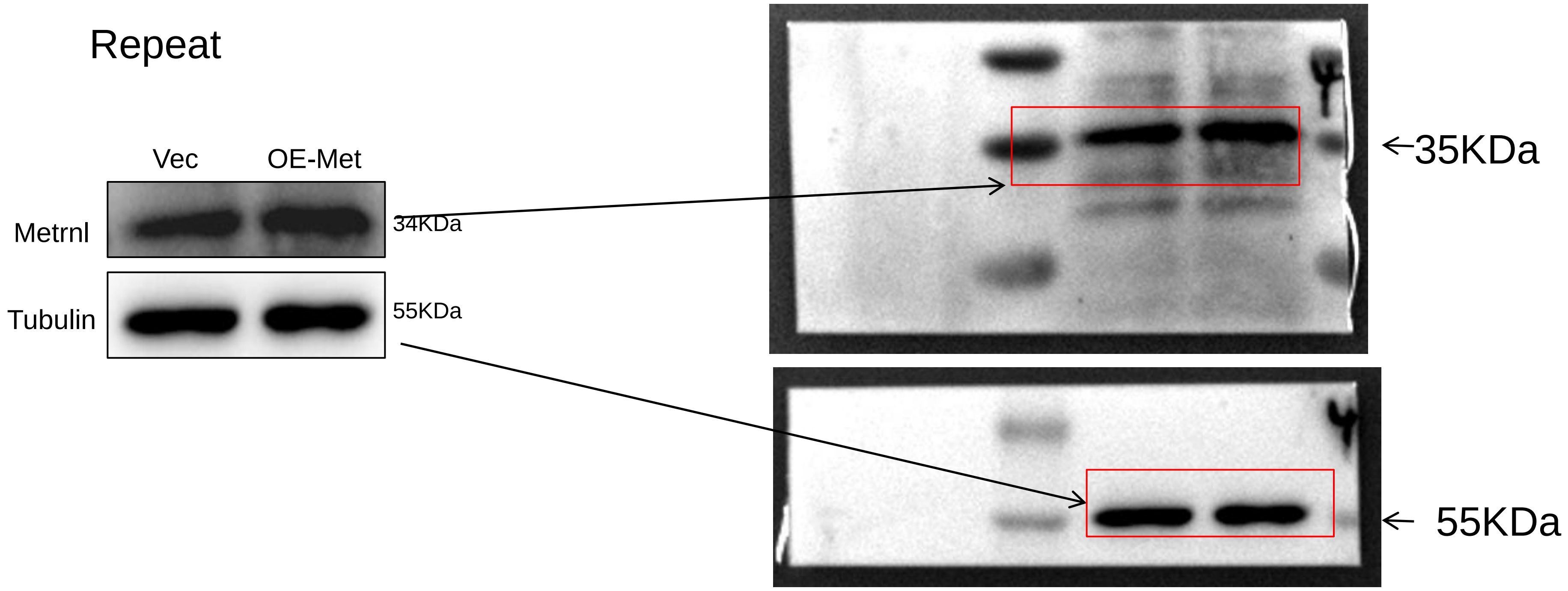

Supplementary Fig.4

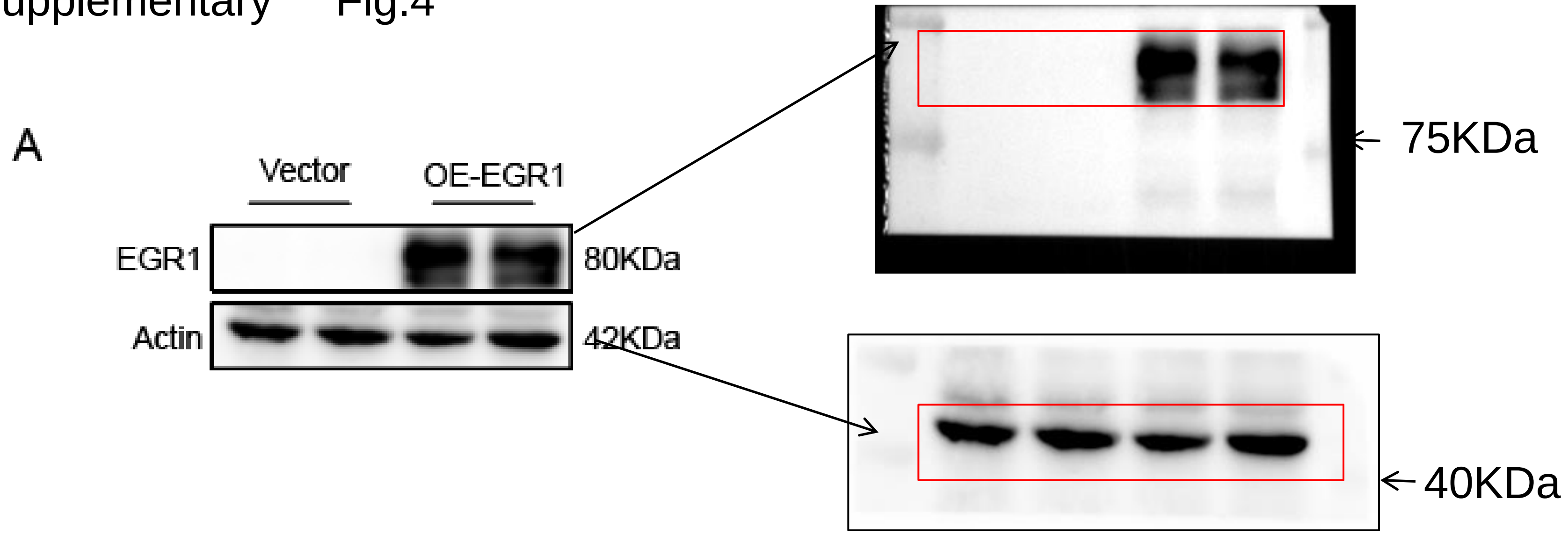

Repeat

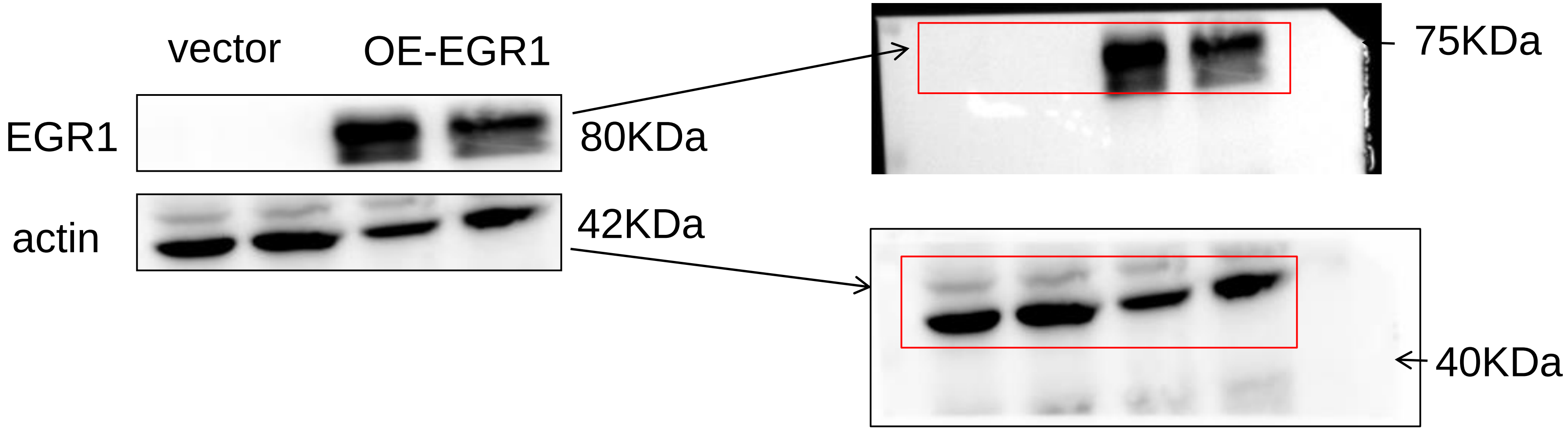

Repeat

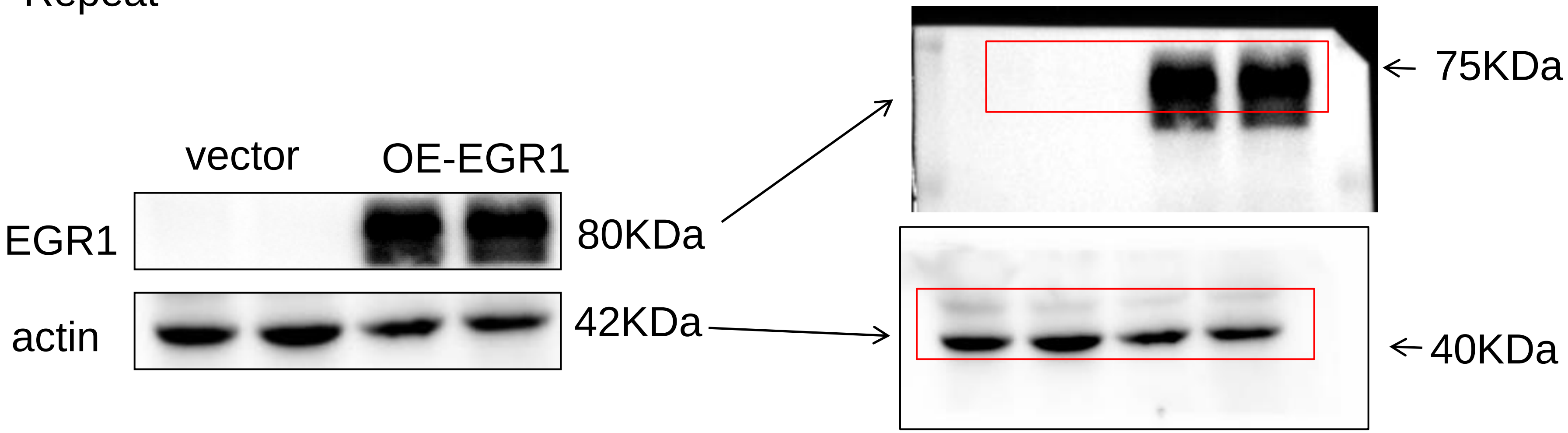

Supplementary Fig.4

B

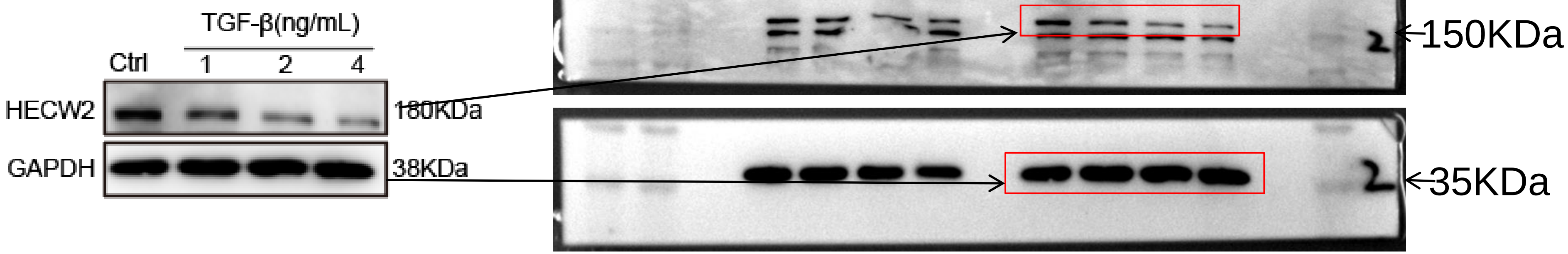

Repeat

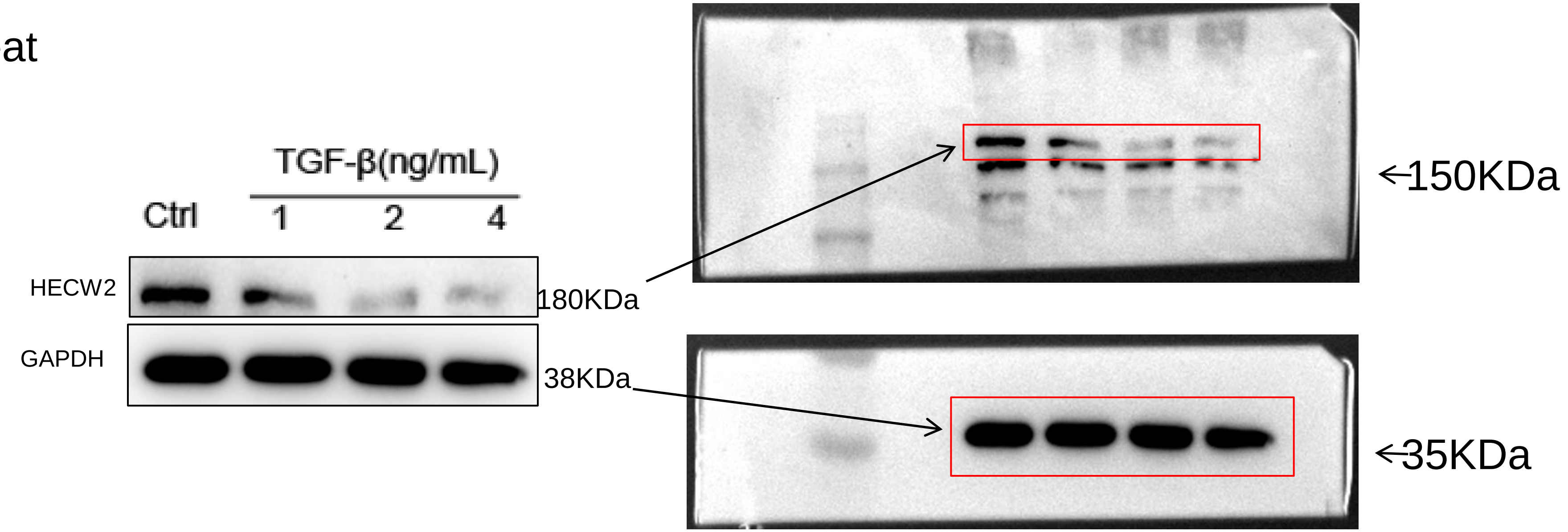

Repeat

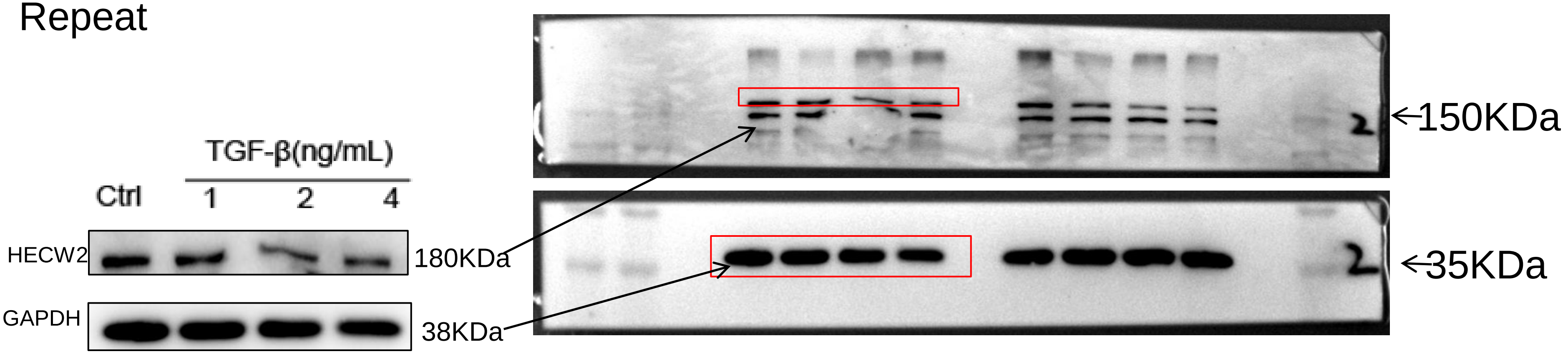

Supplement: Supplementary file 7 — Western blot Original Data [file 41419_2025_7734_MOESM7_ESM.pdf]
